# Can posttreatment blood inflammatory markers predict poor survival in gynecologic cancer?: a systematic review and meta-analysis

**DOI:** 10.3389/fimmu.2025.1676838

**Published:** 2025-10-21

**Authors:** Minyong Choi, Sea-Won Lee, Woohyun Park, Young Sub Lee, Seok Ho Lee, Jong Hoon Lee, Tiara Bunga Mayang Permata, Kwangil Yim

**Affiliations:** ^1^ College of Medicine, The Catholic University of Korea, Seoul, Republic of Korea; ^2^ Department of Radiation Oncology, Eunpyeong St. Mary’s Hospital, College of Medicine, The Catholic University of Korea, Seoul, Republic of Korea; ^3^ Department of Data Science, The Catholic University of Korea, Bucheon, Gyeonggi-do, Republic of Korea; ^4^ Department of Hospital Pathology, Eunpyeong St. Mary’s Hospital, College of Medicine, The Catholic University of Korea, Seoul, Republic of Korea; ^5^ Department of Radiation Oncology, Gachon University Gil Medical Center, Incheon, Republic of Korea; ^6^ Department of Radiation Oncology, St. Vincent’s Hospital, College of Medicine, The Catholic University of Korea, Seoul, Republic of Korea; ^7^ Department of Radiation Oncology, Cipto Mangunkusumo National General Hospital - Faculty of Medicine Universitas Indonesia, Jakarta, Indonesia; ^8^ Department of Hospital Pathology, Uijeongbu St. Mary’s Hospital, College of Medicine, The Catholic University of Korea, Seoul, Republic of Korea

**Keywords:** systematic reviews, genital neoplasms female, peripheral blood inflammatory marker, neutrophil lymphocyte ratio, posttreatment, dynamic change

## Abstract

**Introduction:**

Peripheral blood inflammatory markers (PBIMs) are widely used for prognostication of several malignancies, including gynecologic cancers. However, most studies do not report when PBIMs have been sampled, and the ones that do usually use pretreatment levels. Considering their potential to reflect the host immune status, posttreatment PBIMs and their dynamic changes from pretreatment levels may also carry prognostic information. A systematic review and meta-analysis were conducted to identify the prognostic value of posttreatment PBIMs and their dynamic changes from baseline in gynecologic cancers. Furthermore, among the inconsistent blood draw timing and analytical methods, we aimed to suggest the most suitable strategies in the clinical setting.

**Methods:**

Fourteen eligible studies comprising 2,373 patients with cervical, ovarian, or endometrial cancer were included. The associations between survival outcomes, including overall survival (OS), progression-free survival (PFS), and disease-free survival (DFS), and the PBIMs were extracted or estimated. The PBIMs included the neutrophil-to-lymphocyte ratio (NLR), the platelet-to-lymphocyte ratio (PLR), the monocyte-to-lymphocyte ratio (MLR), the systemic immune-inflammation index (SII), and the systemic inflammation response index (SIRI). Subgroup analyses examined early versus late posttreatment sampling, as well as dynamic assessments based on threshold-defined change (increase or decrease) versus simple directional change (high or low).

**Results:**

All PBIMs (NLR, PLR, MLR, SII, and SIRI) demonstrated significant association with relevant survival endpoints (OS, PFS, and DFS). Early sampling of within one month after treatment completion (≤ median 15 days) showed prognostic significance (pooled hazard ratios 3.43–3.55; p < 0.0001), whereas late sampling demonstrated no significant associations. Dynamic classification using specific thresholds yielded more consistent and less heterogeneous estimates than directionality-based approaches.

**Discussion:**

This meta-analysis demonstrates the prognostic potential of posttreatment PBIMs and their dynamic change from baseline in gynecologic cancers. Sampling within one month after therapy was significantly associated with prognosis, which may reflect the importance of sampling time in relation to the different recovery times by immune cell compartments. However, considering the heterogeneity of confounders between studies, the results should be interpreted with caution. These findings warrant the need for further studies to standardize PBIM assessment in clinical practice.

## 1 Introduction

Gynecologic cancer remains a major contributor to cancer-related mortality worldwide (1). Despite the availability of effective treatments, including adjuvant chemotherapy and targeted therapies (2), therapeutic outcomes remain suboptimal (3, 4), underscoring the need for reliable prognostic biomarkers to guide individualized treatment strategies.

Systemic inflammation has gained increasing attention due to its pivotal role in cancer initiation, progression, and metastasis (5). Tumor-infiltrating lymphocytes are recognized as prognostic biomarkers and may complement conventional indicators such as stage and grade (6). Both local immune responses and systemic inflammation are associated with disease progression and poor prognosis (7). Notably, low-grade chronic inflammation, characterized by sustained immune activation and elevated levels of proinflammatory mediators, often precedes cancer onset and may contribute to tumorigenesis (8).

Pre-treatment systemic inflammation is commonly assessed using peripheral blood inflammatory markers (PBIMs), including neutrophil-to-lymphocyte ratio (NLR) (9), platelet-to-lymphocyte ratio (PLR) (10), monocyte-to-lymphocyte ratio (MLR) (11), systemic inflammation response index (SIRI) (12), and systemic immune-inflammation index (SII) (13). Elevated pre-treatment PBIMs are well-established prognostic indicators in various cancers, particularly gynecologic malignancies, and have been extensively explored in systematic reviews (14–18).

In contrast, relatively few studies have investigated the prognostic significance of posttreatment PBIMs or their dynamic changes relative to pre-treatment levels. Recent findings suggest that posttreatment PBIMs, evaluated independently or as dynamic shifts, may also possess prognostic relevance (19–32). Restoration of immunocompetence after various treatment-related effects such as surgical injury (33), adjuvant chemotherapy, or radiotherapy-related hematopoietic stress is considered important for host defense and antitumor immunity (34). Although it is often considered that innate cell counts (e.g. neutrophils, natural killer cells and monocytes) recover relatively more rapidly than those of T lymphocytes, this remains a hypothetical concept (34). In this review, PBIMs are interpreted primarily as indirect indices that may reflect posttreatment immune suppression and reconstitution, rather than as direct evidence of underlying mechanisms. Nevertheless, posttreatment PBIMs may still bear the potential of revealing the host’s immune status per se, in addition to its resilience to treatment-related toxicities, as can be demonstrated by the dynamics between pre- and posttreatment PBIMs (34). Additionally, standardized criteria for the optimal timing of posttreatment PBIM measurements and methods for assessing dynamic changes remain to be established.

This study aimed to elucidate the prognostic significance of posttreatment and dynamic PBIMs in gynecologic cancers. Additionally, we anticipated to suggest standardized criteria for posttreatment sampling time and dynamic assessment methods for practical application in clinical settings.

## 2 Methods

### 2.1 Search strategy

This meta-analysis was submitted to PROSPERO (No. 453021) and approved by the institutional review board (IRB No. UC23ZISI0108), with full accreditation by the Association for the Accreditation of Human Research Protection Programs (AAHRPP). Three major electronic databases, Medline, EMBASE, and the Cochrane Library, were searched for relevant articles in English language published up to September 13, 2024. The search terminologies and deviations from PROSPERO are summarized in **Supplementary Tables S1, S2** respectively. Additionally, the reference lists of key articles were manually screened to identify further eligible studies. The authors of the PBIM studies that were lacking HR data were contacted. EndNote X20 (Build 10136; Thomson Reuters, New York, NY, USA) was used to manage the retrieved records.

### 2.2 Inclusion and exclusion criteria

The following inclusion criteria were applied in this meta-analysis: (1) studies reporting sufficient information on hazard ratios (HRs) for patient survival; (2) studies evaluating the association between post-treatment PBIMs, or dynamic changes in PBIMs before and after treatment, and prognosis; (3) studies examining the relationship between PBIMs and clinicopathological features; and (4) articles written in English language. The exclusion criteria were as follows: (1) studies reporting only pre-treatment PBIMs; (2) duplicate publications, reviews, case reports, letters, and conference proceedings; (3) studies lacking an association between PBIMs and survival or clinicopathological parameters; (4) studies involving cancer cell lines or animal models; and (5) studies with insufficient data on HRs and 95% confidence intervals (CIs) that could not be extracted or calculated.

### 2.3 Data extraction and assessment of study quality

Data extraction was performed independently by four reviewers (M.C., S-W.L., Y.S.L., and K.Y.). Any disagreements during the process were resolved by consensus among the reviewers. The following data were extracted from each study: first author and publication year, country, ethnicity, age (years, median age), number of patients, follow-up duration, treatment modality, PBIM threshold values, and survival outcomes, including overall survival (OS), disease-free survival (DFS), and progression-free survival (PFS).

For dynamic PBIM, which incorporated both the pre- and posttreatment levels, the methods of marker assessment were categorized as directional binary and threshold-based binary. A study reporting dynamic PBIM with only the direction (increase or decrease of posttreatment level relative to pretreatment level) of the marker was designated as directional binary. When a threshold was used to assess the dynamic change of posttreatment level compared to pretreatment level, it was identified as threshold-based binary (high vs. low). The posttreatment sampling time was retrieved as described in each study, and then median values in days were estimated for statistical analysis. For example, if the study designated the posttreatment sampling time as “within 4 weeks” of treatment completion, we estimated the median value from a range of 0 to 28 days as 14 days. The Quality in Prognosis Studies (QUIPS) tool was used to assess the risk of bias and select studies that qualified for analysis.

### 2.4 Statistical analysis

Statistical analyses for meta-analysis were primarily performed using the Review Manager Software (version 5.4.1; Cochrane Collaboration, Copenhagen, Denmark), including PRISMA flow diagram and forest plots. Pooled HRs with 95% CIs were calculated to evaluate the association between PBIMs and survival outcomes. A HR > 1 indicated poor survival, whereas a HR < 1 indicated better survival, corresponding with a log HR > 0 and log HR < 0, respectively. Associations between PBIMs and other clinicopathological parameters were assessed using the Mantel–Haenszel method to calculate pooled odds ratios (ORs) with 95% CIs and the combined effective value. An I^2^ value > 50% indicated significant heterogeneity among the studies. Meta-regression analysis was conducted to explore potential sources of heterogeneity, including PBIM variable definitions (categorical vs. continuous), analytic levels (univariate vs. multivariate), and treatment settings (surgery vs. concurrent chemoradiotherapy [CCRT]). Relevant subgroup analyses were performed. The effect of posttreatment sampling time in days was analyzed using the non-linear natural spline meta-regression analysis. The meta-regression analyses were performed using the R software version 4.4.1 (R Core Team 2025) with specific workflow, packages (meta, metafor, splines, etc.), and codes presented in **Supplementary Table S3** (A & B). Publication bias was evaluated using a Python-based workflow implemented in SciPy/Statsmodels within a Jupyter Notebook environment. Detailed scripts and workflows are provided in **Supplementary Table S3** (C).

## 3 Results

### 3.1 Eligible studies

**Figure 1:**
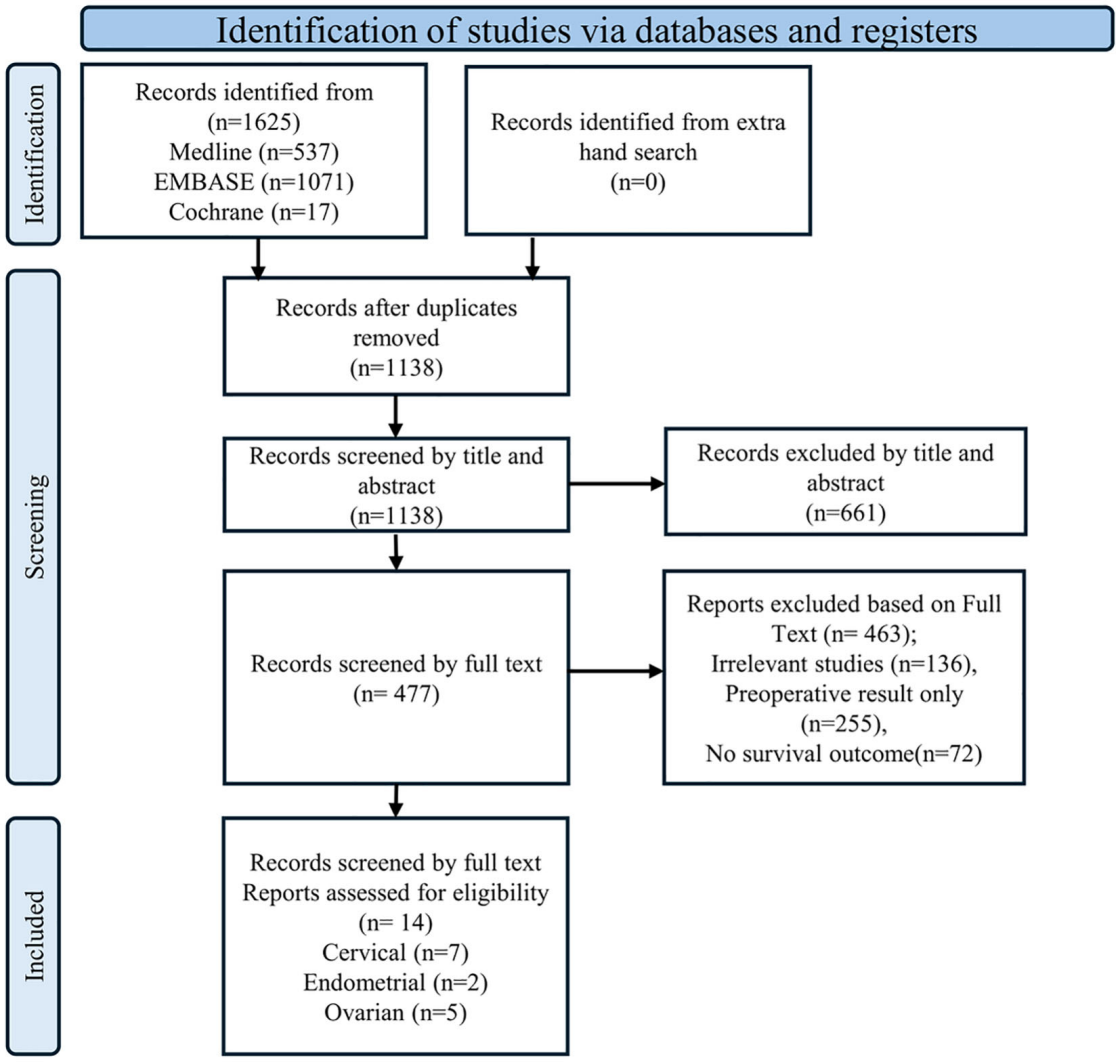
PRISMA flow diagram illustrating the study selection process.

The initial literature search identified 1,625 articles from Medline, EMBASE, and the Cochrane Library (**Figure 1**). After removing 486 duplicate articles, the remaining 1,139 were screened based on reference type criteria. Of these, only 14 articles, comprising seven on cervical cancer (19–24), five on ovarian cancer (26–30) and two on endometrial cancer (31, 32), met the inclusion criteria for this meta-analysis based on data related to prognosis, clinicopathological parameters, and evaluation methods (**Figure 1**). Studies with missing HRs could not be included because, despite contacting the authors, the data could not be retrieved. Most included studies were assessed as having a low risk of bias using the QUIPS tool. The full per-study and per-domain results were summarized in **Supplementary Figure S1**.

### 3.2 Study characteristics

Fourteen studies were included in the final analysis investigating the association between PBIMs and survival rates. These studies were conducted across seven countries and were published between 2017 and 2025 (**Table 1**, **Supplementary Table S4** and **Appendix 1**). A total of 2,373 patients were analyzed, with sample sizes ranging from 51 to 359 and cancer stages across I to IV (**Table 1**, **Supplementary Table S4** and **Appendix 1**). In addition, the PBIM cutoff values used in all included studies were reviewed (**Supplementary Tables S5, S6**).

**Table 1:** Main characteristics of all gynecological cancer studies included in the meta-analysis.

Site	Authors, year	Patients (N)	Stage	Treatment	PBIM	Measurement approach	Dynamic classification^†^	Posttreatment blood test timing (median days)	Endpoint
Cervix	Chao et al., 2023 (19)	359	IA-IIA	Surgery	SIRI	Dynamic	Threshold Binary	Late (42)	OS
	Kim et al., 2020 (20)	107	IB1-IVA	CCRT	NLR	Dynamic	Directional Binary	Early (13)	OS, PFS
	Lee et al., 2020 (21)	125	IIB-IIIB	CCRT	NLR, PLR, MLR	Post, Dynamic	Threshold Binary	Early (14)	OS, DFS
	Trinh et al., 2020 (22)	99	IB-IV	CCRT	NLR, PLR	Post, Dynamic	Continuous Change	Late (90)	OS, PFS
	Du et al., 2023 (23)	164	I-IIA	Surgery	NLR	Post, Dynamic	Directional Binary	Late (30)	OS, PFS
	Chen et al., 2024 (24)	132	IB-IVA	CCRT	NLR, MLR, PLR	Dynamic	Directional Binary	Late (19)	OS, PFS
	Lee et al., 2025 (25)	81	IB-IVA	CCRT	NLR, PLR, MLR, SIRI, SII	Post, Dynamic	Threshold Binary	Early (15)	OS, DFS
Ovary	Kim et al., 2018 (26)	197	IIIB-IVB	Surgery + CTx	NLR	Dynamic	Directional Binary	Early (14)	OS, PFS
	Sanna et al., 2021 (27)	161	IIIC-IVB	Surgery + CTx	NLR	Post	N/A	Early (14)	PFS
	Plaja et al., 2023 (28)	51 (PDS) 80 (IDS)	III-IV	Surgery + CTx	NLR, MLR, PLR	Dynamic	Directional Binary	Late (60)	OS, PFS
	Weng et al., 2023 (29)	307	III-IVA	Surgery + CTx	NLR, PLR	Dynamic	Continuous Change	Late (43)	OS, PFS
	Lazar et al., 2024 (30)	79	IIIA-IV	Surgery + CTx	NLR	Dynamic	Directional Binary	Early (14)	OS, PFS
Endometrium	Ding et al., 2017 (31)	185	I-IV	Surgery +/- CTx/RTx	NLR, PLR	Post	N/A	Early (7)	OS, DFS
	Huang et al., 2021 (32)	246	I-IV	Surgery +/- CTx/RTx	SII	Post	N/A	Early (3)	OS

PDS, primary debulking surgery; IDS, interval debulking surgery; CCRT, concurrent chemoradiotherapy, Ctx, chemotherapy; Rtx, radiotherapy.

^†^Within the dynamic classification, studies were classified into three analytic strategies: continuous change classification, where pre-to-post differences were treated as continuous variables; directional binary classification, where patients were grouped based on whether post-treatment values increased or decreased; and threshold binary classification, where patients were stratified into high vs. low groups using pre/post ratio thresholds.

### 3.3 Elevated peripheral blood inflammatory markers and prognosis in gynecologic cancer

**Figure 2:**
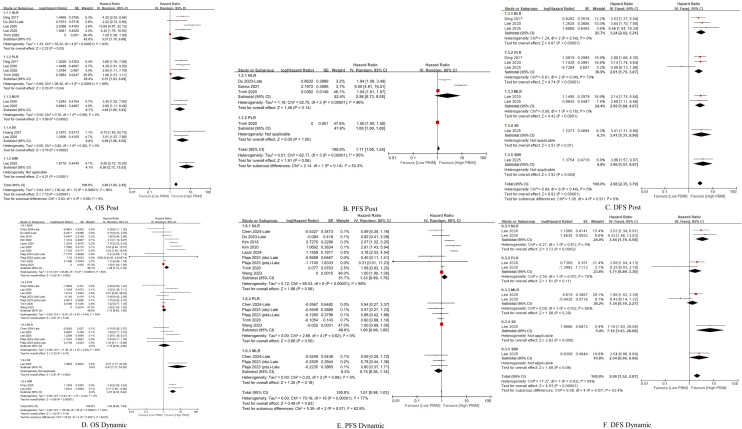
Subgroup hazard ratios for **(A, D)** overall survival (OS), **(B, E)** progression-free survival (PFS) and **(C, F)** disease-free survival (DFS) in patients with gynecologic cancers, according to the type of peripheral blood inflammatory marker (PBIM).

The association between PBIMs and survival endpoints (OS, PFS, and DFS) was examined. Elevated NLR was consistently linked to shorter OS, PFS, and DFS in both the posttreatment and dynamic groups (HRs 1.33 – 3.44). In the posttreatment group, higher PLR (HRs 2.51 and 2.61) and MLR (HRs 3.05 and 2.99) were associated with inferior OS and DFS, respectively. Elevated SII was predictive of worse OS in the posttreatment group (HR: 4.09), whereas a high SIRI was predictive of worse OS in the dynamic group (HR: 3.57) (**Figure 2**). Due to the relative novelty of SII and SIRI, the meta-analysis of these markers could only be performed by including two studies each, thus rendering the interpretation of the results with caution.

### 3.4 Analysis of heterogeneity across included studies

In order to explore the potential sources of heterogeneity among the included studies, the meta-regression analysis was performed for primary tumor site, treatment setting (surgery vs. CCRT), PBIM variable definition (categorical vs. continuous), analytic level (univariate vs. multivariate), and post-treatment sampling time (days). As presented in **Supplementary Table S7**, none of the prementioned factors had a significant effect on meta-analysis except for the post-treatment sampling time. However, acknowledging the baseline heterogeneity among studies and relatively small sample size for meta-analysis, all meta-analyses were performed using the random effects model. The subgroup and sensitivity analyses are shown in **Supplementary Figures S2** (cancer site), S3 (ethnicity), S4 (treatment), S5 (analytic level), and S6 (sensitivity analysis on continuous vs. categorical studies) respectively.

### 3.5 Timing of post-treatment peripheral blood inflammatory marker assessment

In the meta-regression analysis, post-treatment sampling time (in days; R^2^ = 36.76%, *p* = 0.0042) was demonstrated to be the only significant factor (**Supplementary Table S7**). Because all of the 2-, 4-, 6-, and 8-week cutoffs were statistically significant in the sensitivity analysis with meta-regression in a linear function (*p* = 0.0009, *p* = 0.0132, *p* = 0.0013 and *p* = 0.0134, respectively), the non-linear regression analysis was performed to determine the precise cutoff between early vs. late sampling time. As a result, the median of 15 days after treatment completion was identified as the statistically significant cutoff point (*p* = 0.006), shown in **Supplementary Figure S7**. The time point at which the 95% confidence interval (CI) crosses log HR = 0 is approximately 5 weeks after treatment completion. This indicates that the statistical significance of posttreatment sampling is diminished around that time.

**Figure 3:**
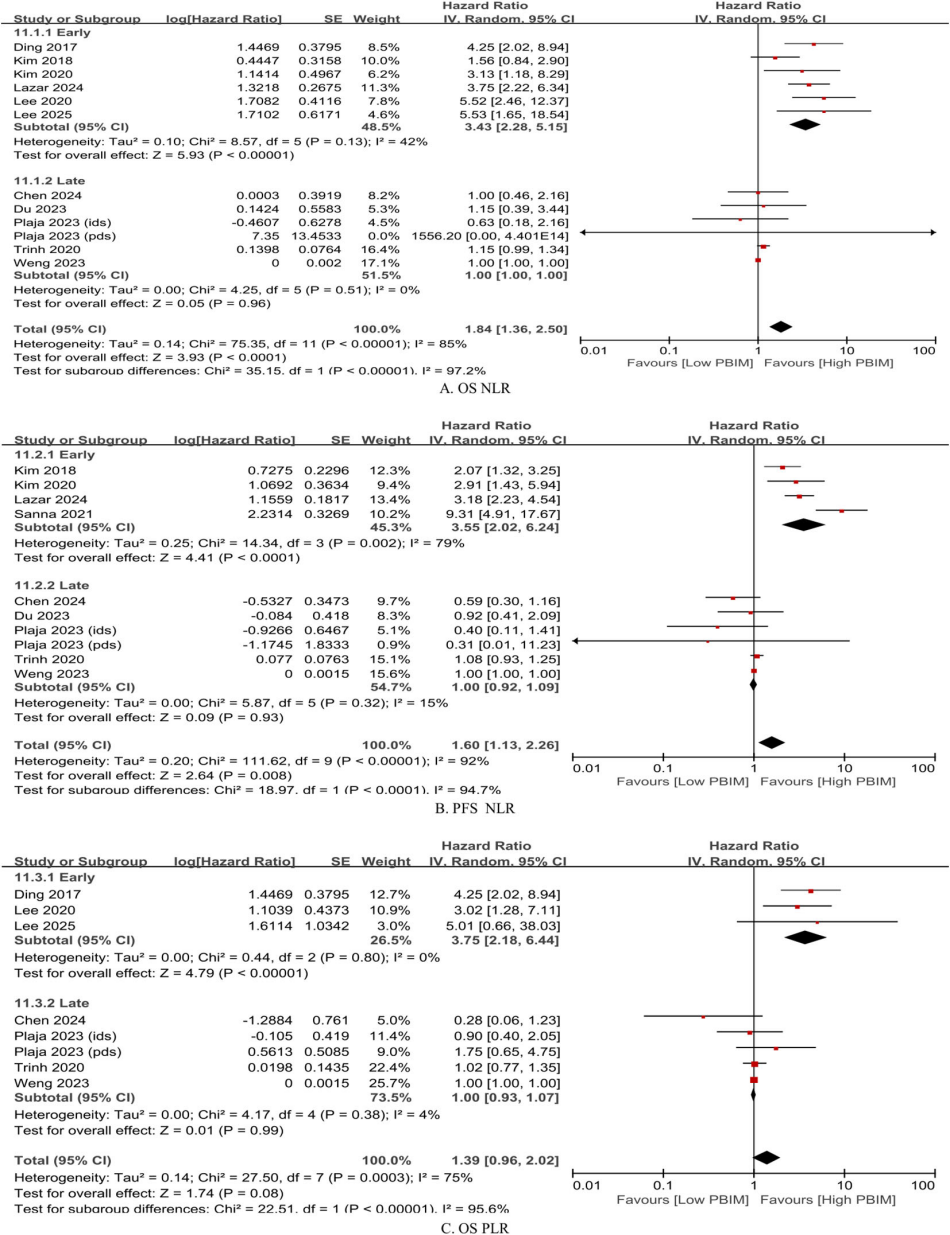
Subgroup hazard ratios by timing of posttreatment blood collection (median ≤ 15 days vs. > 15 days). **(A)** Overall survival (OS) based on neutrophil-to-lymphocyte ratio (NLR); **(B)** progression-free survival (PFS) based on NLR; **(C)** OS based on platelet-to-lymphocyte ratio (PLR).

Based on the cutoff of median 15 days, posttreatment and dynamic PBIM studies were categorized by early vs. late posttreatment sampling time. Measurements obtained within median 15 days after treatment completion were defined as “early phase” (20, 21, 26, 30, 31), whereas those taken more than median 15 days after treatment were defined as “late phase” (23, 24, 28) (**Table 1**). In the early-phase group, elevated PBIMs were significantly associated with poor survival (HR: 3.43 – 3.75; *p* < 0.00001), whereas in the late-phase group, PBIMs were not associated with prognosis (HR: 1.00; *p* = 0.93 – 0.99), as shown in **Figure 3**.

### 3.6 Methods for assessing the dynamic peripheral blood inflammatory markers

**Figure 4:**
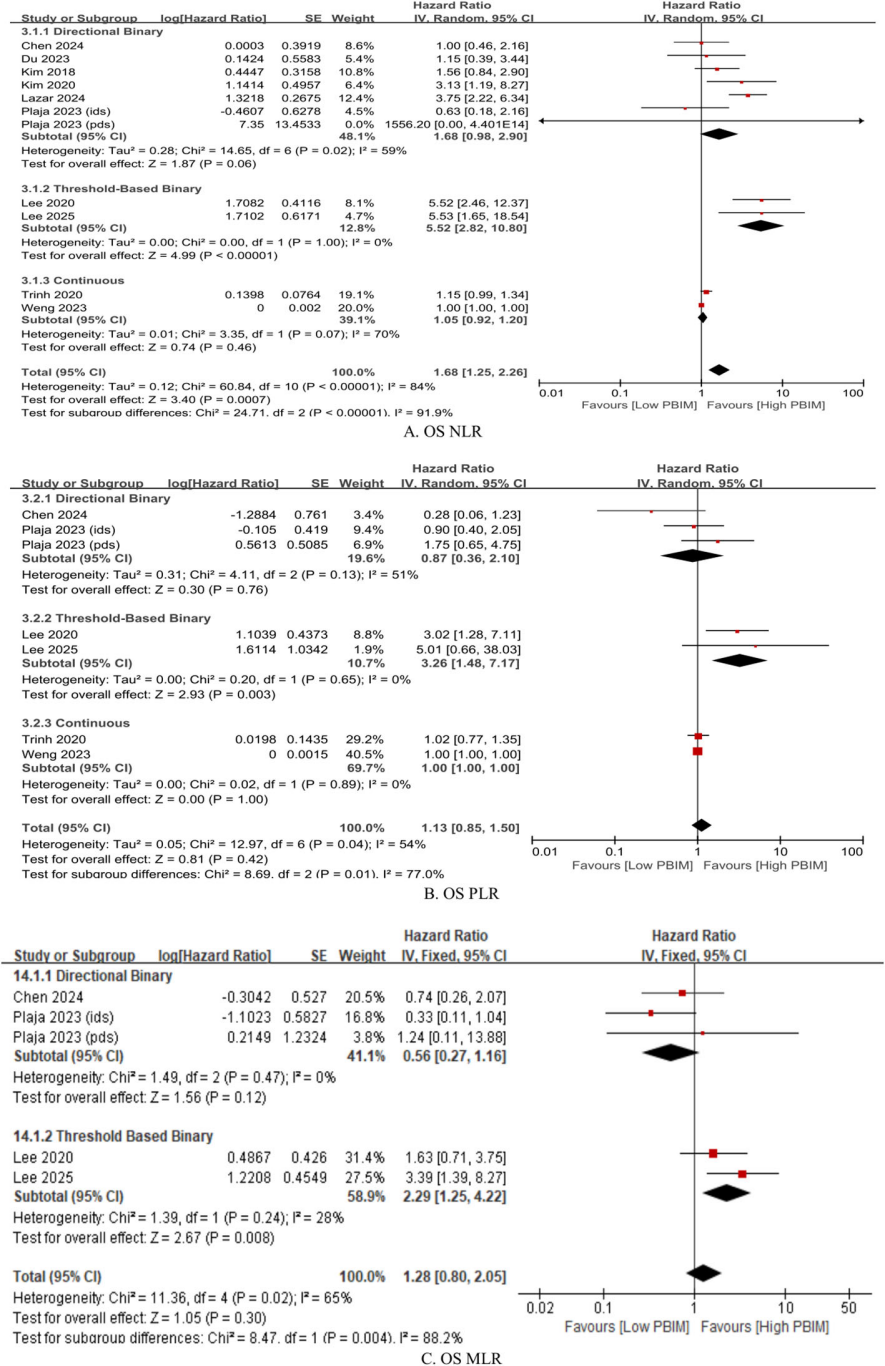
Subgroup hazard ratios for overall survival (OS) in patients with gynecologic cancer according to cutoff method within the dynamic PBIM group. **(A)** Neutrophil-to-lymphocyte ratio (NLR); **(B)** platelet-to-lymphocyte ratio (PLR); **(C)** monocyte-to-lymphocyte ratio (MLR). Threshold-based and directional classifications were compared.

Subgroup analysis of the effects of dynamic PBIMs on OS was performed based on the cutoff methods used. Across different markers, the threshold-based binary method demonstrated the greatest effect with lowest heterogeneity (**Figure 4**; NLR: HR 5.52, *p* < 0.00001, I^2^ = 0%; PLR: HR 3.26, *p* = 0.003, I^2^ = 0%; MLR: HR 2.29, *p* = 0.008, I^2^ = 28%) (20, 21, 23, 24, 26, 28, 30). In case of directional binary method, NLR showed a trend towards significant effect on OS with substantial heterogeneity (**Figure 4**; HR 1.68, *p* = 0.06, I^2^ = 59%). However, the directional binary method failed to show a significant effect on OS with dynamic PLR or MLR (**Figure 4**). The same applied to the continuous method across all dynamic PBIMs (**Figure 4**).

### 3.7 Posttreatment PBIMs and clinicopathological parameters

The main clinicopathological parameters associated with PBIMs across all studies included in the meta-analysis are summarized in **Table 2** and **Supplementary Table S8**. Elevated PBIMs were significantly associated with younger age, type II endometrial carcinoma, and lymphatic invasion (**Table 2**, **Supplementary Table S8** and **Supplementary Figure S8**).

**Table 2:** Summary of meta-analysis evaluating the association between PBIM and clinicopathological parameters in gynecologic cancer.

Parameters	Number of studies	Number of patients	Pooled OR (95% CI)	*P* value	Heterogeneity		
					I^2^ (%)	*P* value	Model
Age (young [ref] vs. old)	3	631	0.66 (0.44-0.99)	0.05	0%	0.41	Fixed
Histology (Endometrium) (Type I [ref] vs. Type II)	2	430	2.52 (1.09-5.84)	0.03	0%	0.93	Fixed
FIGO Stage (Low [ref] vs. High)	3	594	1.61 (0.99-2.61)	0.06	53%	0.12	Fixed
Lymphatic Invasion (Negative [ref] vs. Positive)	3	594	2.23 (1.21-4.09)	0.010	0%	0.37	Fixed
Depth of Invasion (Superficial [ref] vs. Deep)	2	445	0.95 (0.55-1.63)	0.85	35%	0.22	Fixed
Operative Approach (Laparoscopy [ref] vs. Laparotomy)	2	430	0.85 (0.47-1.53)	0.58	0%	0.69	Fixed
Postoperative Chemotherapy (Negative [ref] vs. Positive)	2	430	1.09 (0.61-1.96)	0.77	0%	0.81	Fixed
Postoperative Radiotherapy (Negative [ref] vs. Positive)	2	430	2.28 (0.58-8.86)	0.24	0%	0.68	Fixed
Menopausal Status (Premenopausal [ref] vs. Postmenopausal	2	444	1.28 (0.76-2.16)	0.34	83%	0.01	Fixed
Hypertension (Negative [ref] vs. Positive)	2	430	0.86 (0.46-1.60)	0.63	0%	1.00	Fixed
Diabetes (Negative [ref] vs. Positive)	2	430	0.66 (0.29-1.52)	0.33	65%	0.09	Fixed

OR, odds ratio; CI, confidence interval; ref, reference.

### 3.8 Publication bias

We used a funnel plot, Begg’s test, and Egger’s test to investigate publication bias. The funnel plot was asymmetric, and the trim-and-fill method was used to make the funnel plot symmetric (Appendix). Furthermore, in according to Egger’s linear regression and Begg’s test, no publication bias was found except dynamic PBIM for PFS (*p* = 0.013 and *p* = 0.042) (Appendix 2).

## 4 Discussion

This meta-analysis demonstrated that posttreatment PBIMs and their changes from pre-treatment levels (dynamic PBIMs) are associated with poor prognosis in patients with gynecologic cancers (**Figure 2**). In addition, PBIM values measured within median 15 days after treatment completion served as reliable prognostic indicators, whereas those measured later did not (**Figure 3**, **Supplementary Table S7** and **Supplementary Figure S7**). In case of dynamic PBIMs which incorporated posttreatment levels into pretreatment levels, the threshold-based method (high vs. low) demonstrated a significant association with prognosis compared with the directional method (increase vs. decrease) or the continuous method (**Figure 4**). To the best of our knowledge, this is the first systematic review to comprehensively analyze the prognostic role of posttreatment PBIMs and their dynamic change relative to pretreatment levels.

Because the current literature on the role of PBIMs in gynecologic disease (35, 36) and cancers (37, 38) is devoid of information about sampling times, and even if it does, since most studies involve pretreatment levels we found it warranted to conduct a systematic review on the role of posttreatment PBIMs in gynecologic cancers. As if to reflect the reality, we yielded a low inclusion rate of 0.68% after the literary search. In our previous studies, we experienced cases in which studies were excluded during the search process when keywords related to outcomes were used, even if they contained survival data. This usually occurred in studies that were primarily conducted in cell lines or animal models and validated in actual patients, but lacked prognosis data in the title or abstract. To overcome this problem, we expanded the body of literature beyond PICO keywords during the search process and then proceeded according to PICO keywords during the actual paper selection process. As we suspected, there were no studies that reported CSS, and only three reported DFS (21, 31). This may be because PBIMs are associated with not only cancer recurrence (39), but also the patient’s overall condition (40) and disease progression (41). Consequently, the meta-analysis came to focus on integrated outcomes, such as OS and PFS. In addition to the rigorous methodology employed to identify all relevant studies on PBIMs in gynecologic cancers, the limited number of studies evaluating relatively newer markers such as SIRI (n = 2) (19) and SII (n = 2) (32) also contributed to the low inclusion rate.

The potential value of PBIMs in cancer prognosis has long been recognized. Biologically, lymphocytes play a central role in anti-tumor immunity (42). Upon activation by antigen-presenting cells, CD8^+^ T cells differentiate into cytotoxic T lymphocytes (CTLs), which mediate tumor cell lysis via perforin- and granzyme-dependent exocytosis (43, 44). CD4^+^ helper T cells augment this response by producing interleukin-2, tumor necrosis factor-α, and interferon-γ, which promote CTL function, enhance macrophage and NK cell activity, and increase tumor antigen presentation (45). In contrast, neutrophils (42, 46), monocytes (which differentiate into macrophages in tissues) (42, 47), and platelets exhibit pro-tumorigenic effects (48). Based on this biology, ratios using neutrophils, monocytes, and/or platelets as the numerator and lymphocytes as the denominator have been reported as a convenient means for predicting poor survival across various cancers (9–13). Most of the studies on the pretreatment values found association with the tumor microenvironment and reported that they could serve as effective prognostic indicators (14–18).

The individual studies on posttreatment PBIMs were again shown to have association with poor prognosis in this meta-analysis of gynecologic malignancies (**Figure 2**). While pretreatment PBIMs can be considered to reflect the intrinsic tumor microenvironment, posttreatment PBIMs may provide insights into the therapy-induced immunosuppression and the kinetics of immune reconstitution (33, 34). It is well known that prolonged or severe immunosuppression caused by intense cancer treatment can compromise immune surveillance. Based on pro-tumor immune cells such as neutrophils (34, 49), monocytes (34, 50), and platelets (51) recover more rapidly after treatment, whereas anti-tumor lymphocytes have relatively delayed recovery (34, 52), posttreatment PBIMs incorporate these features and may have the potential to serve as a prognostic indicator.

To address the heterogeneity of the included studies, the meta-regression analysis was performed to identify potential sources of heterogeneity, including primary tumor site, treatment setting, PBIM variable definition, analytic level, and posttreatment sampling time. As a result, posttreatment sampling time was demonstrated as the only significant factor, and the remaining moderators were not significant effect on the meta-analysis (**Supplementary Table S7**).

Additionally, the sampling time of posttreatment levels was assessed. First, after estimation into days, the posttreatment sampling time was identified as a significant factor through meta-regression analysis (**Supplementary Table S7**). Next, the precise cutoff between early and late sampling was determined using a non-linear meta-regression analysis. As a result, posttreatment PBIMs measured within 30 days (median 15 days) after treatment completion, demonstrated a significant association with poor prognosis (**Figure 3** and **Supplementary Figure S7**). This result should be interpreted with caution regarding survivorship bias. However, the posttreatment sampling window (day 0–90) was relatively short, likely limiting its impact.

This finding may be partly attributed to the relatively rapid recovery of neutrophils, monocytes, and platelets (34, 49–51), as opposed to the delayed recovery of all major circulating lymphocyte subsets (34, 52). Consequently, early-phase PBIMs are more likely to reflect the imbalance between rapidly recovering pro-tumorigenic cells and slowly recovering antitumor lymphocytes, which heightens their potential as a surrogate marker of immune recovery capacity (34). In the late-phase, pro-tumor cell counts tend to stabilize whereas lymphocyte numbers gradually increase over time (34). As a result, PBIMs may be more susceptible to variability depending on when they are sampled and when external immune-activating events occur. This could compromise their prognostic relevance.

Several approaches for evaluating posttreatment PBIMs as prognostic markers have been reported in the literature. First, Trinh et al. (22) and Weng et al. (29) directly analyzed PBIMs as continuous variables in relation to survival, whereas all other studies used binary PBIM values for the analysis. Second, some of the studies adopted a dynamic approach, comparing both pre- and posttreatment PBIM values, rather than using posttreatment levels alone. This method allows adjustment for each patient’s baseline inflammatory status and has a potential to better reflect individual immune changes over time. Furthermore, within the dynamic approach, the classification could be made into two approaches: the directional approach, which categorizes patients based on whether PBIMs increase or decrease after treatment, and the threshold-based approach, which uses the difference or ratio between pre- and posttreatment values and applies a specific cut-off value (**Table 1**). The threshold-based method showed more homogeneous results across studies (**Figure 4**).

Another significant factor related to posttreatment PBIMs was younger age. This observation may reflect age-related immunosenescence, which results in lower PBIM values in older patients (53). High PBIMs were also associated with poor prognostic clinicopathological factors, such as lymphatic invasion and type II histology in endometrial cancer, indicating that posttreatment PBIM may increase in more aggressive tumors. Therefore, posttreatment PBIMs have the potential to reflect both the patient’s immune response and the intrinsic aggressiveness of the tumors. However, current evidence is limited to a small number of studies (23, 31, 32), and larger studies are needed to validate these findings.

This study has several limitations: (i) We were unable to collect information on various confounders that may influence PBIMs, including perioperative inflammation, infections, corticosteroid use, tumor burden, circadian variations in leukocyte and platelet counts, and standardization of blood draw timing. Prospective studies that adequately control for each of these factors are warranted. (ii) This analysis pooled results from primary studies that used various non-standardized cutoff values. Therefore, a clinically optimized and validated threshold could not be determined. (iii) The incremental prognostic value of PBIMs compared to existing prognostic models was not evaluated (e.g., via C-index, Net Reclassification Improvement [NRI], or Integrated Discrimination Improvement [IDI]). (iv) Studies not published in the English language were excluded due to the difficulty in obtaining precise data, which may have introduced selection bias. (v) For studies that did not report HRs with 95% CIs, the data were extracted using an indirect method prior to pooled HR calculation, which may have compromised the accuracy of the data. (vi) There is a limited number of studies on recently investigated markers, such as SIRI and SII, highlighting the caution for interpretation of the results and the need for further research. (vii) The results of posttreatment sampling time should be interpreted with caution, as survivorship bias cannot be ruled out. We must emphasize that our conclusion should be limited to positioning PBIMs as a potentially useful marker that requires further validation rather than as a clinically established prognostic tool; nevertheless, the meta-analysis indicates the potential value of PBIM in prognostication and in understanding its clinicopathological significance in gynecological cancers.

## 5 Conclusion

Posttreatment PBIMs and their dynamic changes from pretreatment levels showed significant association with poor prognosis in patients with gynecologic cancers. The potential of posttreatment PBIMs as prognostic biomarker of gynecologic cancers has been demonstrated. While it remains a hypothesis, the underlying mechanism may involve immune suppression and subsequent recovery after cancer treatment. Notably, posttreatment PBIMs measured within 30 days after therapy and those assessed using threshold-based classification demonstrated stronger prognostic value, underscoring the need for standardized timing and cut-off values in future clinical applications.

## Data Availability

The original contributions presented in the study are included in the article/**Supplementary Material**. Further inquiries can be directed to the corresponding author.

## Supplementary material

Supplementary Figure S1 Risk of bias assessment using the Quality in Prognosis Studies (QUIPS) tool.

Supplementary Figure S2 Subgroup hazard ratios **(A)** OS and **(B)** PFS in gynecological cancer patients according to organ type.

Supplementary Figure S3 Subgroup hazard ratios **(A)** OS and **(B)** PFS in gynecological cancer patients according to ethnicity.

Supplementary Figure S4 Subgroup hazard ratios in gynecological cancer patients according to the treatment options.

Supplementary Figure S5 Subgroup hazard ratios **(A, B)** OS and **(C)** PFS in gynecological cancer patients according to the univariate vs multivariate analysis.

Supplementary Figure S6 Sensitivity analyses comparing continuous-variable vs categorical-variable studies for posttreatment OS **(A, B)**, posttreatment PFS **(C, D)**, dynamic OS **(E, F)**, and dynamic PFS **(G, H)**. Z values were similar when continuous studies were included **(A, C, E, G)** versus excluded **(B, D, F, H)**.

Supplementary Figure S7 Non-linear spline meta-regression defines early versus late windows. Significant impact was identified at or before median 15 days of sampling time.

Supplementary Figure S8 Subgroup odds ratio analyzing the PBIM and pathological parameters in gynecological cancer patients **(A)** age, **(B)** histology and **(C)** lymphatic invasion.
