# Supplementary figures and images for: Can posttreatment blood inflammatory markers predict poor survival in gynecologic cancer?: a systematic review and meta-analysis
Source: Front Immunol. 2025 Oct 21;16:1676838. doi: 10.3389/fimmu.2025.1676838 (PMC12583213; doi:10.3389/fimmu.2025.1676838)

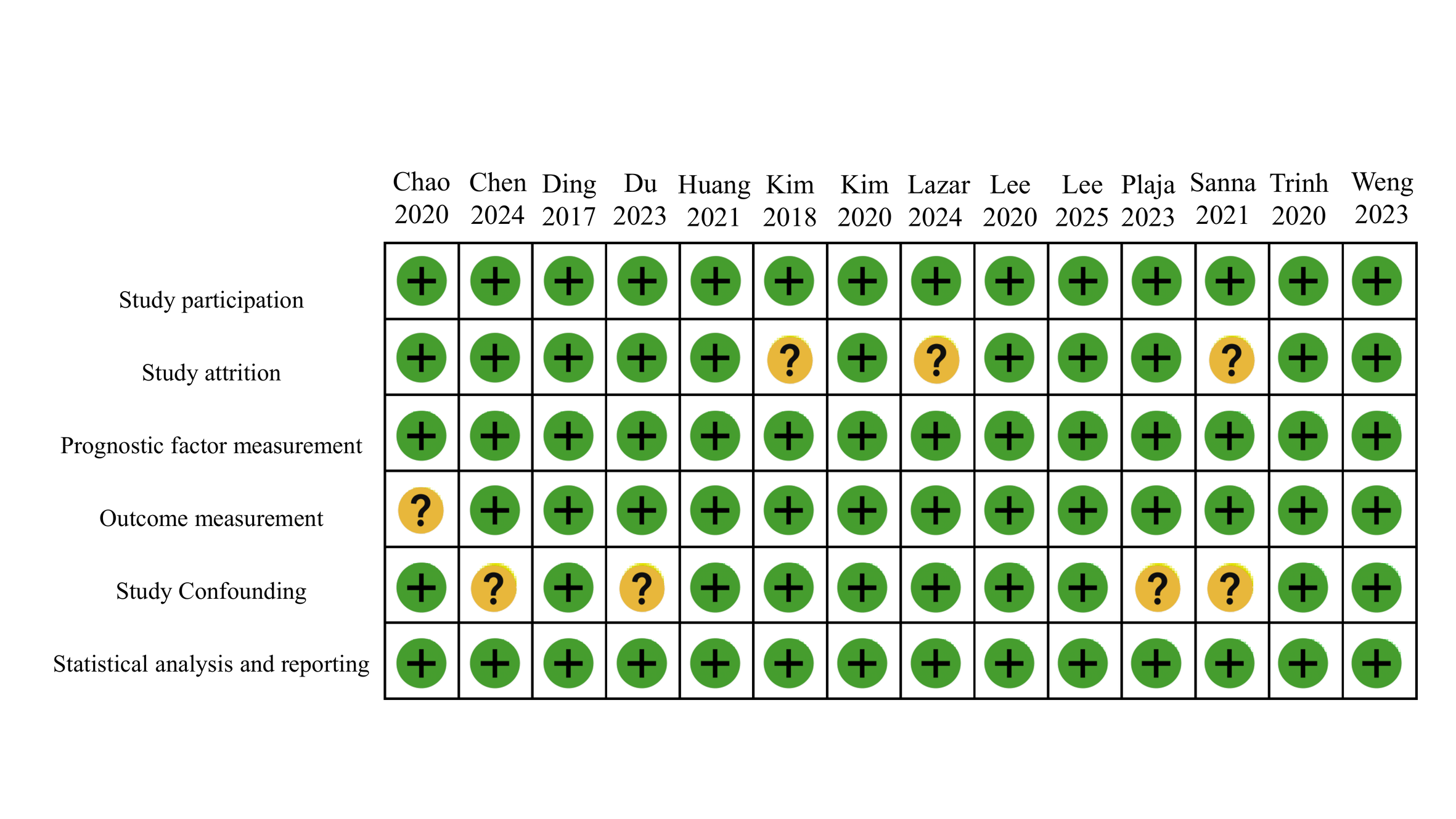

Supplement: Supplementary Figure S1 — Risk of bias assessment using the Quality in Prognosis Studies (QUIPS) tool. [file Image1.tiff]

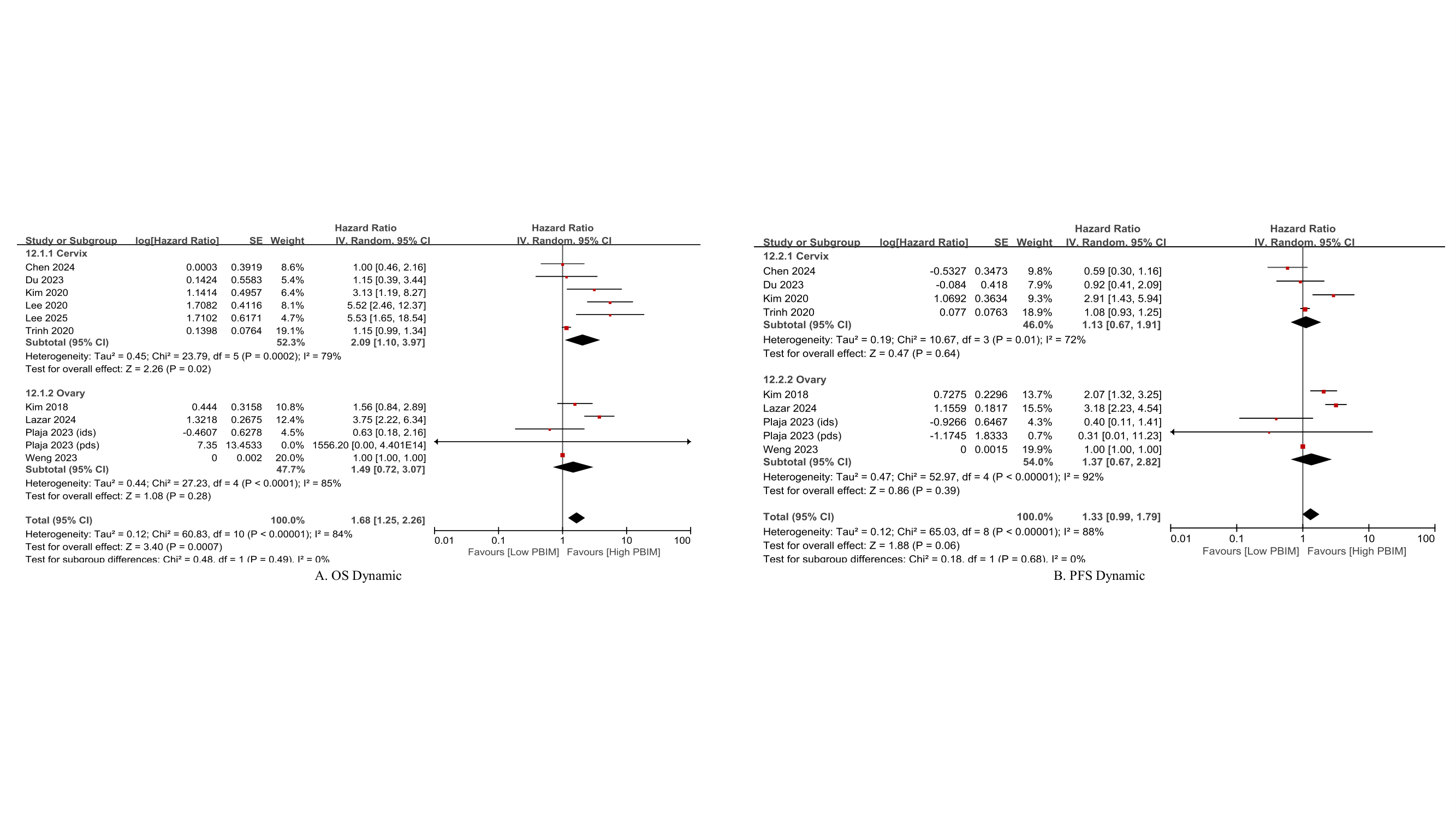

Supplement: Supplementary Figure S2 — Subgroup hazard ratios (A) OS and (B) PFS in gynecological cancer patients according to organ type. [file Image2.tiff]

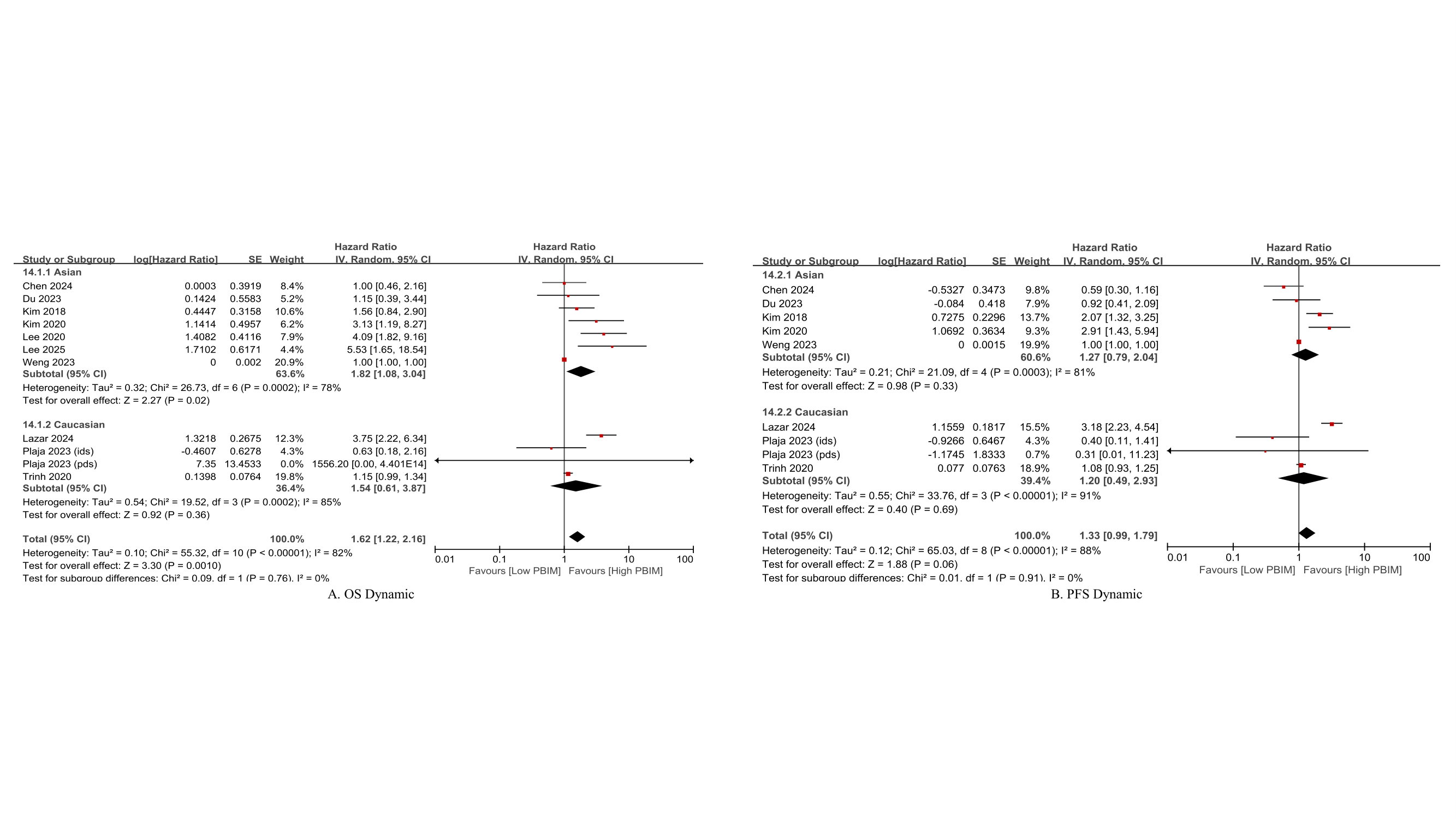

Supplement: Supplementary Figure S3 — Subgroup hazard ratios (A) OS and (B) PFS in gynecological cancer patients according to ethnicity. [file Image3.tiff]

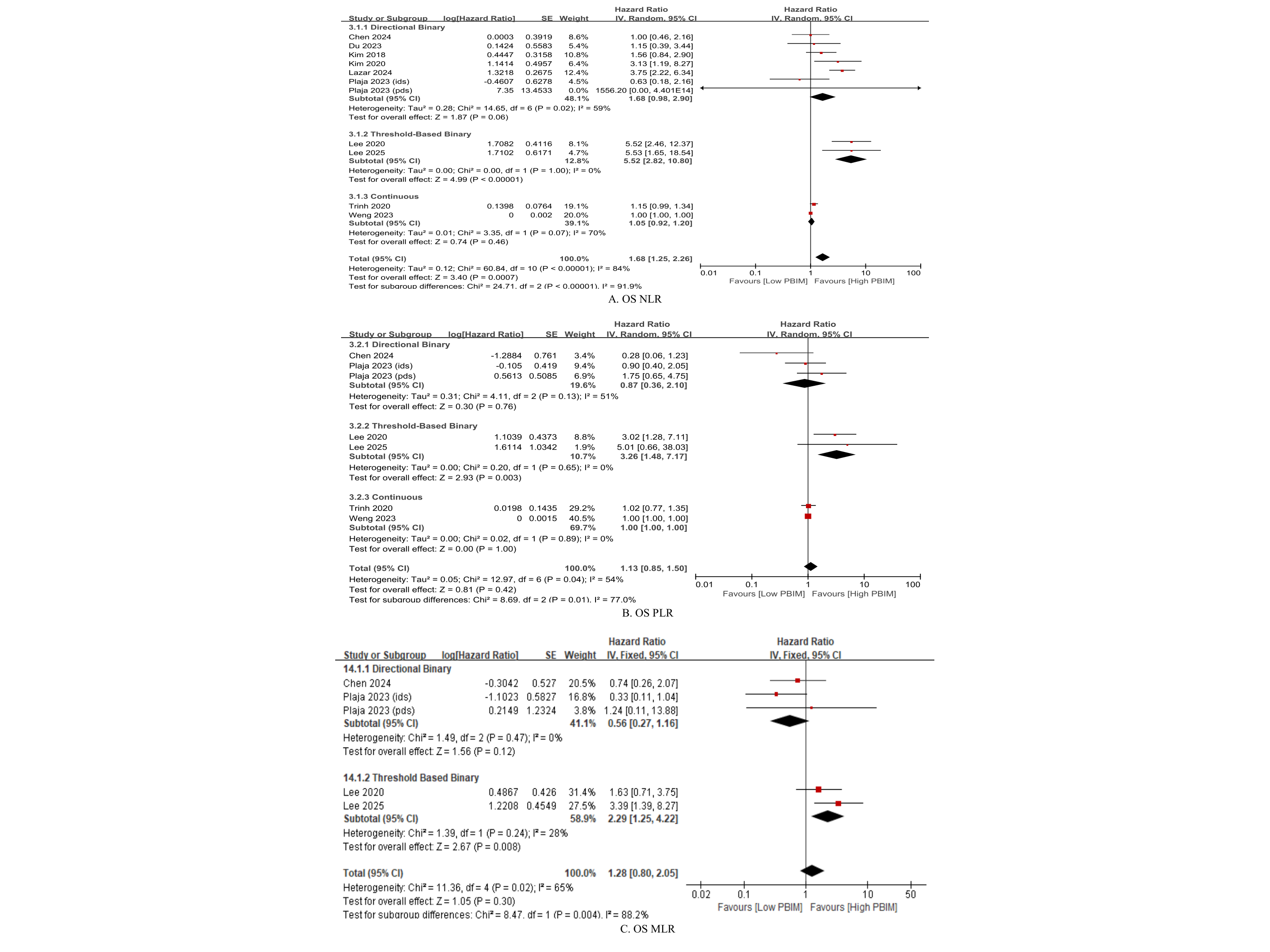

Supplement: Supplementary Figure S4 — Subgroup hazard ratios in gynecological cancer patients according to the treatment options. [file Image4.tiff]

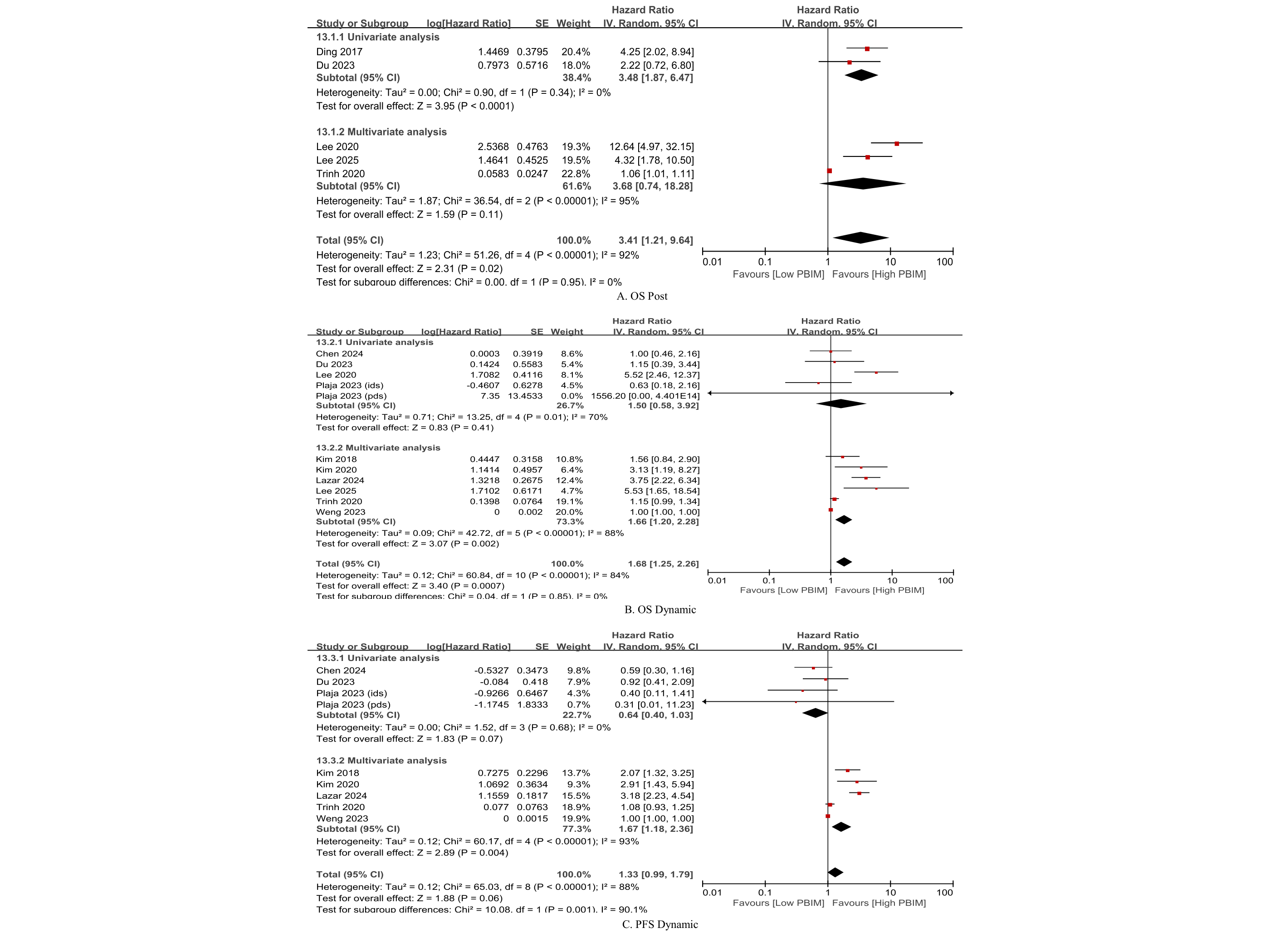

Supplement: Supplementary Figure S5 — Subgroup hazard ratios (A, B) OS and (C) PFS in gynecological cancer patients according to the univariate vs multivariate analysis. [file Image5.tiff]

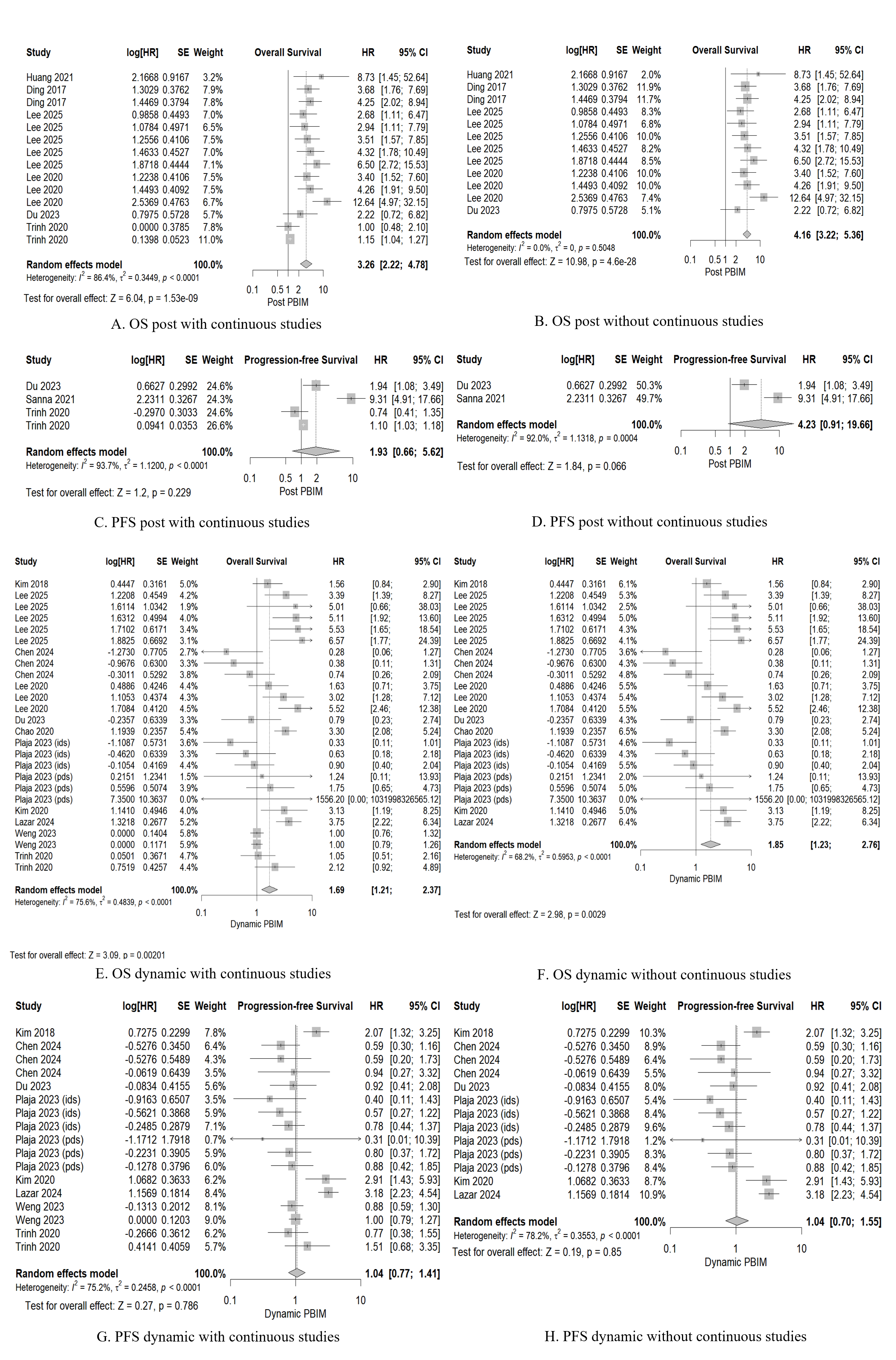

Supplement: Supplementary Figure S6 — Sensitivity analyses comparing continuous-variable vs categorical-variable studies for posttreatment OS (A, B), posttreatment PFS (C, D), dynamic OS (E, F), and dynamic PFS (G, H). Z values were similar when continuous studies were included (A, C, E, G) versus excluded (B, D, F, H). [file Image6.tif]

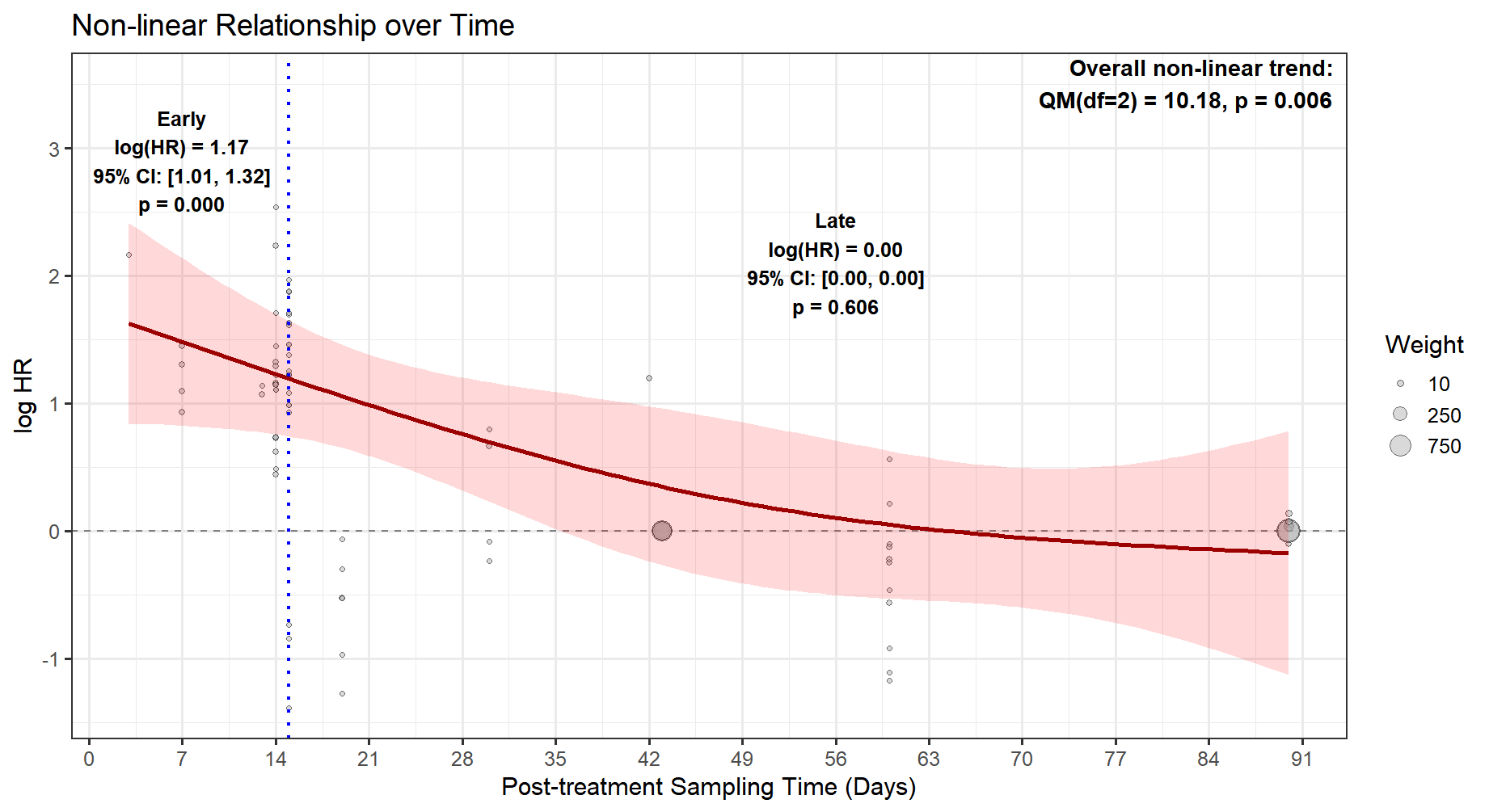

Supplement: Supplementary Figure S7 — Non-linear spline meta-regression defines early versus late windows. Significant impact was identified at or before median 15 days of sampling time. [file Image7.tiff]

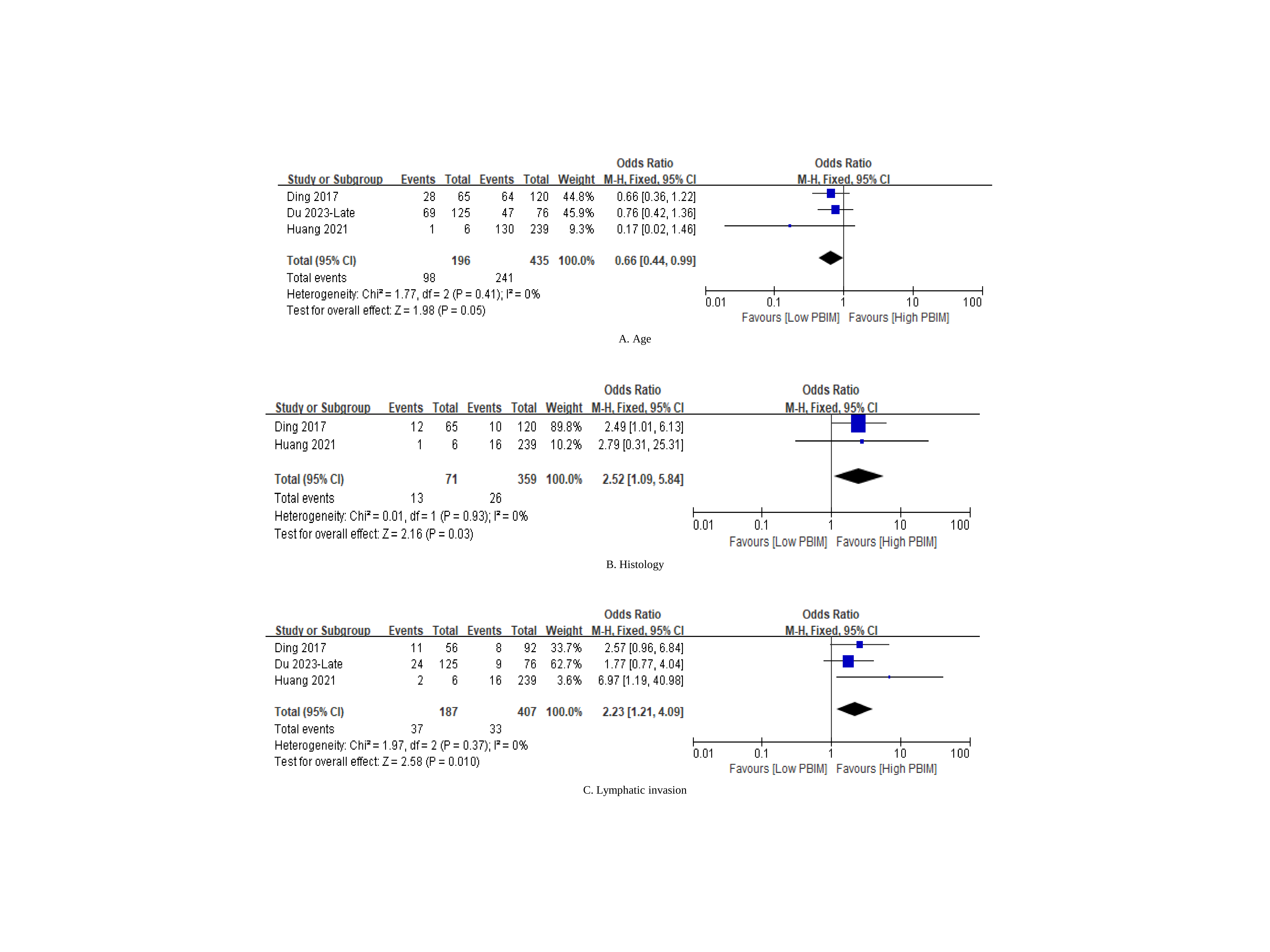

Supplement: Supplementary Figure S8 — Subgroup odds ratio analyzing the PBIM and pathological parameters in gynecological cancer patients (A) age, (B) histology and (C) lymphatic invasion. [file Image8.tiff]

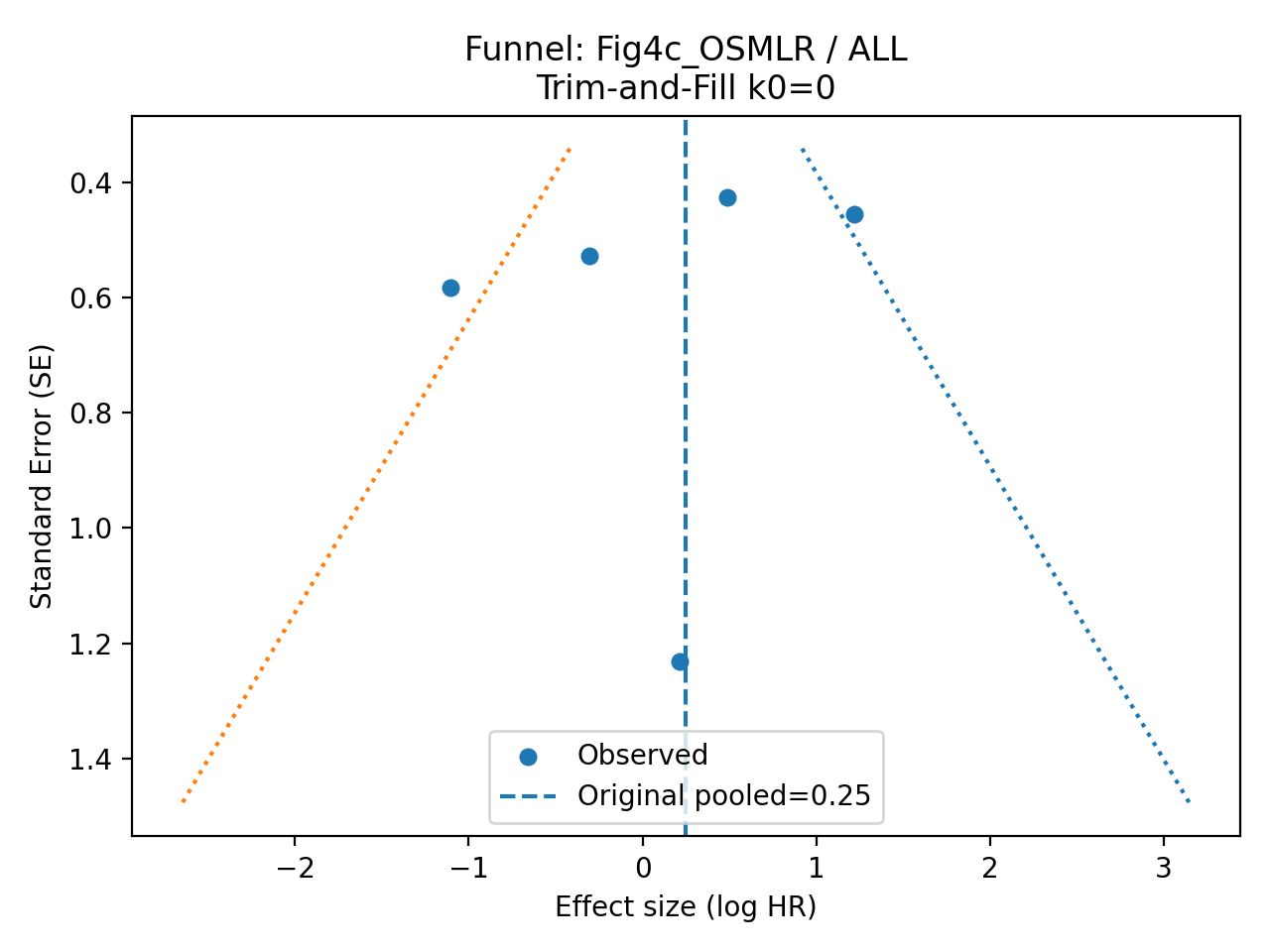

Supplement: Supplementary file 10 [file DataSheet2.zip › Fig4c_OSMLR_TrimAndFill_ALL.png]

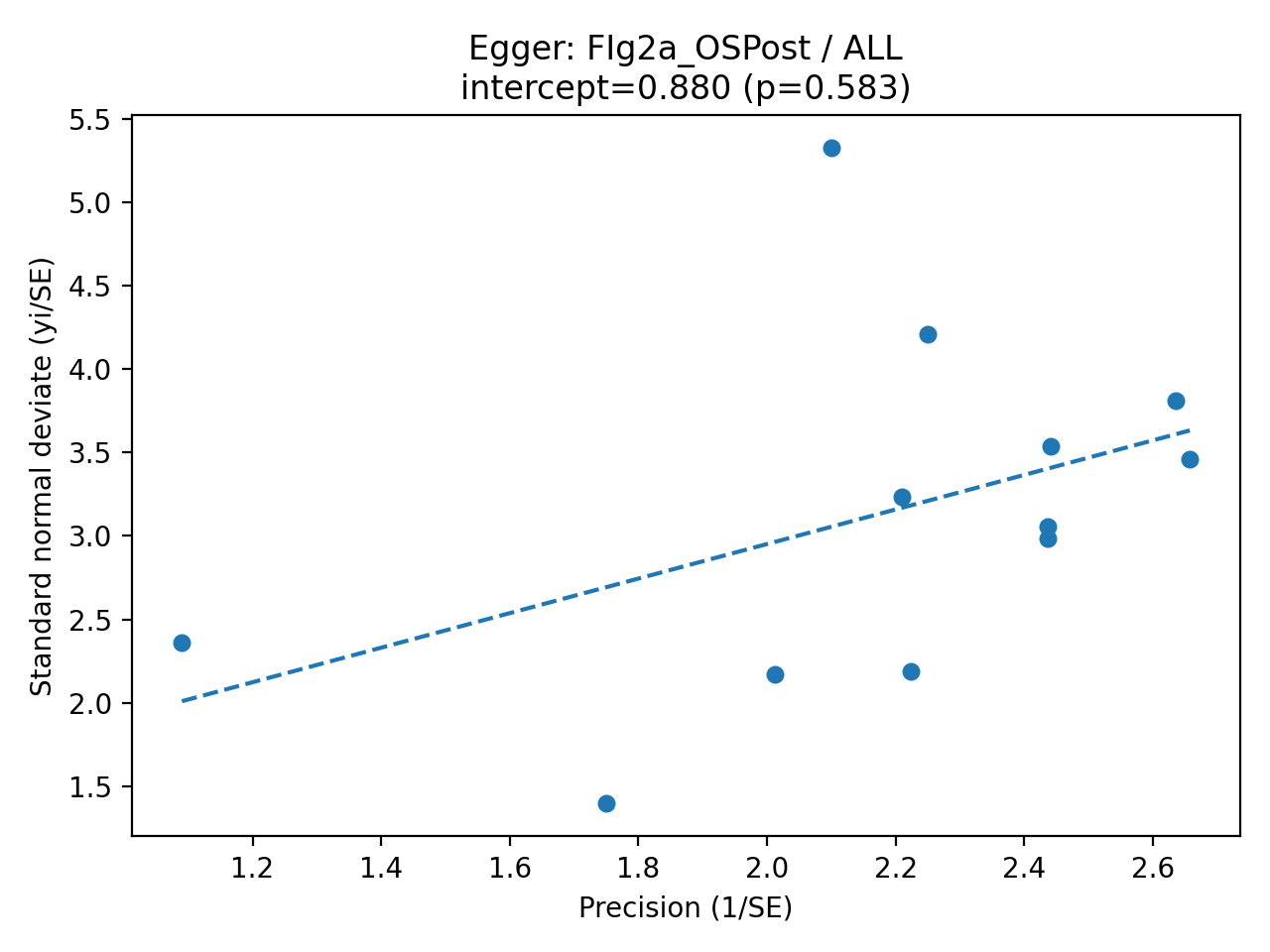

Supplement: Supplementary file 10 [file DataSheet2.zip › FIg2a_OSPost_egger_ALL.png]

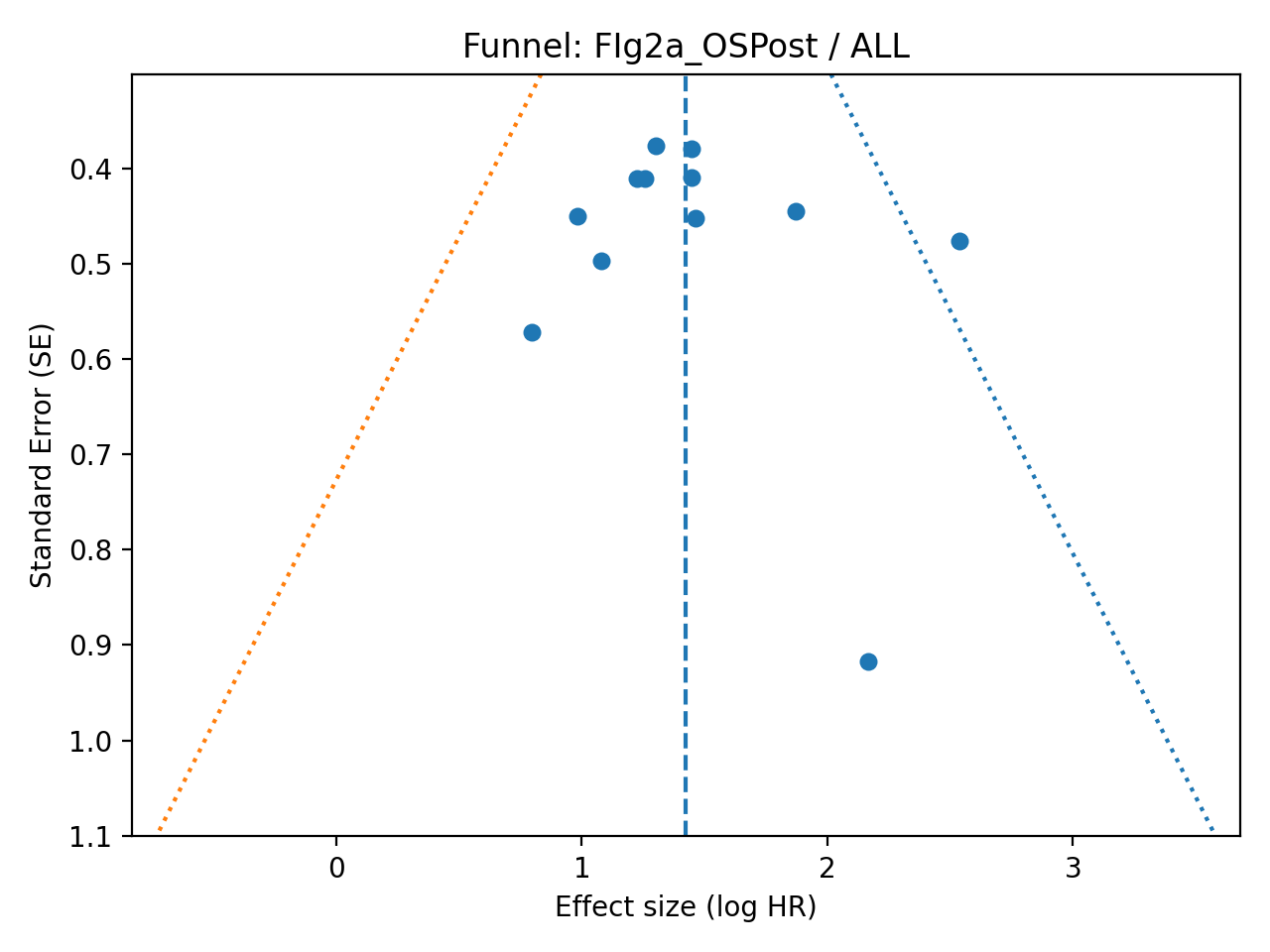

Supplement: Supplementary file 10 [file DataSheet2.zip › FIg2a_OSPost_funnel_ALL.png]

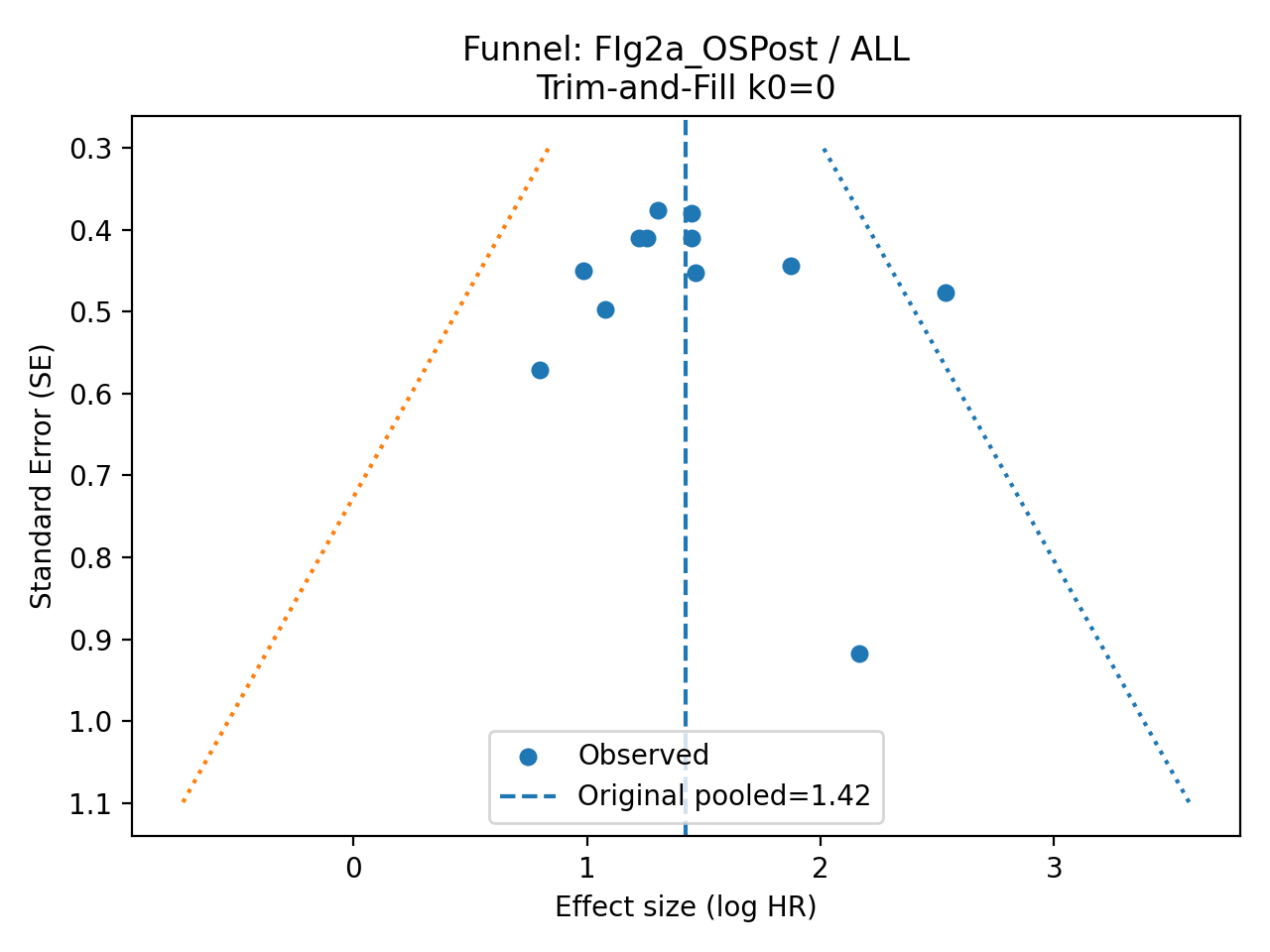

Supplement: Supplementary file 10 [file DataSheet2.zip › FIg2a_OSPost_TrimAndFill_ALL.png]

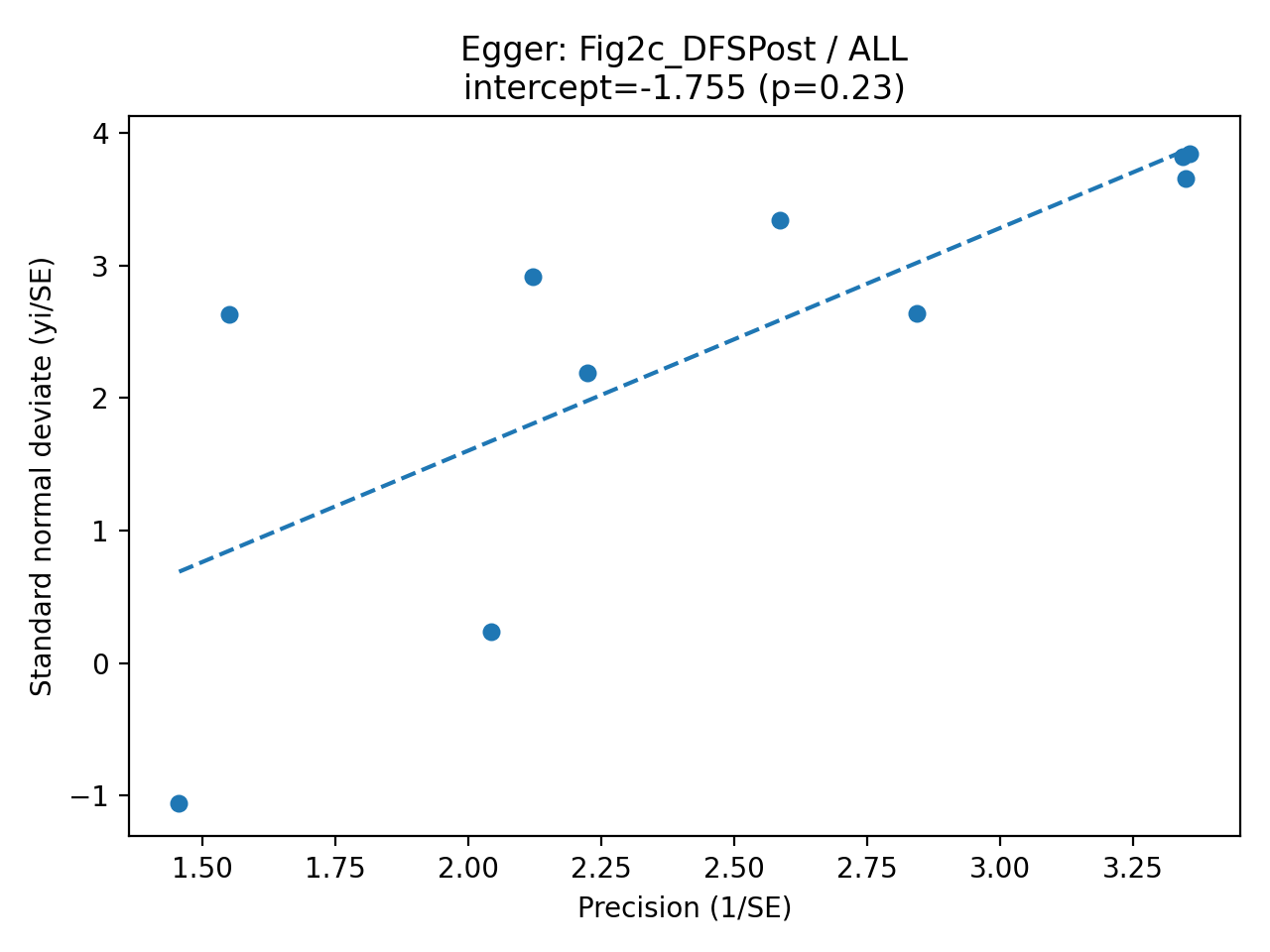

Supplement: Supplementary file 10 [file DataSheet2.zip › Fig2c_DFSPost_egger_ALL.png]

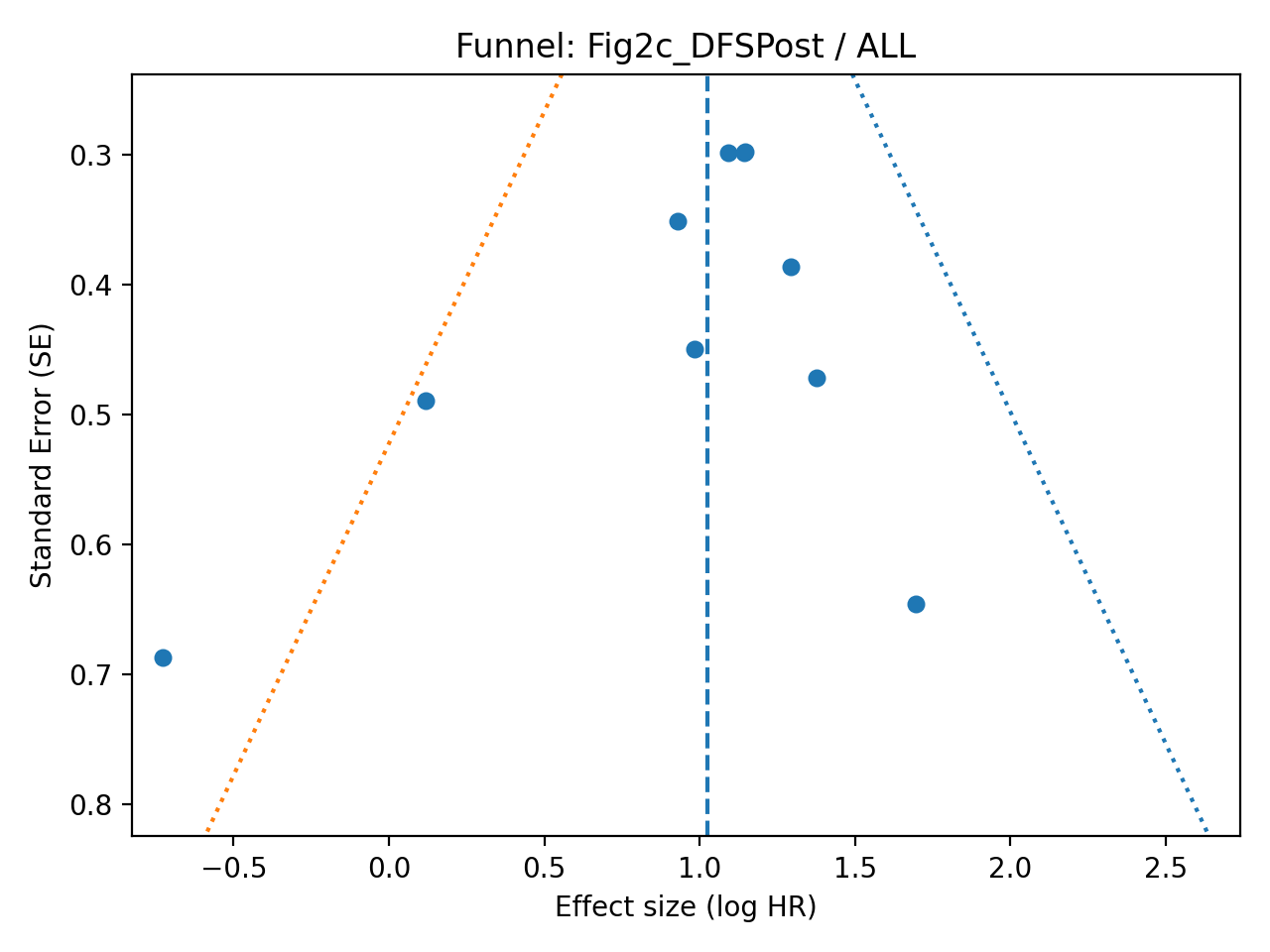

Supplement: Supplementary file 10 [file DataSheet2.zip › Fig2c_DFSPost_funnel_ALL.png]

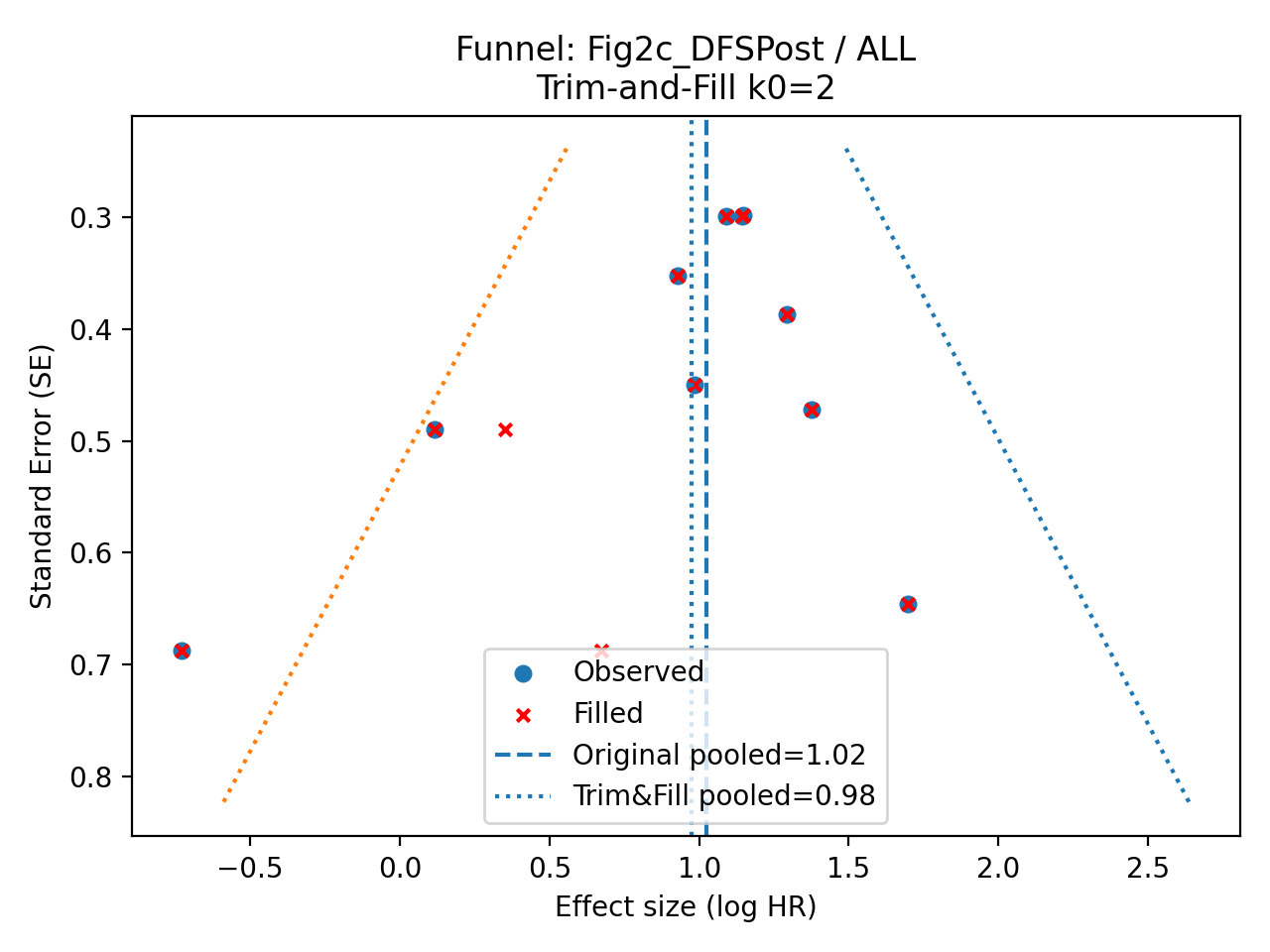

Supplement: Supplementary file 10 [file DataSheet2.zip › Fig2c_DFSPost_TrimAndFill_ALL.png]

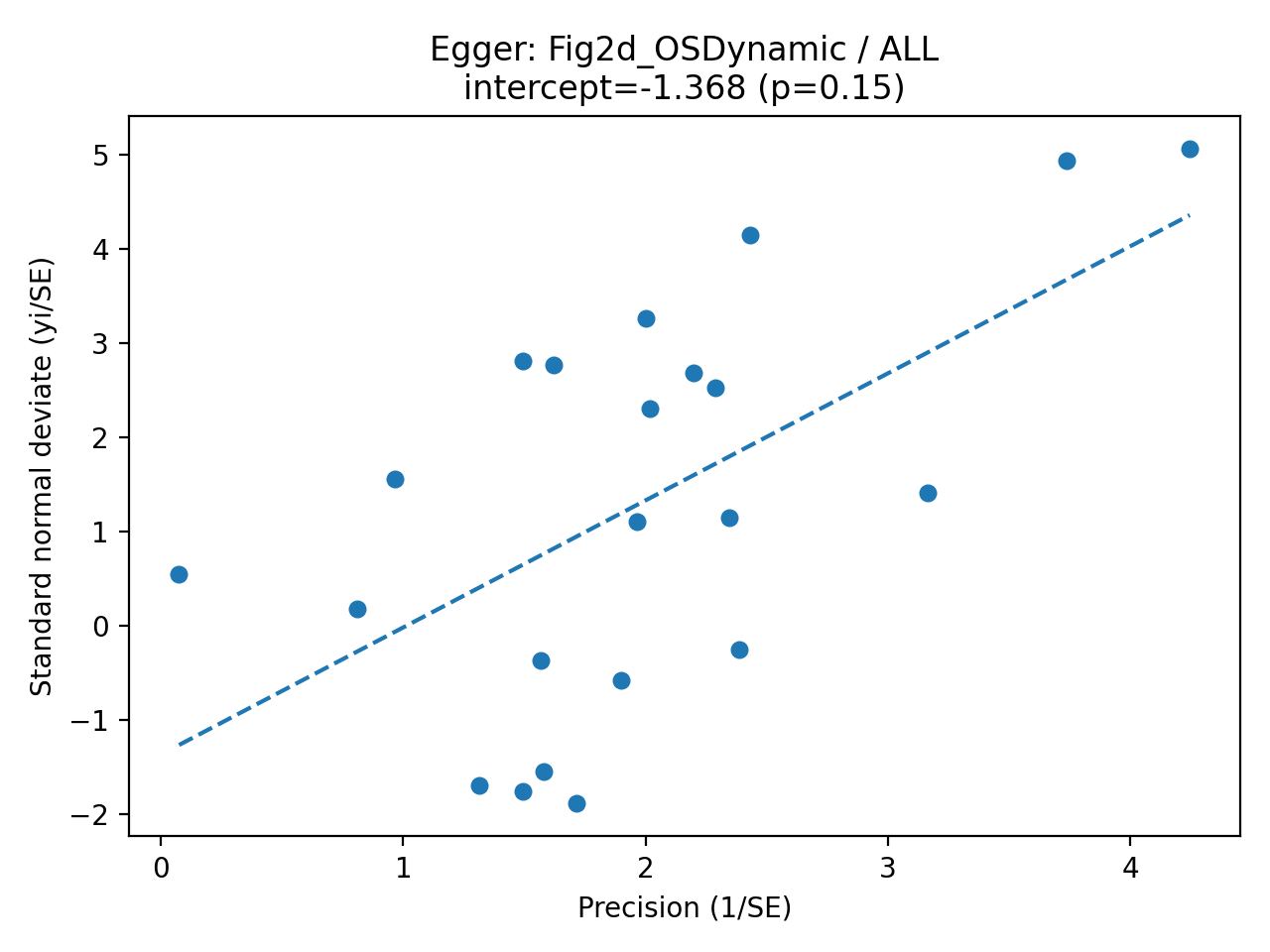

Supplement: Supplementary file 10 [file DataSheet2.zip › Fig2d_OSDynamic_egger_ALL.png]

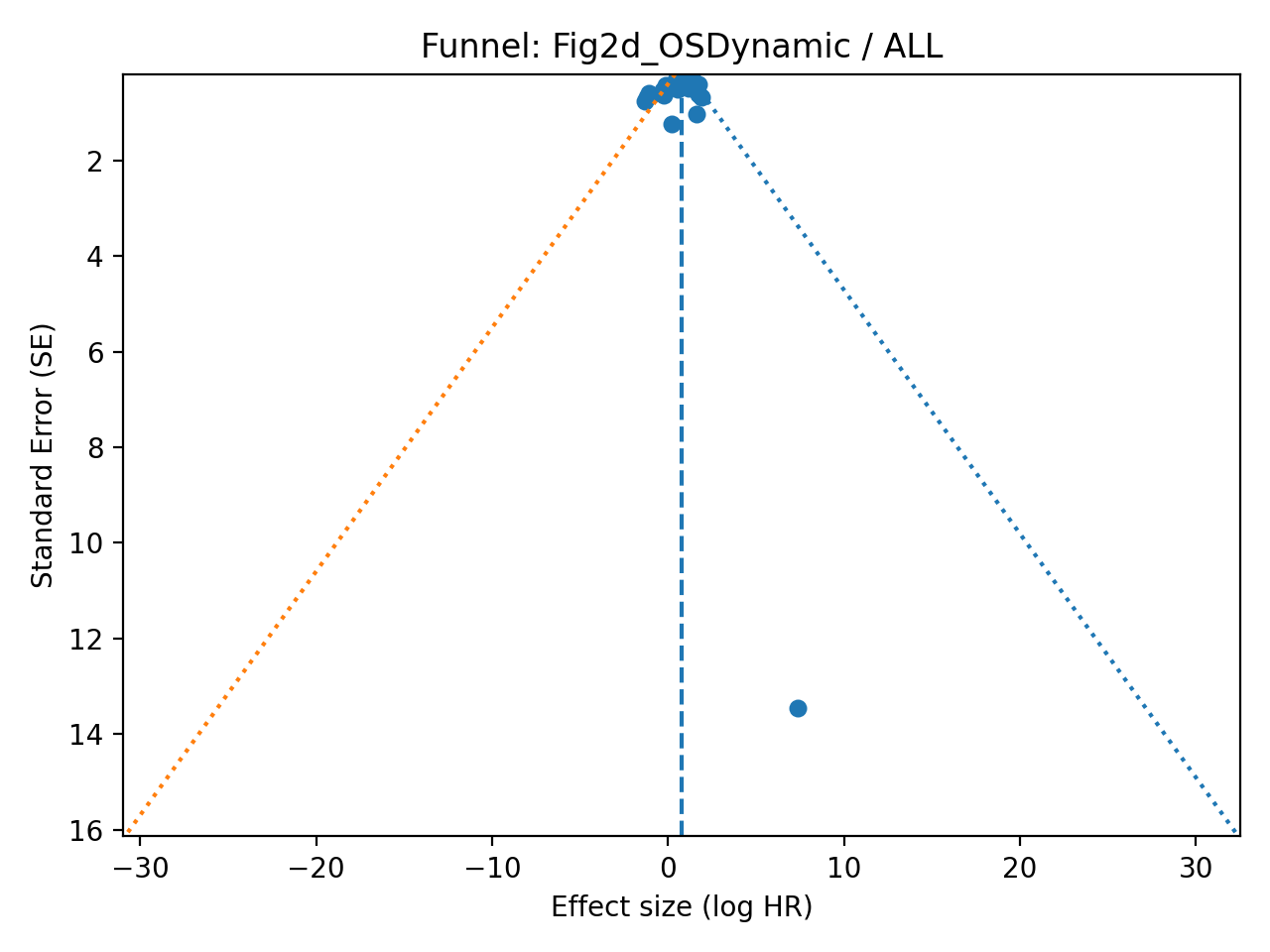

Supplement: Supplementary file 10 [file DataSheet2.zip › Fig2d_OSDynamic_funnel_ALL.png]

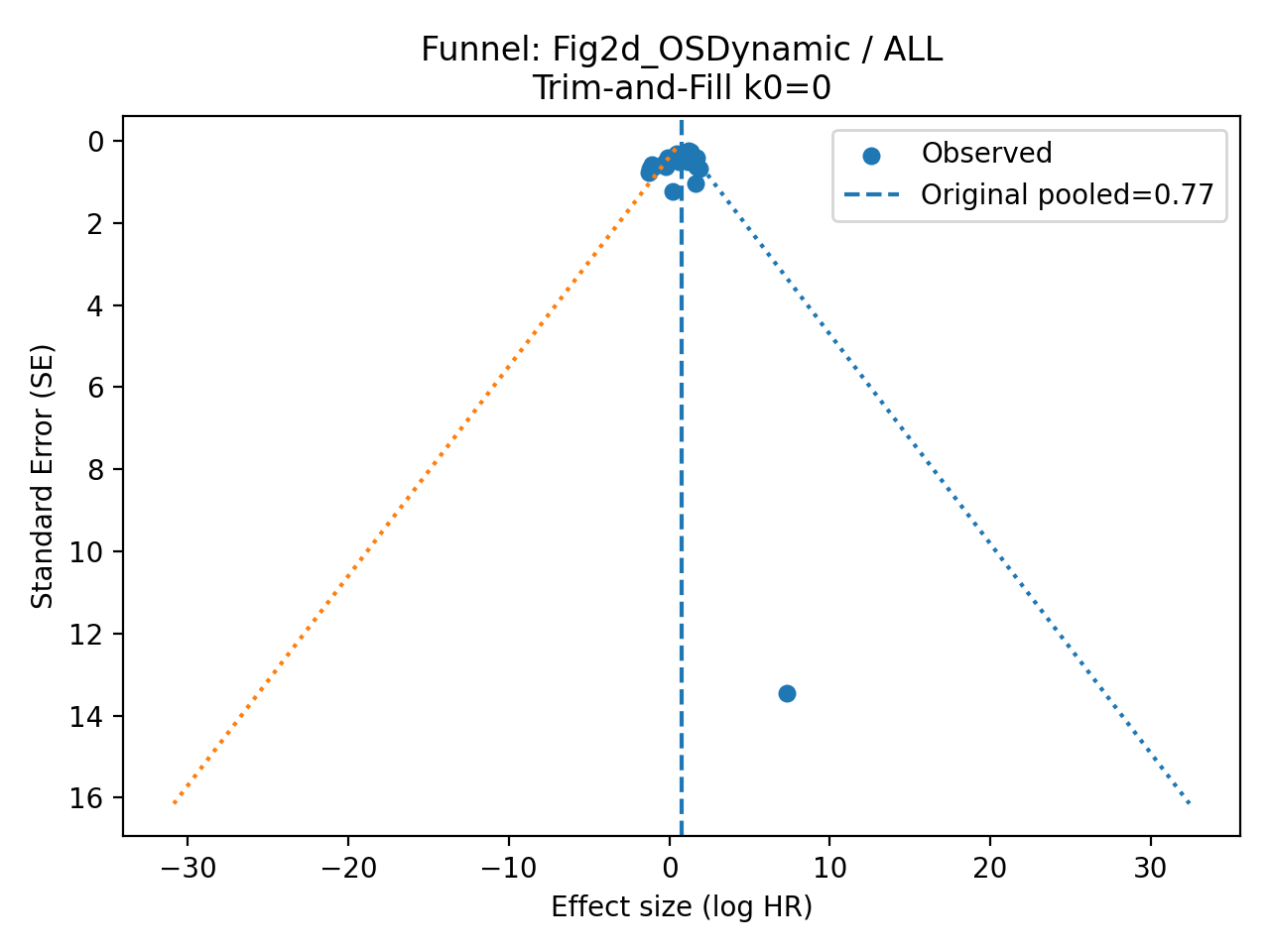

Supplement: Supplementary file 10 [file DataSheet2.zip › Fig2d_OSDynamic_TrimAndFill_ALL.png]

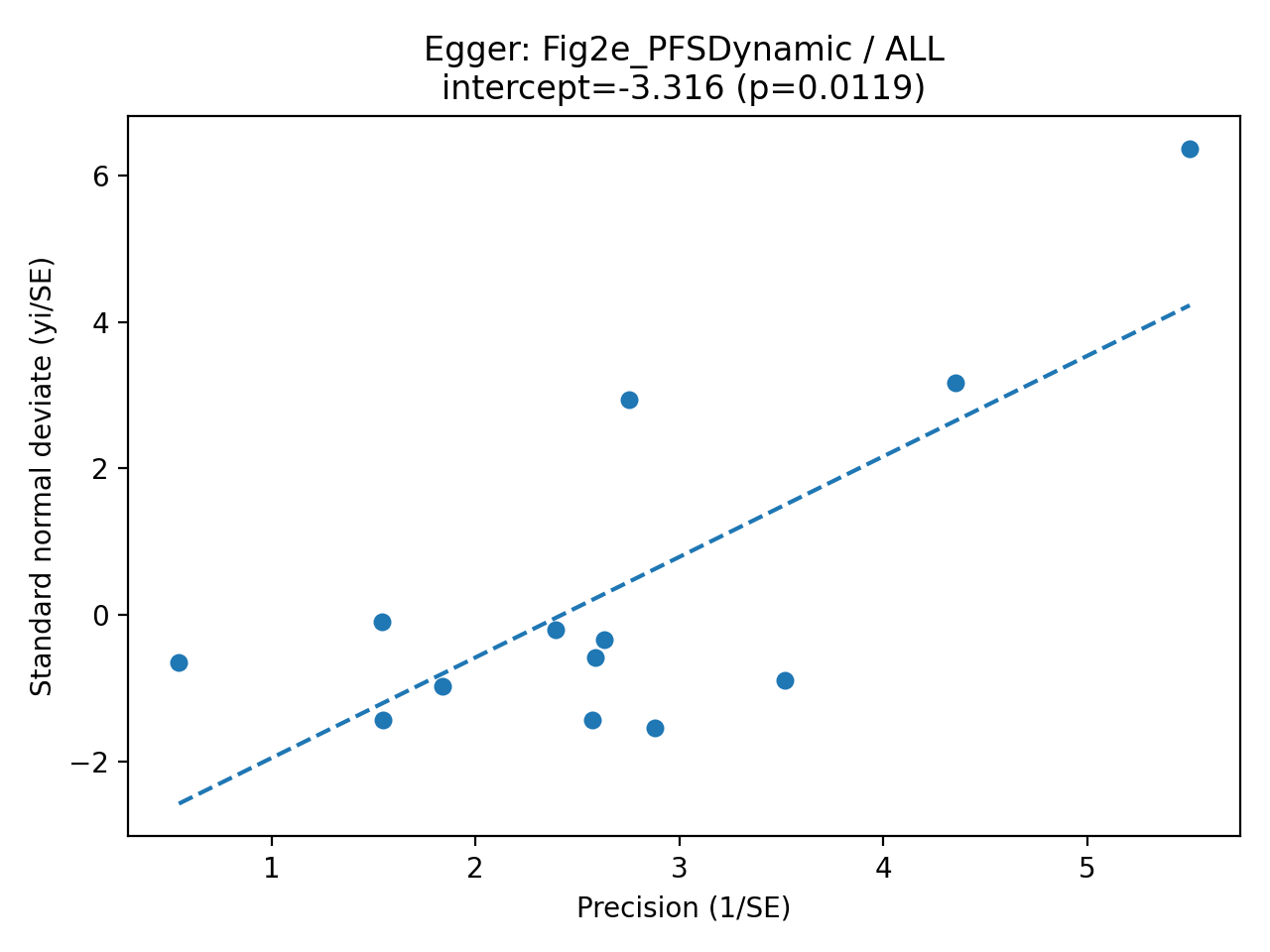

Supplement: Supplementary file 10 [file DataSheet2.zip › Fig2e_PFSDynamic_egger_ALL.png]

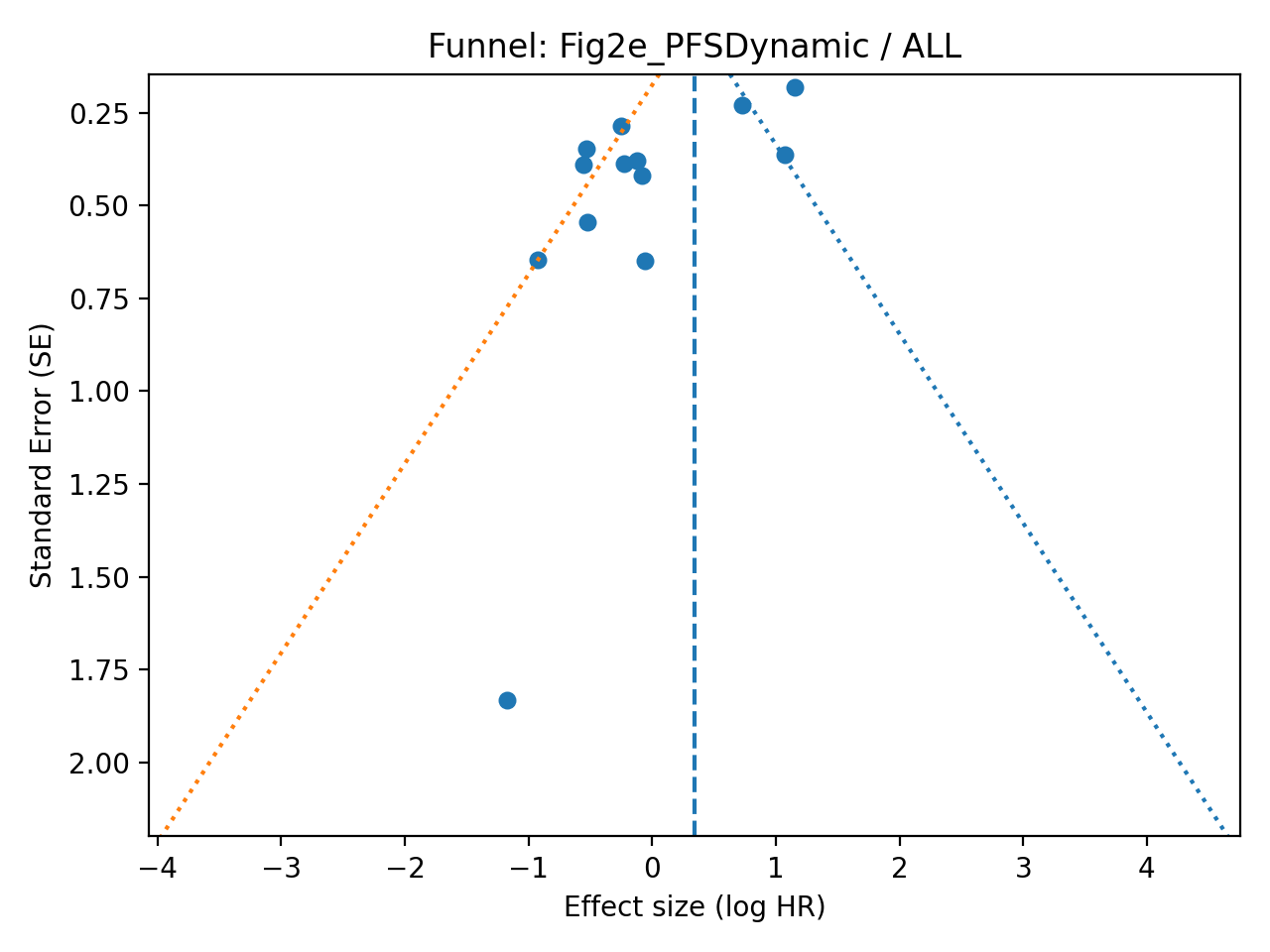

Supplement: Supplementary file 10 [file DataSheet2.zip › Fig2e_PFSDynamic_funnel_ALL.png]

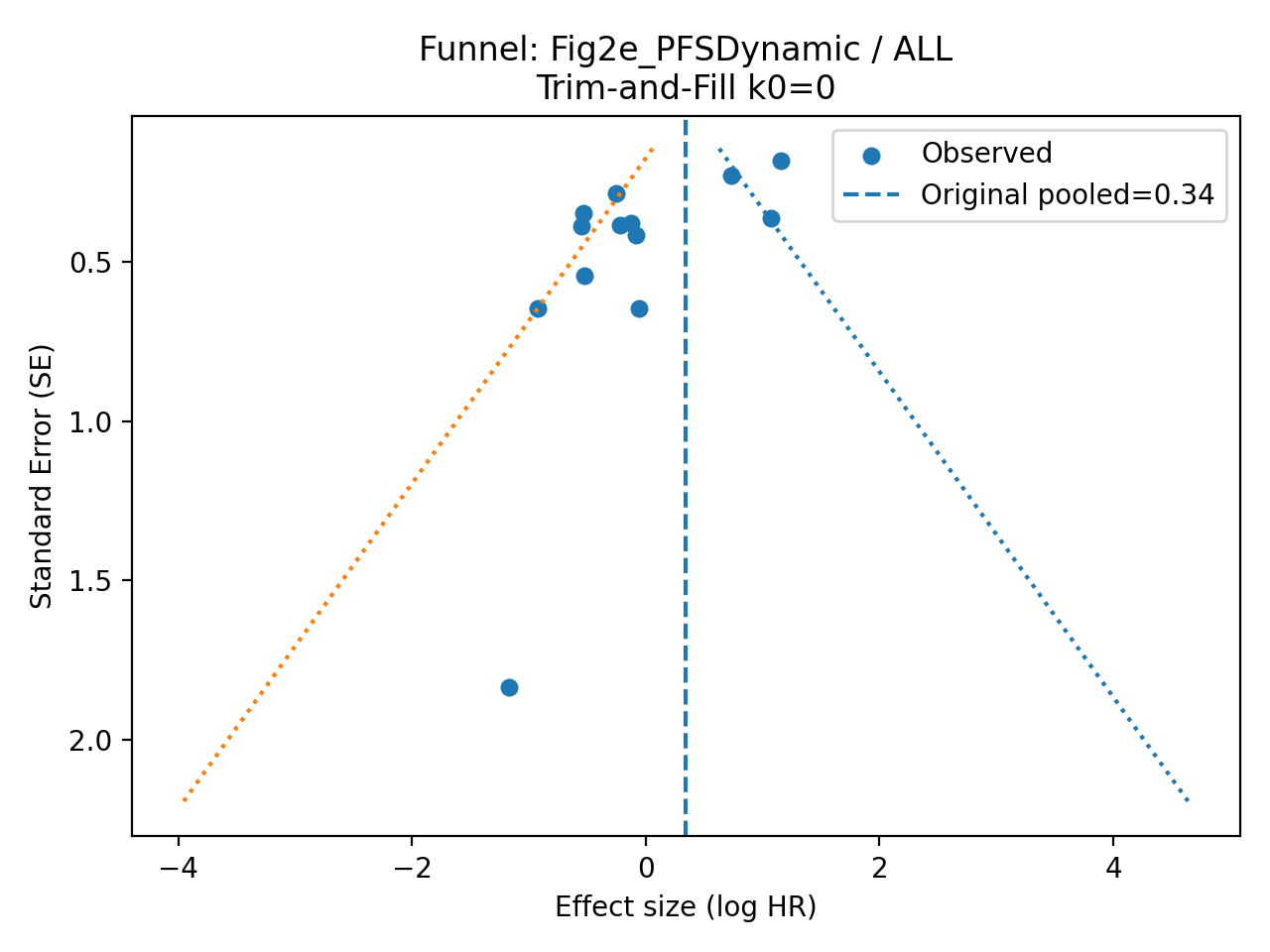

Supplement: Supplementary file 10 [file DataSheet2.zip › Fig2e_PFSDynamic_TrimAndFill_ALL.png]

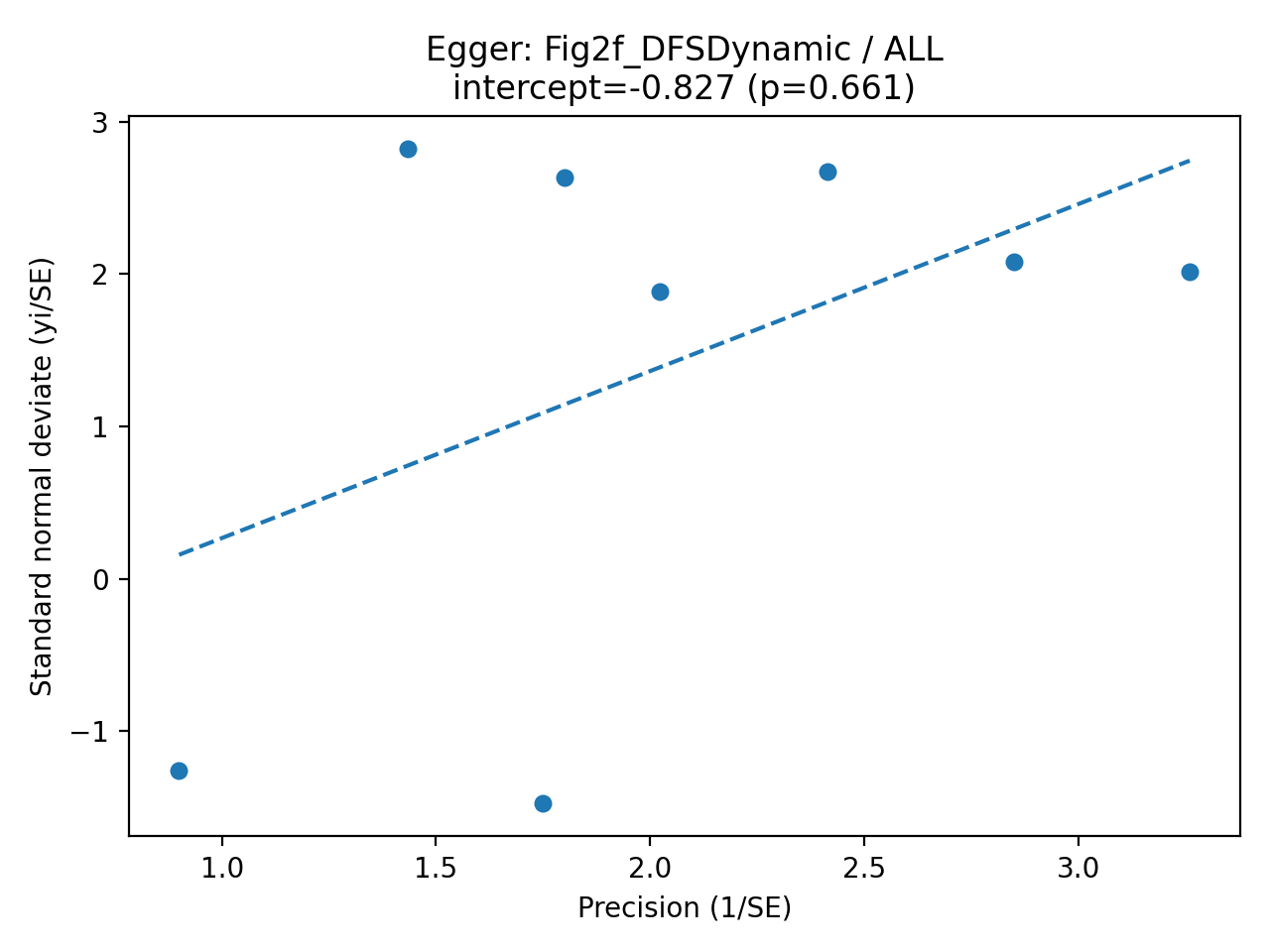

Supplement: Supplementary file 10 [file DataSheet2.zip › Fig2f_DFSDynamic_egger_ALL.png]

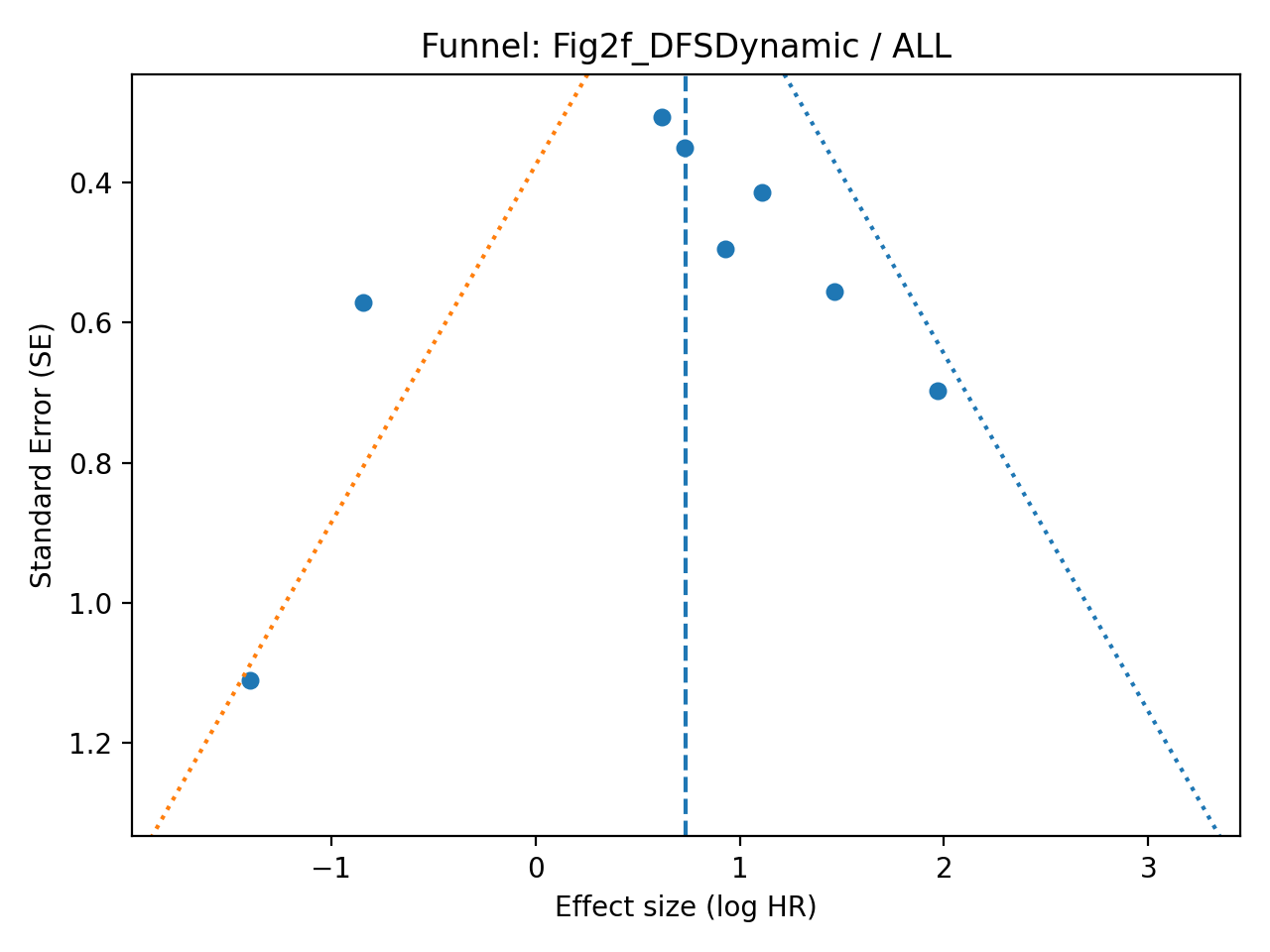

Supplement: Supplementary file 10 [file DataSheet2.zip › Fig2f_DFSDynamic_funnel_ALL.png]

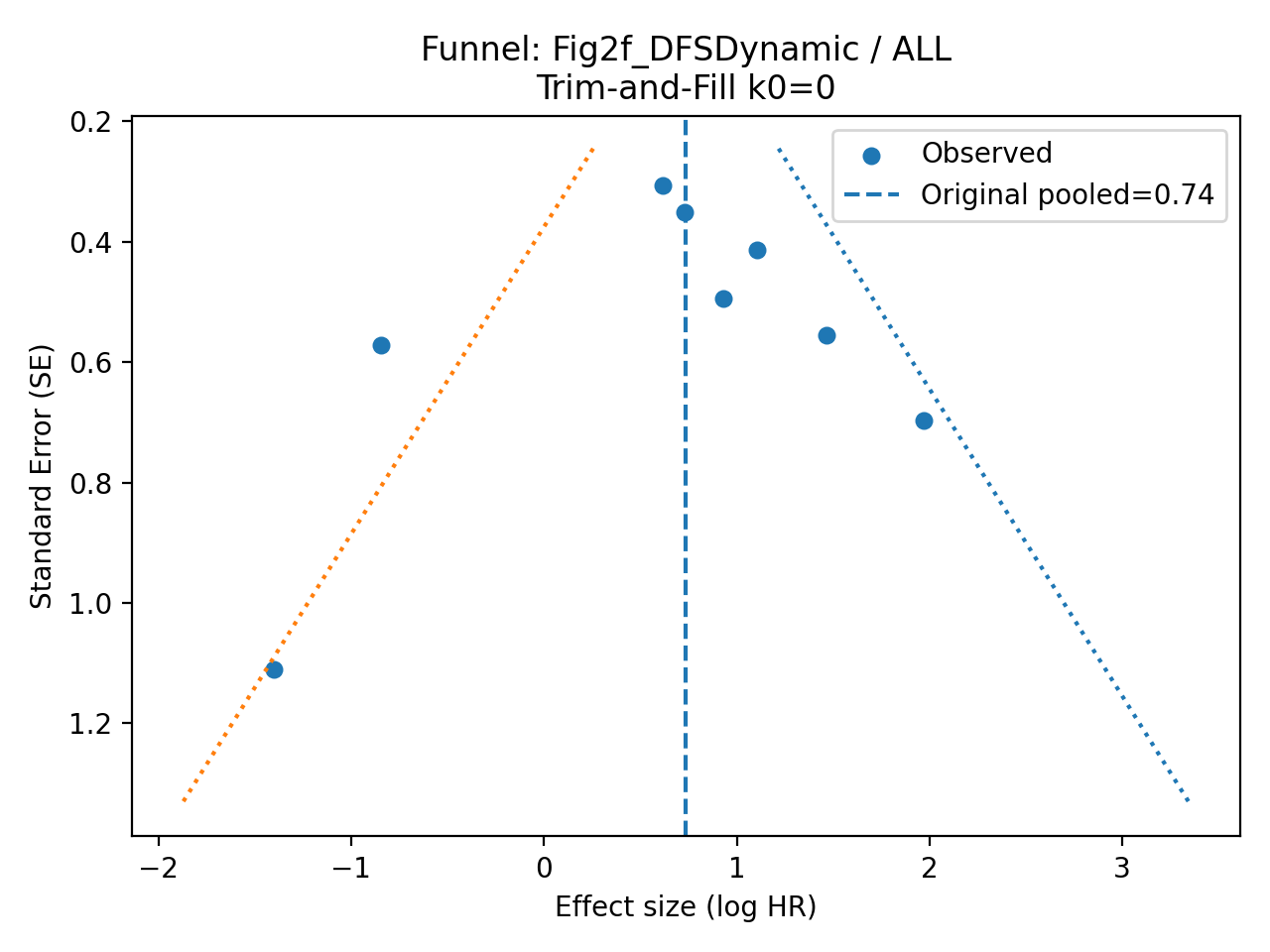

Supplement: Supplementary file 10 [file DataSheet2.zip › Fig2f_DFSDynamic_TrimAndFill_ALL.png]

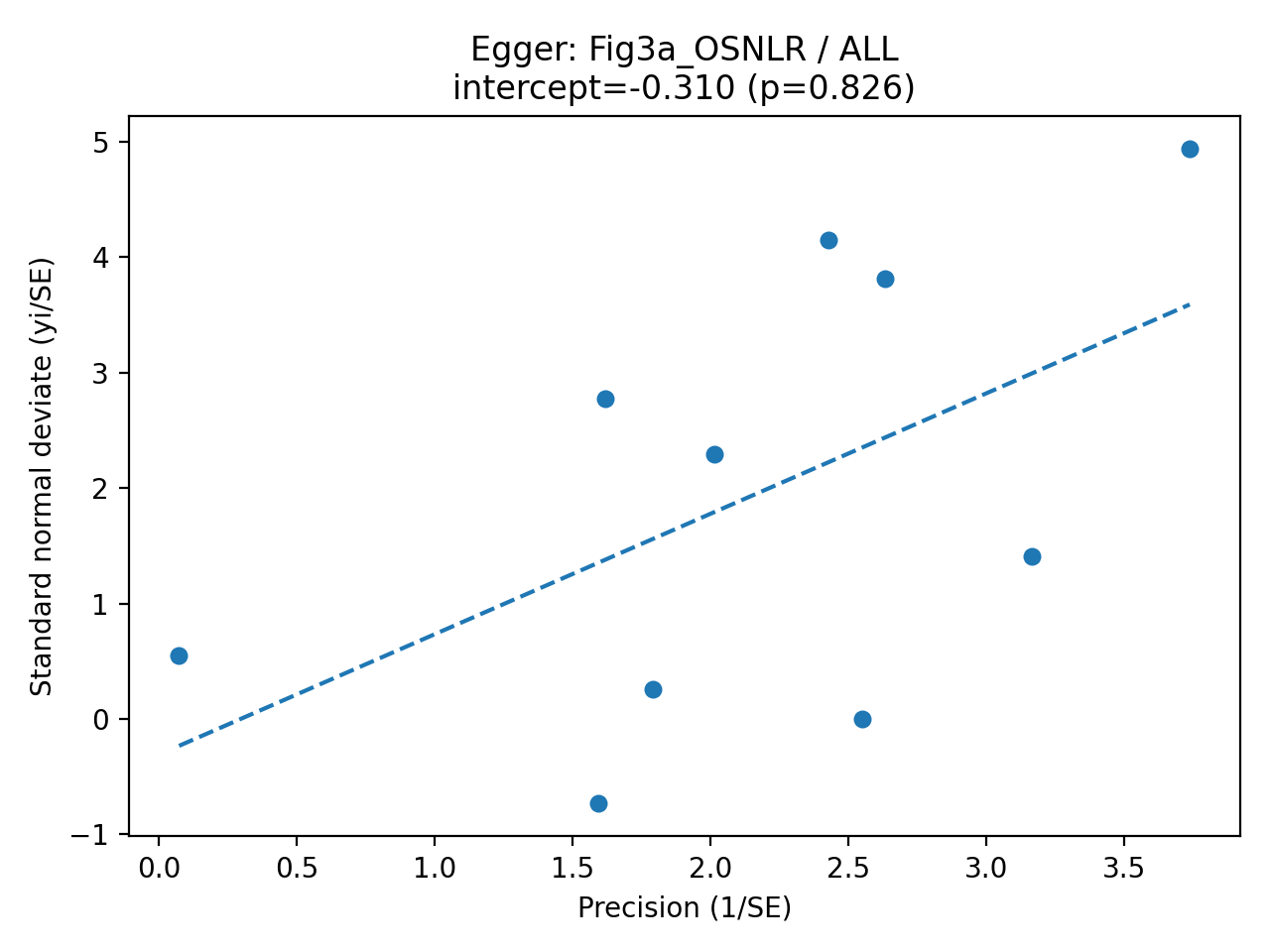

Supplement: Supplementary file 10 [file DataSheet2.zip › Fig3a_OSNLR_egger_ALL.png]

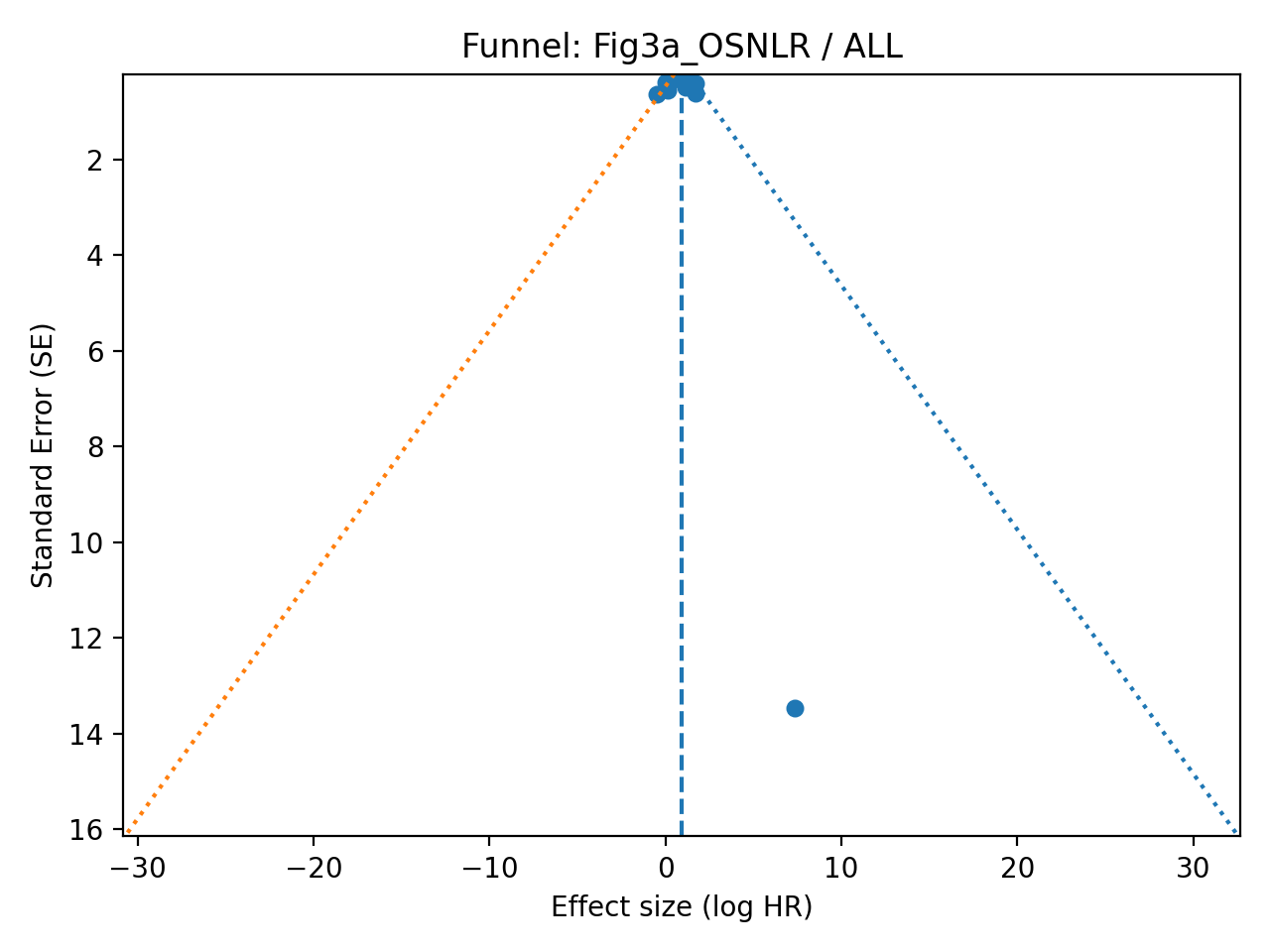

Supplement: Supplementary file 10 [file DataSheet2.zip › Fig3a_OSNLR_funnel_ALL.png]

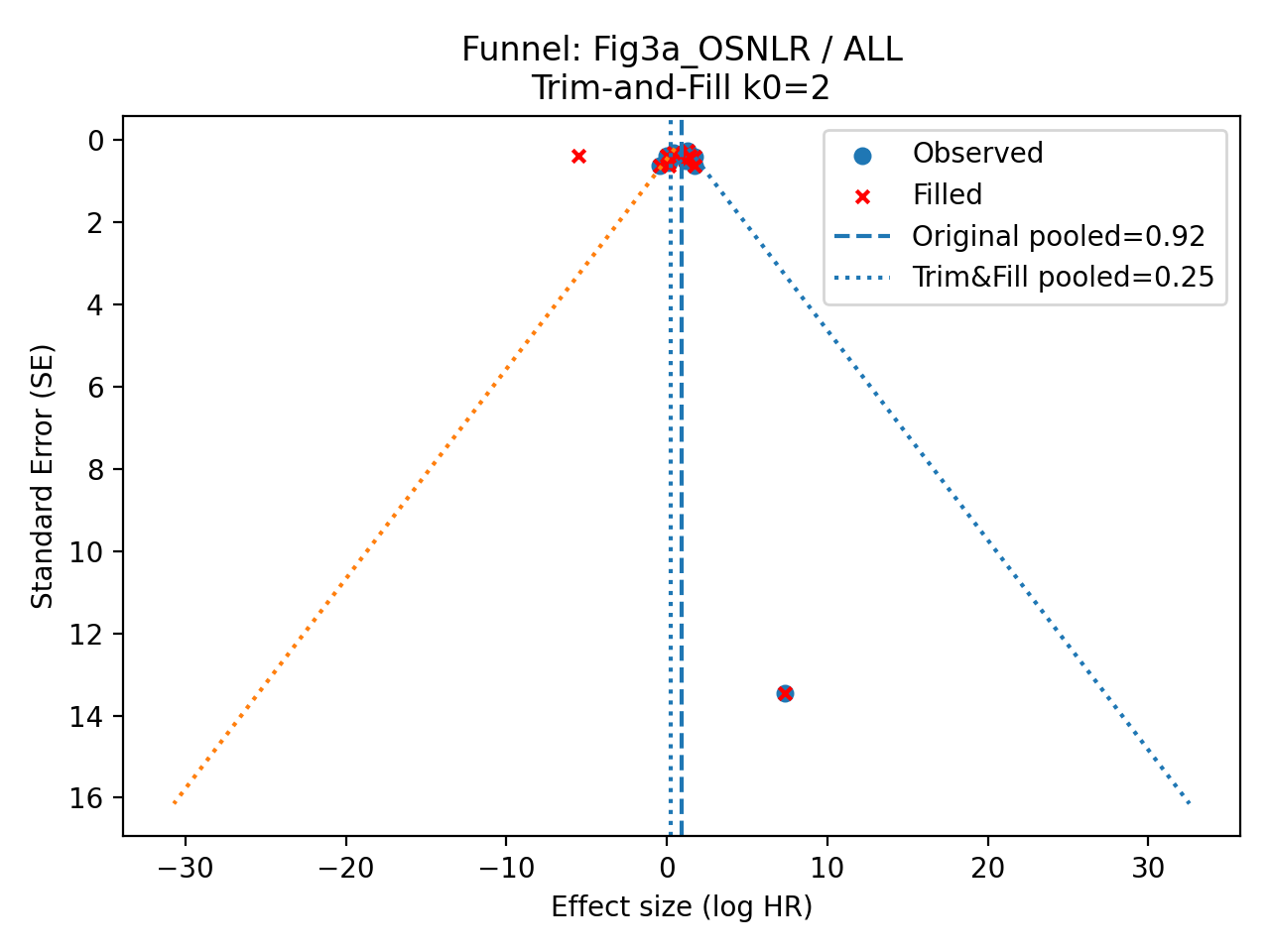

Supplement: Supplementary file 10 [file DataSheet2.zip › Fig3a_OSNLR_TrimAndFill_ALL.png]

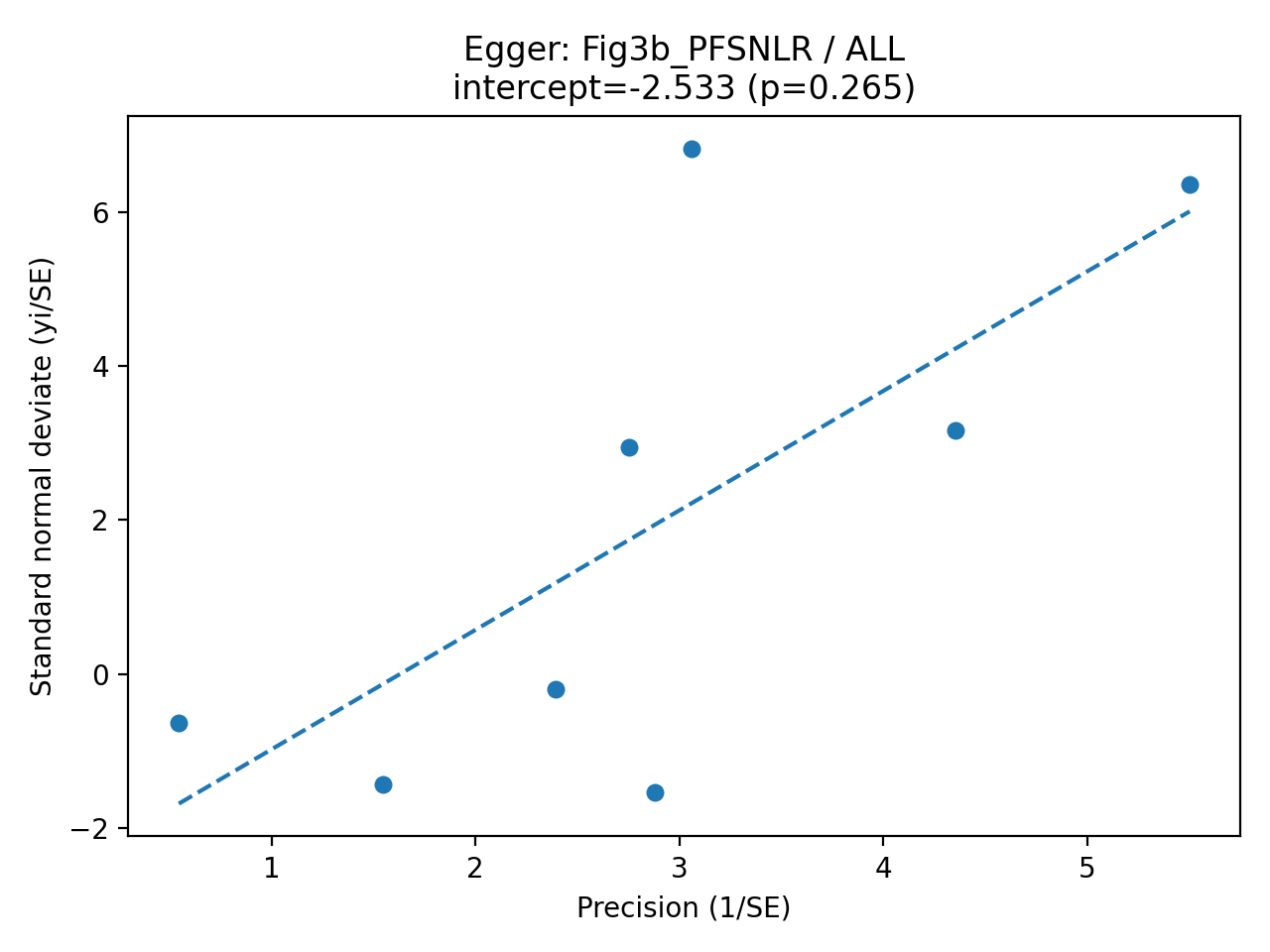

Supplement: Supplementary file 10 [file DataSheet2.zip › Fig3b_PFSNLR_egger_ALL.png]

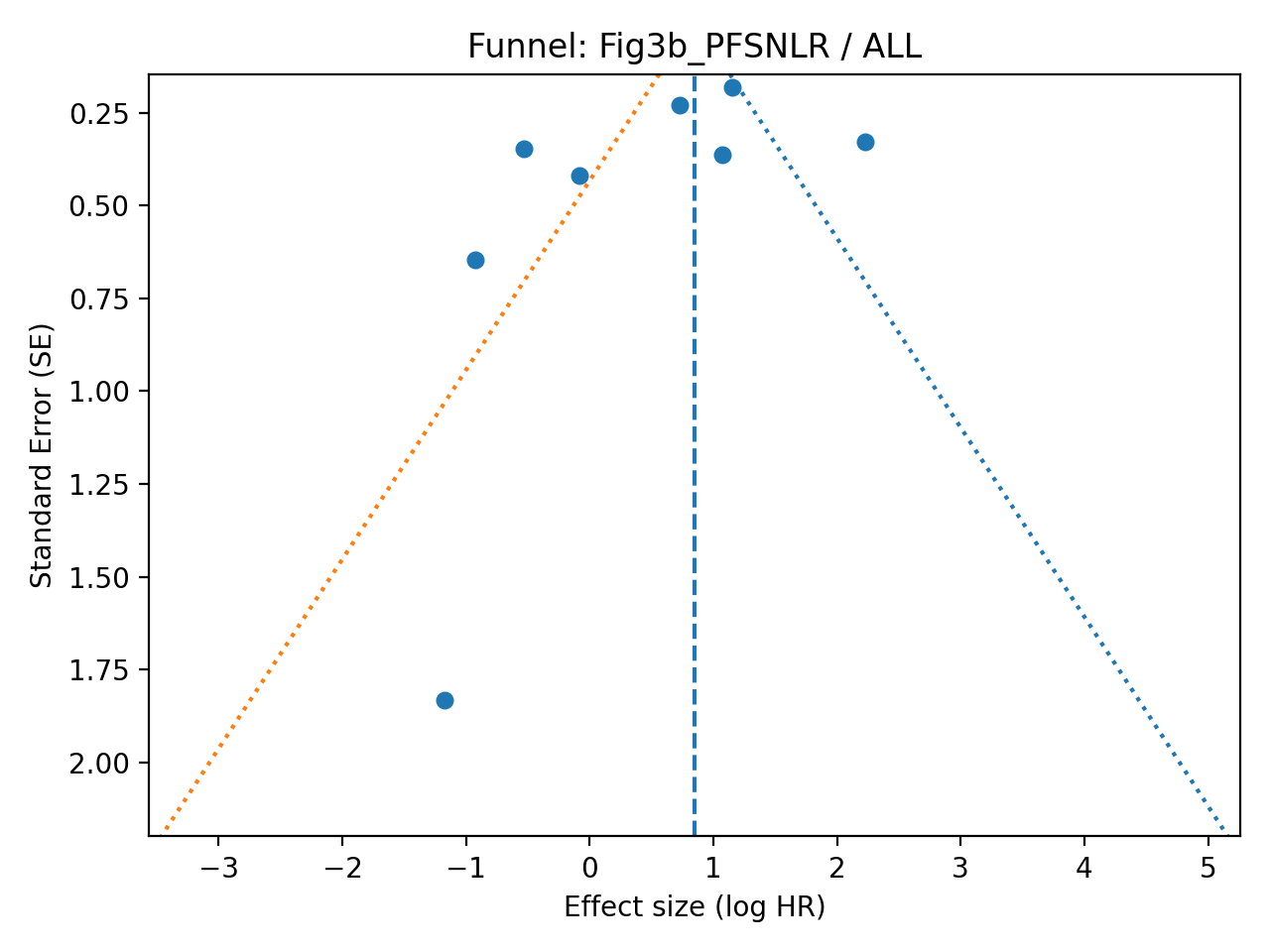

Supplement: Supplementary file 10 [file DataSheet2.zip › Fig3b_PFSNLR_funnel_ALL.png]

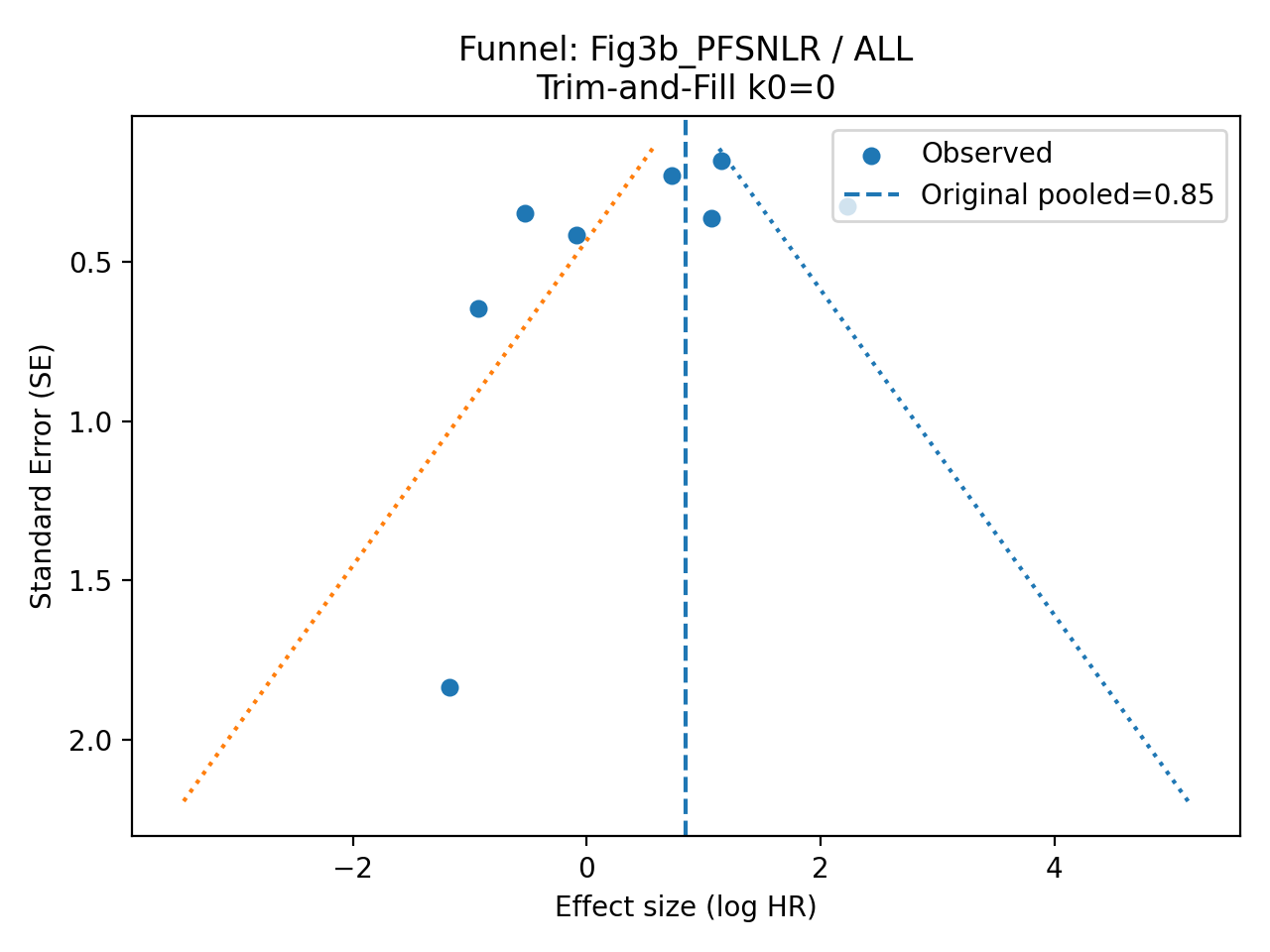

Supplement: Supplementary file 10 [file DataSheet2.zip › Fig3b_PFSNLR_TrimAndFill_ALL.png]

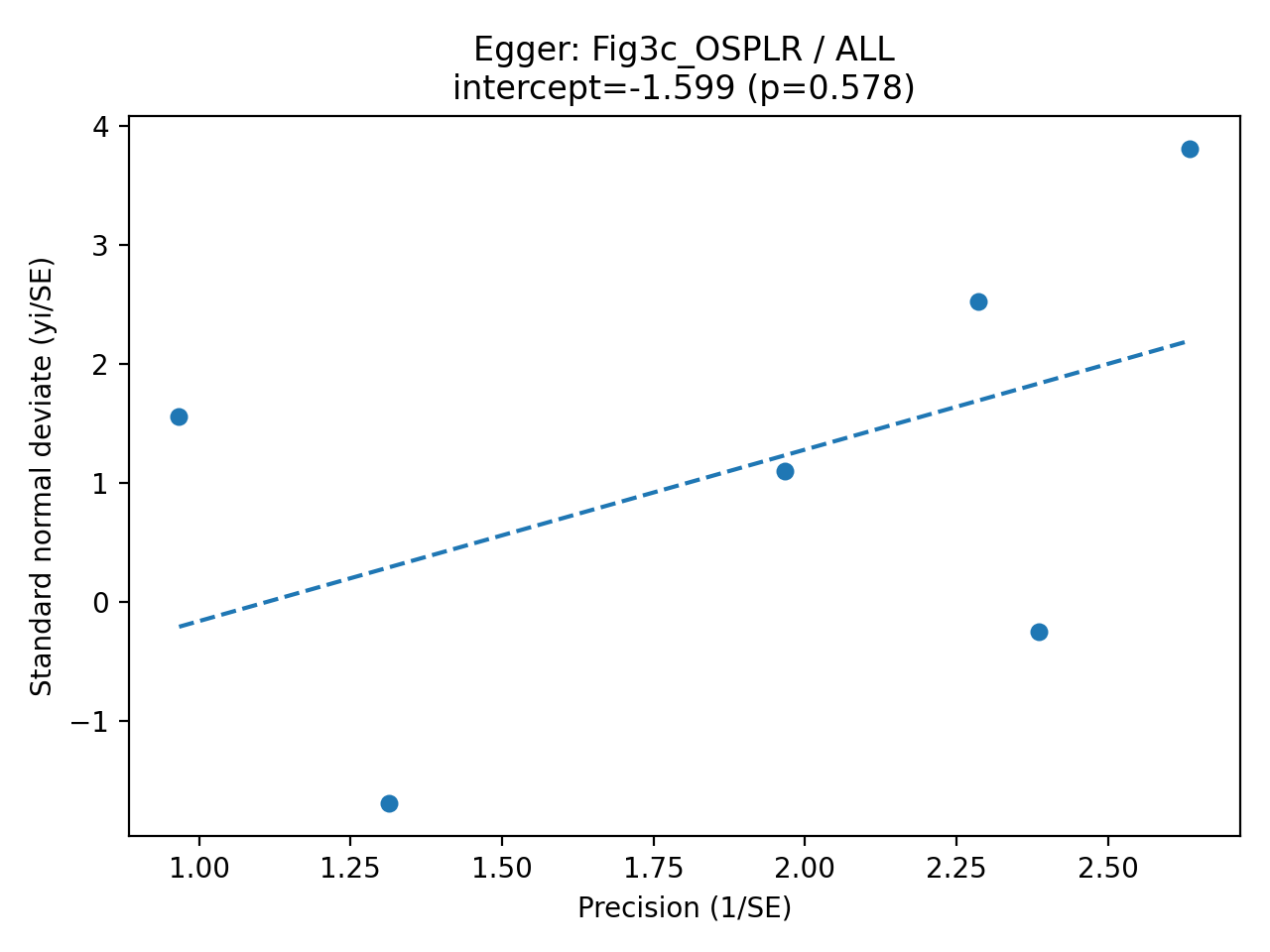

Supplement: Supplementary file 10 [file DataSheet2.zip › Fig3c_OSPLR_egger_ALL.png]

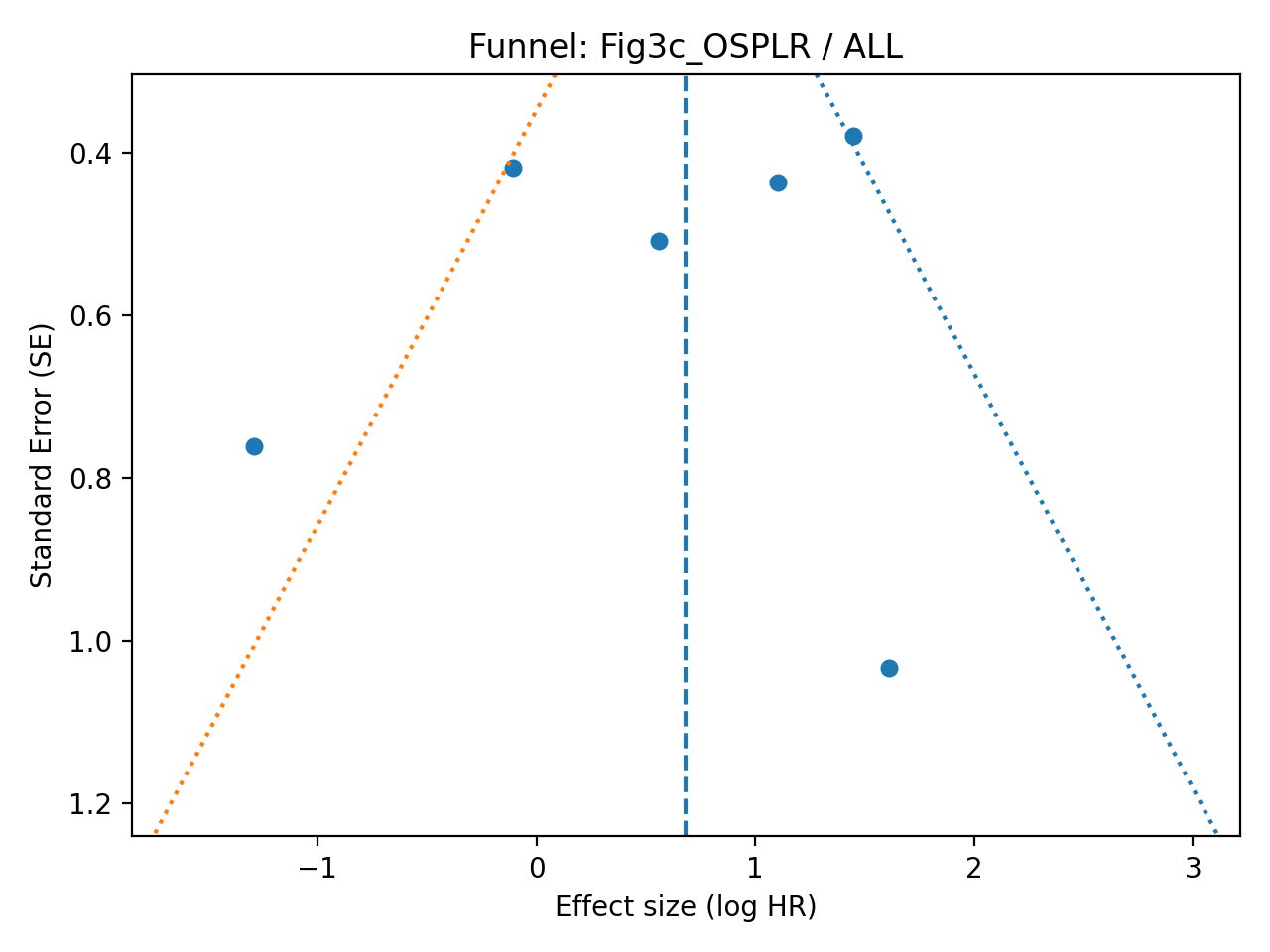

Supplement: Supplementary file 10 [file DataSheet2.zip › Fig3c_OSPLR_funnel_ALL.png]

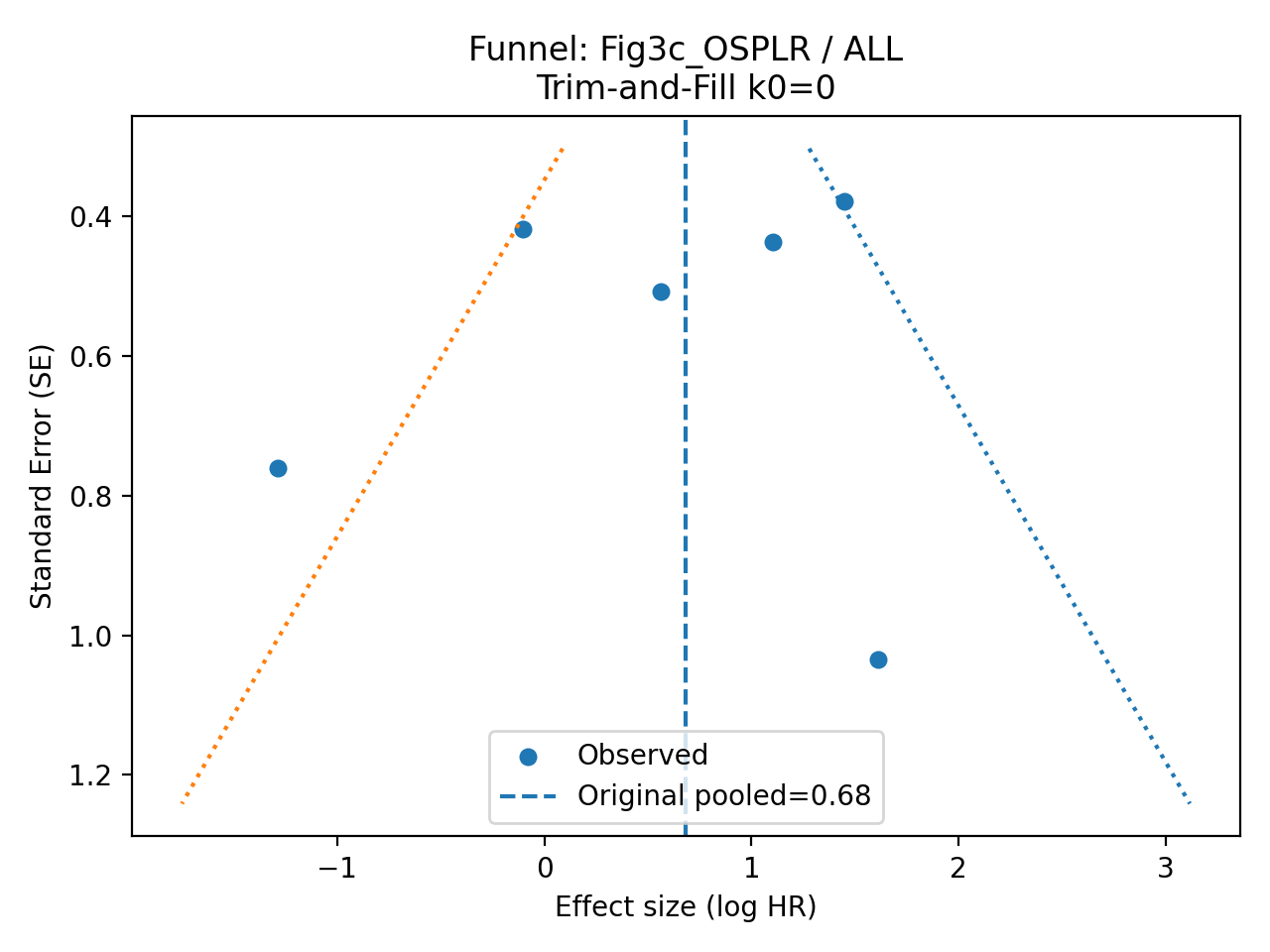

Supplement: Supplementary file 10 [file DataSheet2.zip › Fig3c_OSPLR_TrimAndFill_ALL.png]

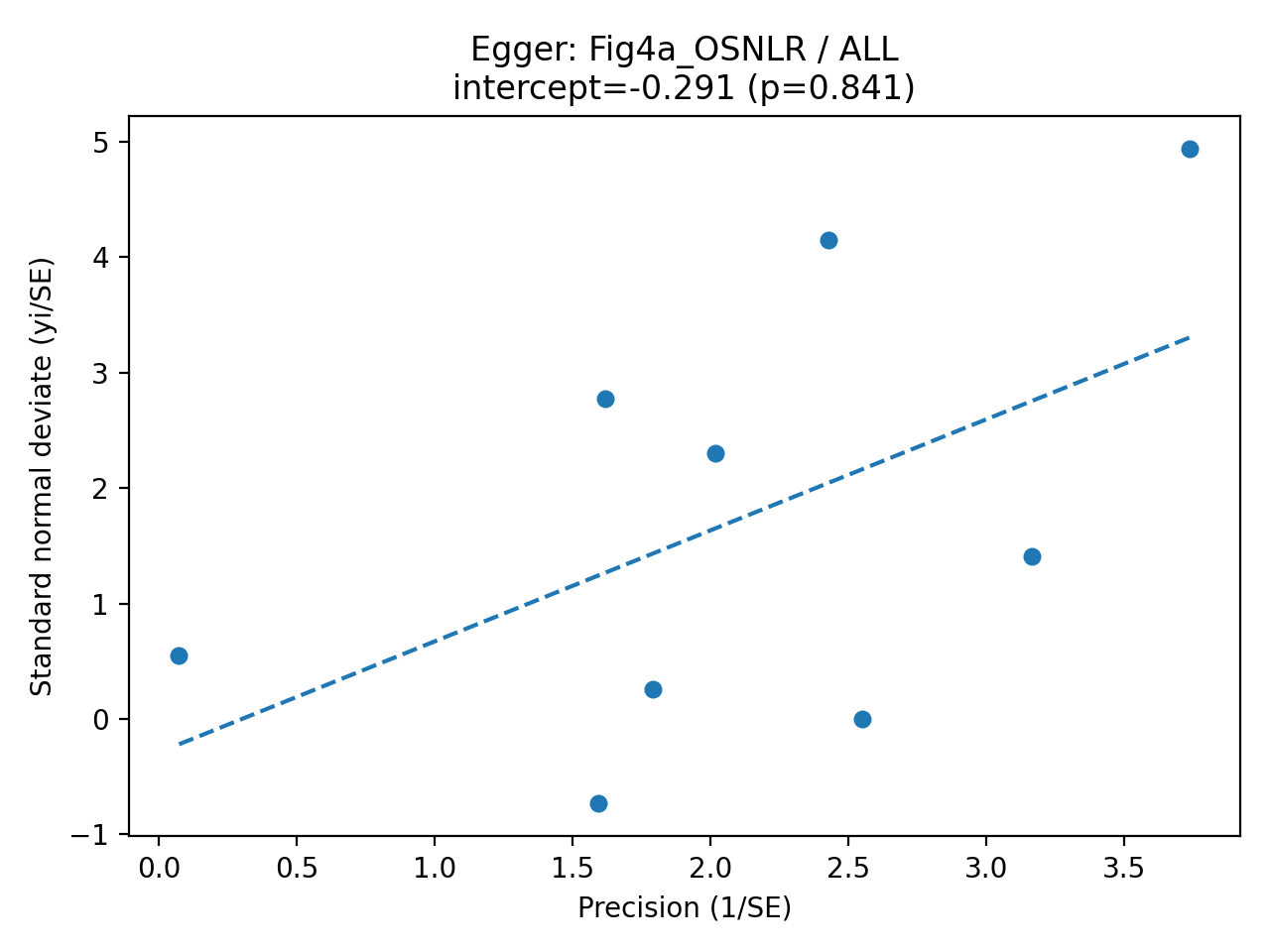

Supplement: Supplementary file 10 [file DataSheet2.zip › Fig4a_OSNLR_egger_ALL.png]

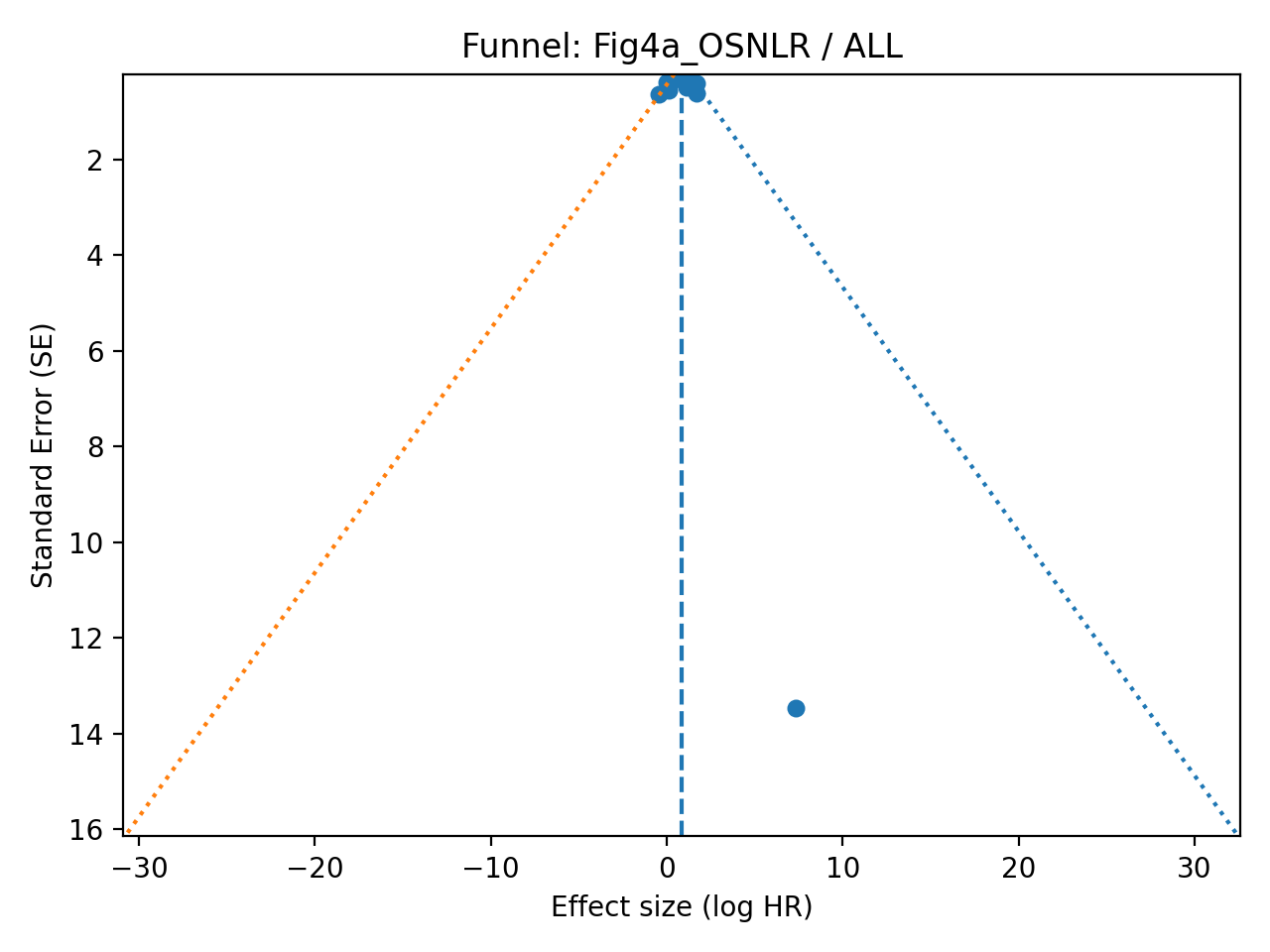

Supplement: Supplementary file 10 [file DataSheet2.zip › Fig4a_OSNLR_funnel_ALL.png]

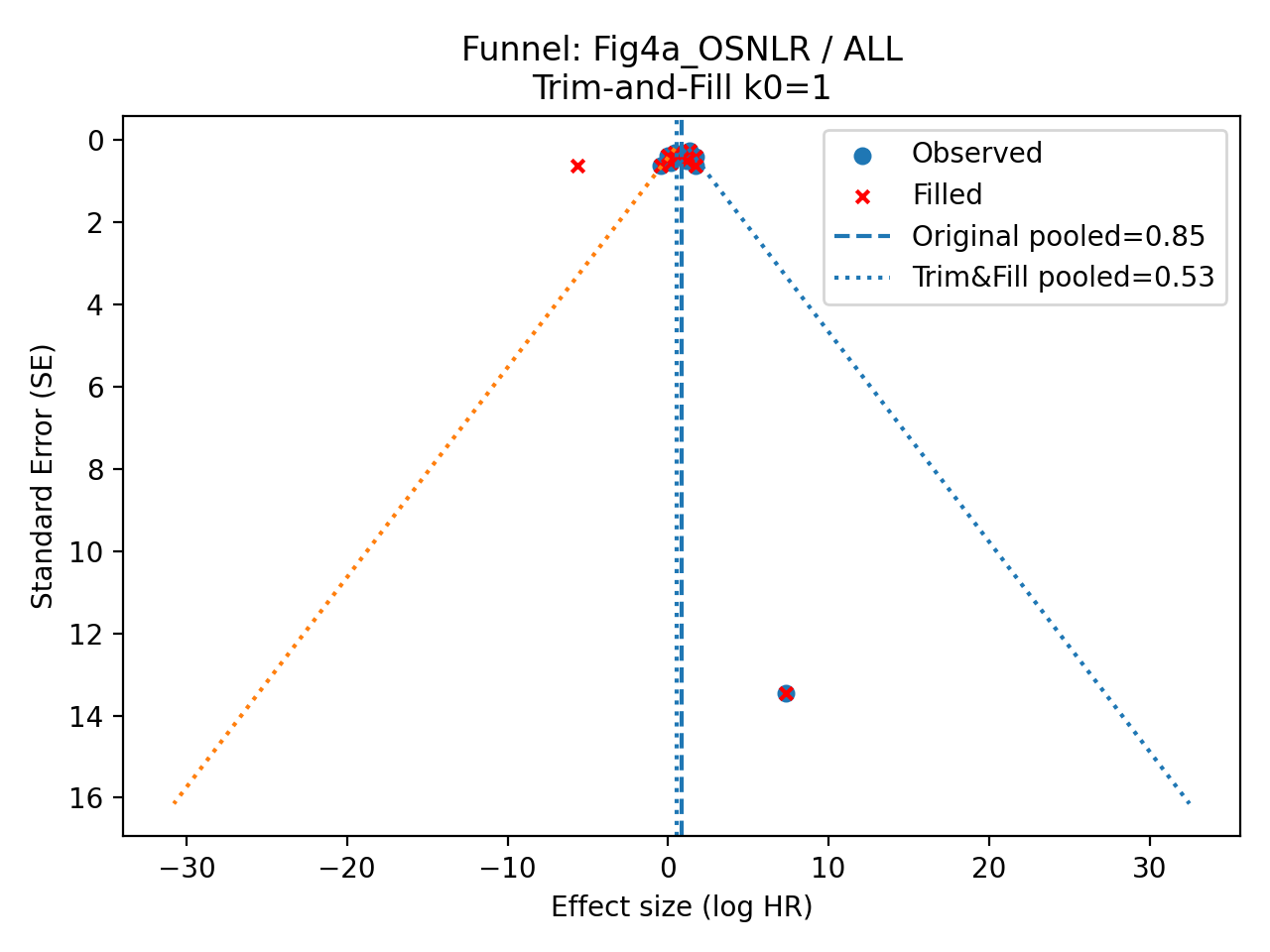

Supplement: Supplementary file 10 [file DataSheet2.zip › Fig4a_OSNLR_TrimAndFill_ALL.png]

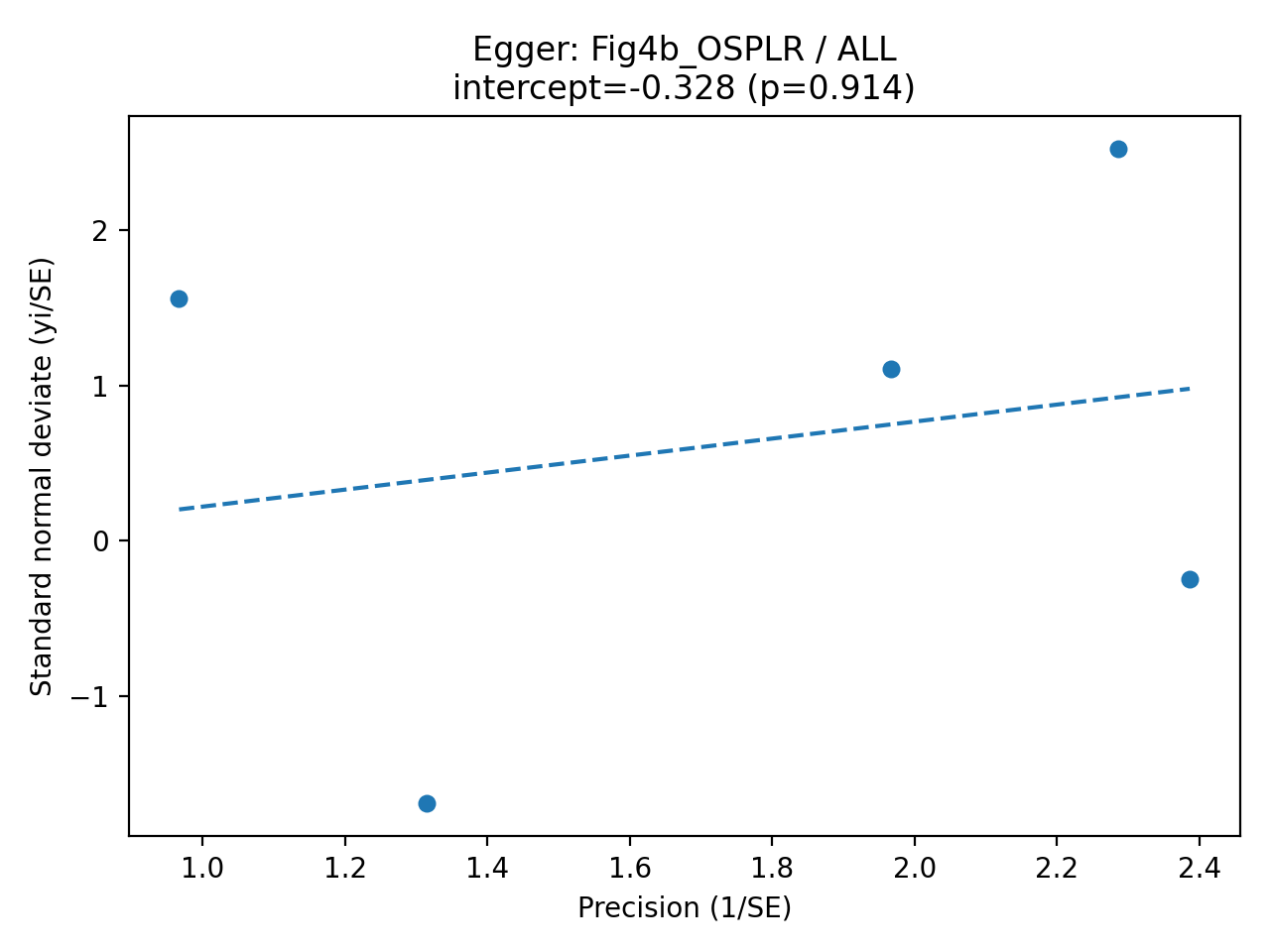

Supplement: Supplementary file 10 [file DataSheet2.zip › Fig4b_OSPLR_egger_ALL.png]

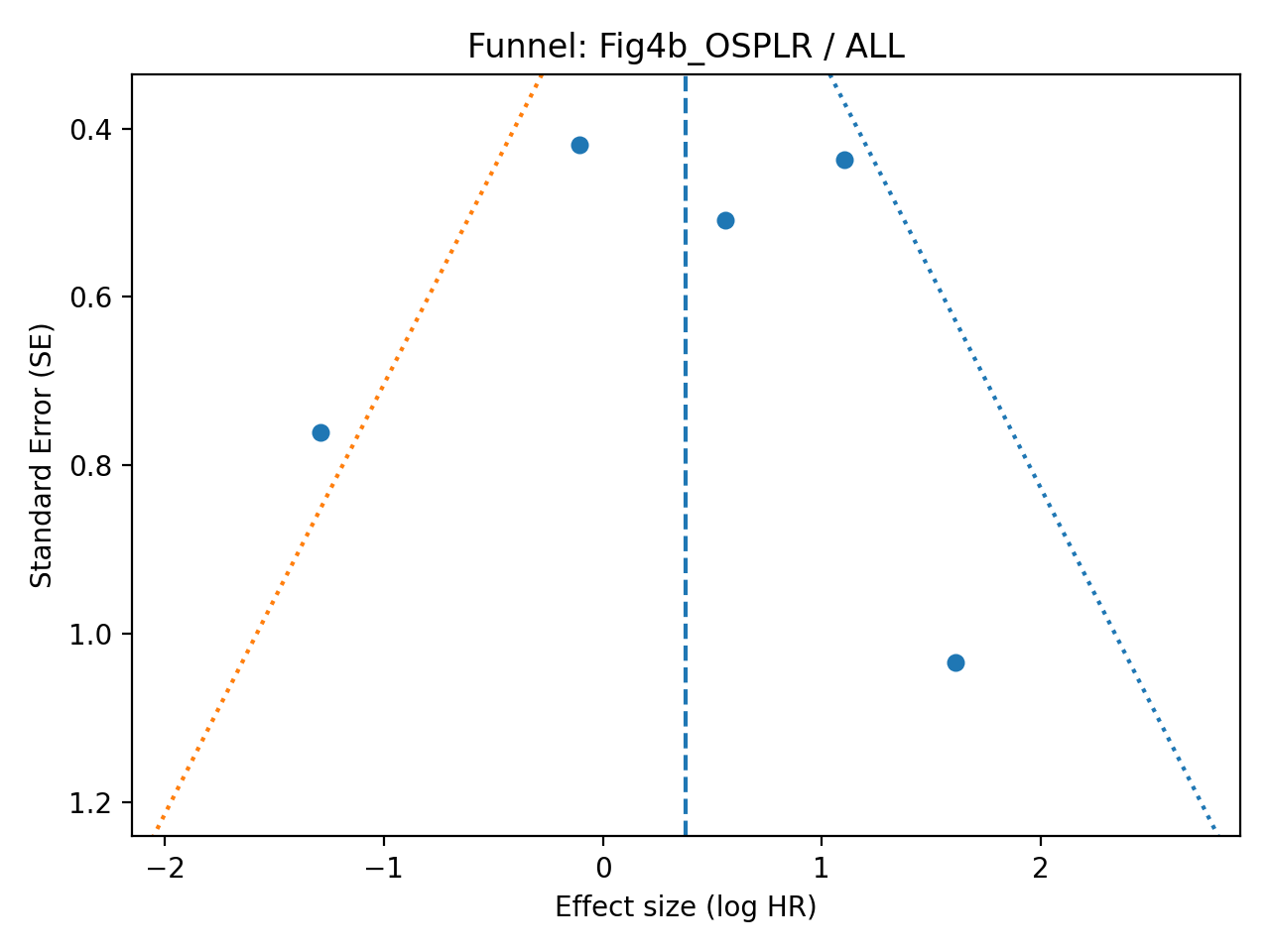

Supplement: Supplementary file 10 [file DataSheet2.zip › Fig4b_OSPLR_funnel_ALL.png]

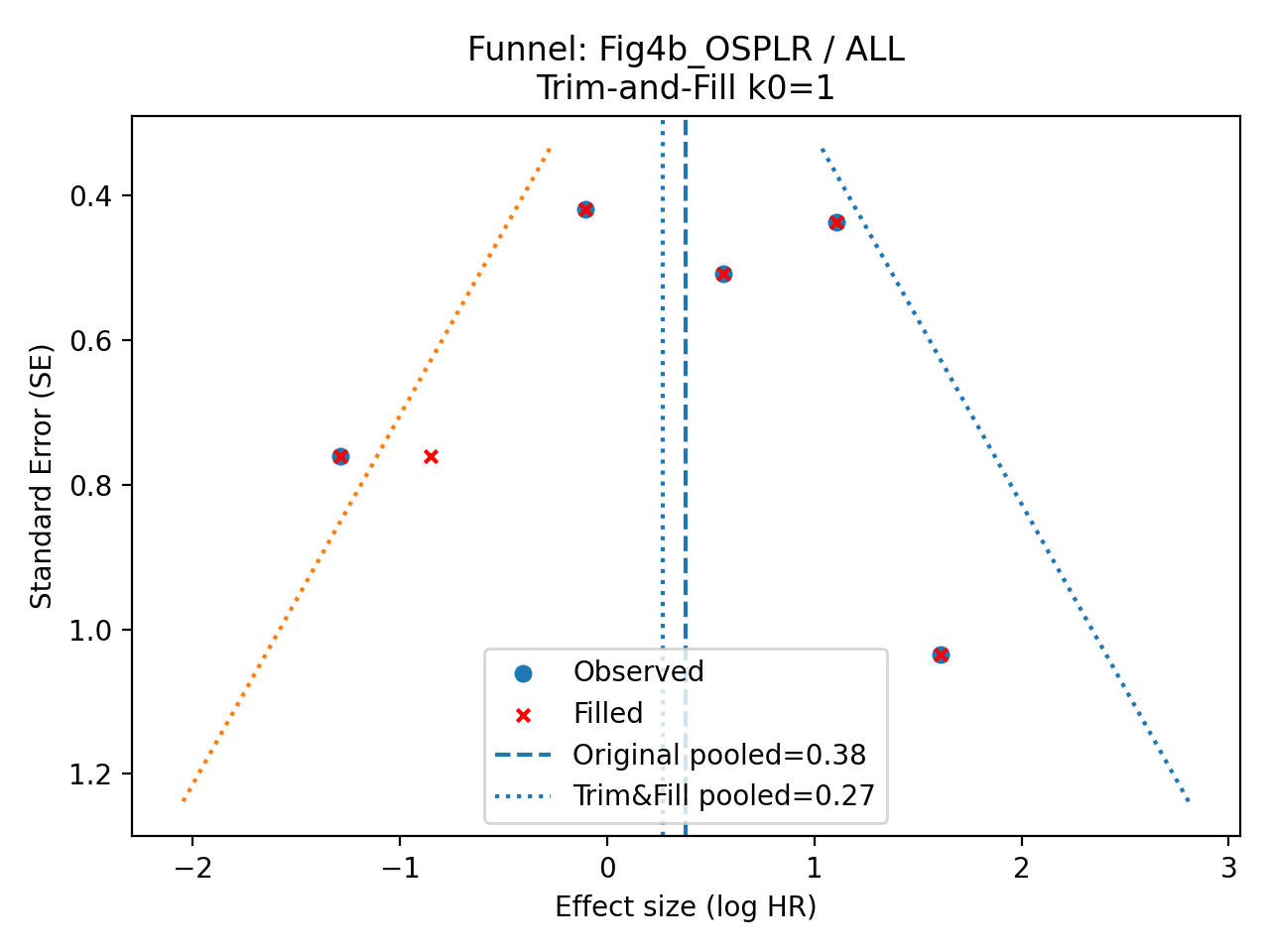

Supplement: Supplementary file 10 [file DataSheet2.zip › Fig4b_OSPLR_TrimAndFill_ALL.png]

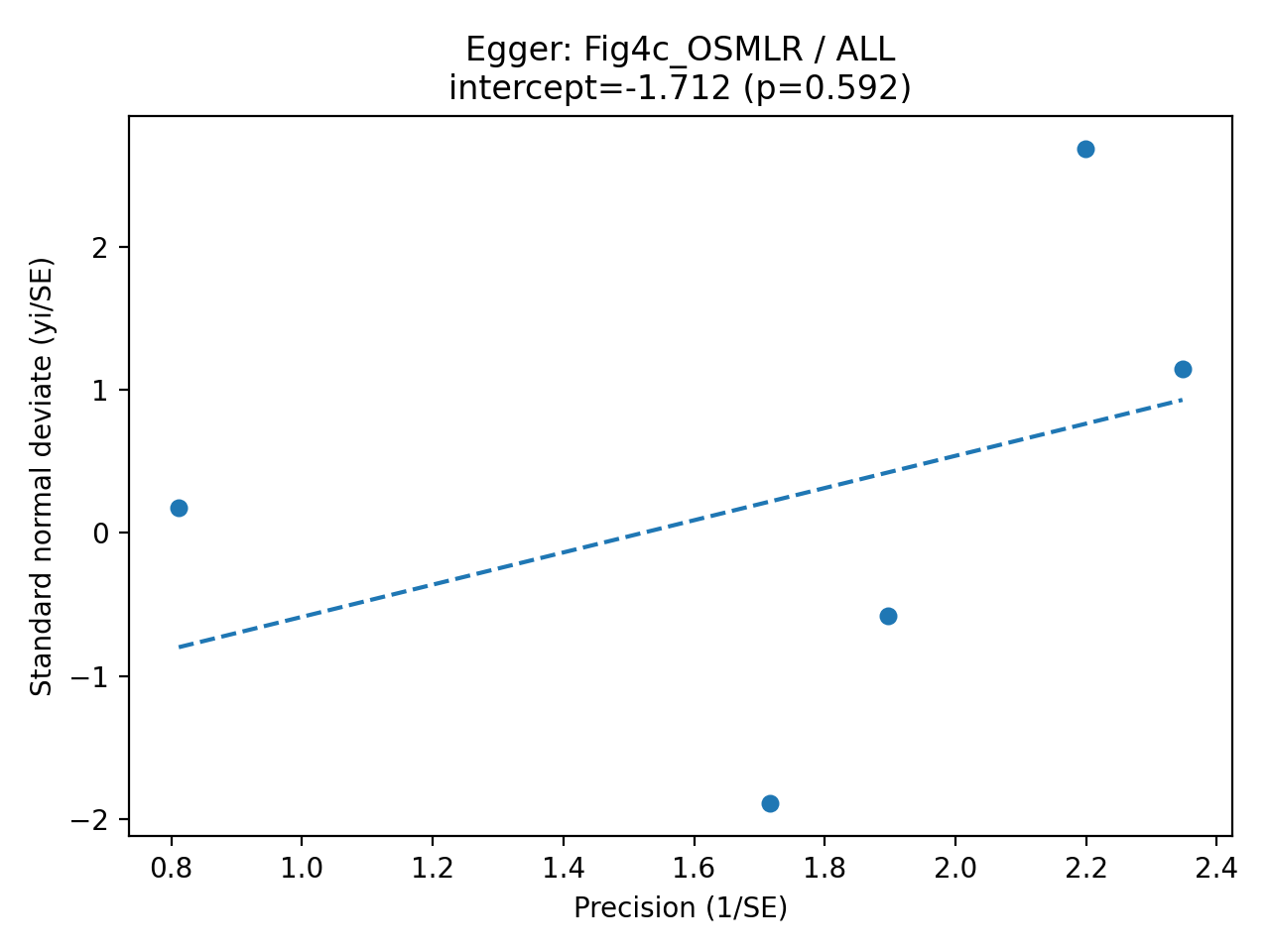

Supplement: Supplementary file 10 [file DataSheet2.zip › Fig4c_OSMLR_egger_ALL.png]

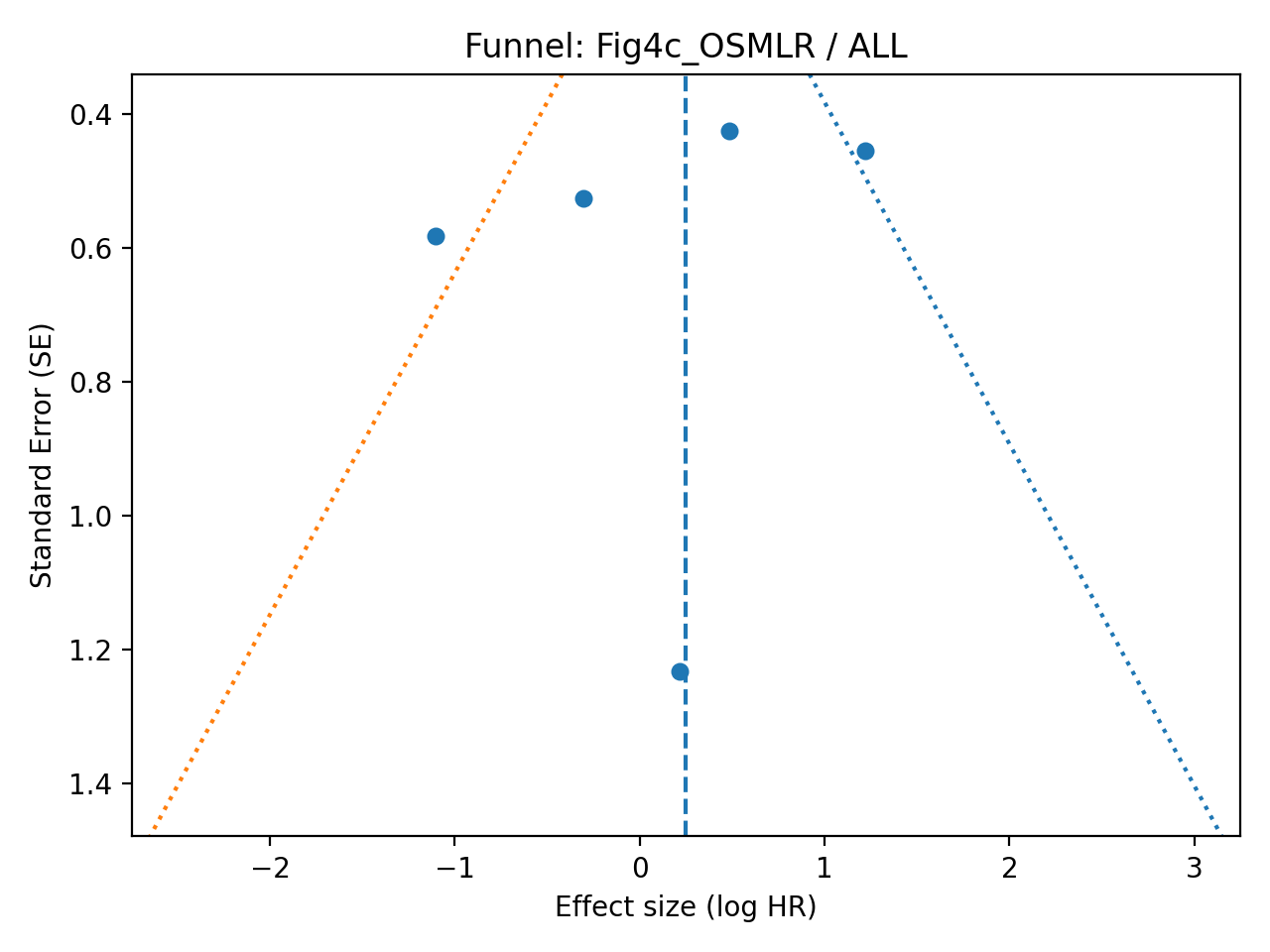

Supplement: Supplementary file 10 [file DataSheet2.zip › Fig4c_OSMLR_funnel_ALL.png]
