# Supplementary material for: Can posttreatment blood inflammatory markers predict poor survival in gynecologic cancer?: a systematic review and meta-analysis
Source: Front Immunol. 2025 Oct 21;16:1676838. doi: 10.3389/fimmu.2025.1676838 (PMC12583213; doi:10.3389/fimmu.2025.1676838)
Supplement: Supplementary file 11 [file Table1.docx]

| **Can posttreatment blood inflammatory markers predict poor survival in gynecologic cancer?: A systematic review and meta-analysis**  **Table S1**. Detailed overview of keywords used for search strategy   \| **Database** \| **Coverage**  **Platform** \| **Final search data** \| **Language restrictions** \| **Applied filters/limits** \| \| --- \| --- \| --- \| --- \| --- \| \| PubMed \| MEDLINE \| 2024.09.13 \| English only \| meta-analysis[Filter] OR systematic review[Filter] \| \| Embase \| Elsevier Embase \| 2024.09.13 \| English only \| meta-analysis[Filter] OR systematic review[Filter] \| \| Cochrane library \| Cochrane Reviews \| 2024.09.13 \| English only \| meta-analysis[Filter] OR systematic review[Filter] \| | | | |
| --- | --- | --- | --- | --- | --- | --- | --- | --- | --- | --- | --- | --- | --- | --- | --- | --- | --- | --- | --- | --- | --- | --- | --- |
| **Database** | **Search** | **Keywords** |  |
| **PubMed** | #1 | "Uterine Cervical Neoplasms"[Mesh] |  |
|  | #2 | "Uterine Cervical Neoplasms"[TW] OR "Cervical Neoplasm, Uterine"[TW] OR "Neoplasm, Uterine Cervical"[TW] OR "Uterine Cervical Neoplasm"[TW] OR "Neoplasms, Cervical"[TW] OR "Cervical Neoplasms"[TW] OR "Cervical Neoplasm"[TW] OR "Neoplasms, Cervix"[TW] OR "Cervix Neoplasm"[TW] OR "Neoplasm, Cervix"[TW] OR "Cervix Neoplasms"[TW] OR "Cancer of the Uterine Cervix"[TW] OR "Cancer of the Cervix"[TW] OR "Cervical Cancer"[TW] OR "Cancer, Cervical"[TW] OR "Cervical Cancers"[TW] OR "Uterine Cervical Cancer"[TW] OR "Cancer, Uterine Cervical"[TW] OR "Cervical Cancer, Uterine"[TW] OR "Uterine Cervical Cancers"[TW] OR "Cancer of Cervix"[TW] OR "Cervix Cancer"[TW] OR "Cancer, Cervix"[TW] OR "cervical adenocarcinoma"[TW] |  |
|  | #3 | "Ovarian Neoplasms"[Mesh] |  |
|  | #4 | "Ovarian Neoplasms"[TW] OR "Neoplasm, Ovarian"[TW] OR "Ovarian Neoplasm"[TW] OR "Ovary Neoplasms"[TW] OR "Neoplasm, Ovary"[TW] OR "Neoplasms, Ovary"[TW] OR "Ovary Neoplasm"[TW] OR "Neoplasms, Ovarian"[TW] OR "Ovary Cancer"[TW] OR "Cancer, Ovary"[TW] OR "Cancers, Ovary"[TW] OR "Ovary Cancers"[TW] OR "Ovarian Cancer"[TW] OR "Cancer, Ovarian"[TW] OR "Cancers, Ovarian"[TW] OR "Ovarian Cancers"[TW] OR "Cancer of Ovary"[TW] OR "Cancer of the Ovary"[TW] OR "ovarian clear cell carcinoma "[TW] OR "Mutinous ovarian carcinoma"[TW] |  |
|  | #5 | "Endometrial Neoplasms"[Mesh] |  |
|  | #6 | "Endometrial Neoplasms"[TW] OR "Endometrial Neoplasm"[TW] OR "Neoplasm, Endometrial"[TW] OR "Neoplasms, Endometrial"[TW] OR "Endometrial Carcinoma"[TW] OR "Carcinoma, Endometrial"[TW] OR "Carcinomas, Endometrial"[TW] OR "Endometrial Carcinomas"[TW] OR "Endometrial Cancer"[TW] OR "Cancer, Endometrial"[TW] OR "Cancers, Endometrial"[TW] OR "Endometrial Cancers"[TW] OR "Endometrium Cancer"[TW] OR "Cancer, Endometrium"[TW] OR "Cancers, Endometrium"[TW] OR "Cancer of the Endometrium"[TW] OR "Carcinoma of Endometrium"[TW] OR "Endometrium Carcinoma"[TW] OR "Endometrium Carcinomas"[TW] OR "Cancer of Endometrium"[TW] OR "Endometrium Cancers"[TW] |  |
|  | #7 | "Carcinoma, Ovarian Epithelial"[Mesh] |  |
|  | #8 | "Carcinoma, Ovarian Epithelial"[TW] OR "Epithelial Carcinoma, Ovarian"[TW] OR "Ovarian Epithelial Carcinomas"[TW] OR "Epithelial Ovarian Cancer"[TW] OR "Ovarian Epithelial Cancer"[TW] OR "Cancer, Ovarian Epithelial"[TW] OR "Epithelial Cancer, Ovarian"[TW] OR "Ovarian Epithelial Cancers"[TW] OR "Ovarian Cancer, Epithelial"[TW] OR "Cancer, Epithelial Ovarian"[TW] OR "Epithelial Ovarian Cancers"[TW] OR "Ovarian Epithelial Carcinoma"[TW] OR "Epithelial Ovarian Carcinoma"[TW] OR "Carcinoma, Epithelial Ovarian"[TW] OR "Epithelial Ovarian Carcinomas"[TW] OR "Ovarian Carcinoma, Epithelial"[TW] OR "Serous Ovarian Cancer"[TW] OR "Endometrioid ovarian cancer"[TW] |  |
|  | **#9 Combine** | **#1 OR #2 OR #3 OR #4 OR #5 OR #6 OR #7 OR #8** |  |
|  | **#10** | **"serum inflammation marker"[TW] OR "Neutrophil-lymphocyte ratio"[TW] OR "NLR"[TW] OR "neutrophil-to-lymphocyte ratio"[TW] OR "monocyte lymphocyte ratio"[TW] OR "MLR"[TW] OR "monocyte-to-lymphocyte ratio"[TW] OR "lymphocyte monocyte ratio "[TW] OR "LMR"[TW] OR "lymphocyte-to-monocyte ratio"[TW] OR "platelet lymphocyte ratio"[TW] OR "PLR"[TW] OR "platelet-to-lymphocyte ratio"[TW] OR "systemic immune-inflammation index"[TW] OR "SII"[TW] OR "systemic immune‐inflammation index"[TW] OR "systemic inflammatory response index"[TW] OR "SIRI"[TW] OR "systemic inflammatory response markers"[TW]** |  |
|  | **#11 Combine** | **#9 AND #10** |  |
|  | **#12 Limit** | **#11 AND (meta-analysis[Filter] OR systematicreview[Filter])** |  |
|  |  |  |  |
|  |  |  |  |
| **DB** | **Search** | **Keywords** |  |
| **EMBASE** | #1 | "uterine cervix tumor"/exp |  |
|  | #2 | "Uterine Cervical Neoplasms":ti,ab,kw,de OR "Cervical Neoplasm, Uterine":ti,ab,kw,de OR "Neoplasm, Uterine Cervical":ti,ab,kw,de OR "Uterine Cervical Neoplasm":ti,ab,kw,de OR "Neoplasms, Cervical":ti,ab,kw,de OR "Cervical Neoplasms":ti,ab,kw,de OR "Cervical Neoplasm":ti,ab,kw,de OR "Neoplasms, Cervix":ti,ab,kw,de OR "Cervix Neoplasm":ti,ab,kw,de OR "Neoplasm, Cervix":ti,ab,kw,de OR "Cervix Neoplasms":ti,ab,kw,de OR "Cancer of the Uterine Cervix":ti,ab,kw,de OR "Cancer of the Cervix":ti,ab,kw,de OR "Cervical Cancer":ti,ab,kw,de OR "Cancer, Cervical":ti,ab,kw,de OR "Cervical Cancers":ti,ab,kw,de OR "Uterine Cervical Cancer":ti,ab,kw,de OR "Cancer, Uterine Cervical":ti,ab,kw,de OR "Cervical Cancer, Uterine":ti,ab,kw,de OR "Uterine Cervical Cancers":ti,ab,kw,de OR "Cancer of Cervix":ti,ab,kw,de OR "Cervix Cancer":ti,ab,kw,de OR "Cancer, Cervix":ti,ab,kw,de OR "cervical adenocarcinoma":ti,ab,kw,de |  |
|  | #3 | "ovary tumor"/exp |  |
|  | #4 | "Ovarian Neoplasms":ti,ab,kw,de OR "Neoplasm, Ovarian":ti,ab,kw,de OR "Ovarian Neoplasm":ti,ab,kw,de OR "Ovary Neoplasms":ti,ab,kw,de OR "Neoplasm, Ovary":ti,ab,kw,de OR "Neoplasms, Ovary":ti,ab,kw,de OR "Ovary Neoplasm":ti,ab,kw,de OR "Neoplasms, Ovarian":ti,ab,kw,de OR "Ovary Cancer":ti,ab,kw,de OR "Cancer, Ovary":ti,ab,kw,de OR "Cancers, Ovary":ti,ab,kw,de OR "Ovary Cancers":ti,ab,kw,de OR "Ovarian Cancer":ti,ab,kw,de OR "Cancer, Ovarian":ti,ab,kw,de OR "Cancers, Ovarian":ti,ab,kw,de OR "Ovarian Cancers":ti,ab,kw,de OR "Cancer of Ovary":ti,ab,kw,de OR "Cancer of the Ovary":ti,ab,kw,de OR "ovarian clear cell carcinoma ":ti,ab,kw,de OR "Mutinous ovarian carcinoma":ti,ab,kw,de |  |
|  | #5 | "endometrium tumor"/exp |  |
|  | #6 | "Endometrial Neoplasms":ti,ab,kw,de OR "Endometrial Neoplasm":ti,ab,kw,de OR "Neoplasm, Endometrial":ti,ab,kw,de OR "Neoplasms, Endometrial":ti,ab,kw,de OR "Endometrial Carcinoma":ti,ab,kw,de OR "Carcinoma, Endometrial":ti,ab,kw,de OR "Carcinomas, Endometrial":ti,ab,kw,de OR "Endometrial Carcinomas":ti,ab,kw,de OR "Endometrial Cancer":ti,ab,kw,de OR "Cancer, Endometrial":ti,ab,kw,de OR "Cancers, Endometrial":ti,ab,kw,de OR "Endometrial Cancers":ti,ab,kw,de OR "Endometrium Cancer":ti,ab,kw,de OR "Cancer, Endometrium":ti,ab,kw,de OR "Cancers, Endometrium":ti,ab,kw,de OR "Cancer of the Endometrium":ti,ab,kw,de OR "Carcinoma of Endometrium":ti,ab,kw,de OR "Endometrium Carcinoma":ti,ab,kw,de OR "Endometrium Carcinomas":ti,ab,kw,de OR "Cancer of Endometrium":ti,ab,kw,de OR "Endometrium Cancers":ti,ab,kw,de |  |
|  | #7 | "ovary carcinoma"/exp |  |
|  | #8 | "Carcinoma, Ovarian Epithelial":ti,ab,kw,de OR "Epithelial Carcinoma, Ovarian":ti,ab,kw,de OR "Ovarian Epithelial Carcinomas":ti,ab,kw,de OR "Epithelial Ovarian Cancer":ti,ab,kw,de OR "Ovarian Epithelial Cancer":ti,ab,kw,de OR "Cancer, Ovarian Epithelial":ti,ab,kw,de OR "Epithelial Cancer, Ovarian":ti,ab,kw,de OR "Ovarian Epithelial Cancers":ti,ab,kw,de OR "Ovarian Cancer, Epithelial":ti,ab,kw,de OR "Cancer, Epithelial Ovarian":ti,ab,kw,de OR "Epithelial Ovarian Cancers":ti,ab,kw,de OR "Ovarian Epithelial Carcinoma":ti,ab,kw,de OR "Epithelial Ovarian Carcinoma":ti,ab,kw,de OR "Carcinoma, Epithelial Ovarian":ti,ab,kw,de OR "Epithelial Ovarian Carcinomas":ti,ab,kw,de OR "Ovarian Carcinoma, Epithelial":ti,ab,kw,de OR "Serous Ovarian Cancer":ti,ab,kw,de OR "Endometrioid ovarian cancer":ti,ab,kw,de |  |
|  | **#9 Combine** | **#1 OR #2 OR #3 OR #4 OR #5 OR #6 OR #7 OR #8** |  |
|  | **#10** | **"serum inflammation marker":ti,ab,kw,de OR "Neutrophil-lymphocyte ratio":ti,ab,kw,de OR "NLR":ti,ab,kw,de OR "neutrophil-to-lymphocyte ratio":ti,ab,kw,de OR "monocyte lymphocyte ratio":ti,ab,kw,de OR "MLR":ti,ab,kw,de OR "monocyte-to-lymphocyte ratio":ti,ab,kw,de OR "lymphocyte monocyte ratio ":ti,ab,kw,de OR "LMR":ti,ab,kw,de OR "lymphocyte-to-monocyte ratio":ti,ab,kw,de OR "platelet lymphocyte ratio":ti,ab,kw,de OR "PLR":ti,ab,kw,de OR "platelet-to-lymphocyte ratio":ti,ab,kw,de OR "systemic immune-inflammation index":ti,ab,kw,de OR "SII":ti,ab,kw,de OR "systemic immune‐inflammation index":ti,ab,kw,de OR "systemic inflammatory response index":ti,ab,kw,de OR "SIRI":ti,ab,kw,de OR "systemic inflammatory response markers":ti,ab,kw,de** |  |
|  | **#11 Combine** | **#9 AND #10** |  |
|  | **#12 Limit** | **#11 AND ([systematic review]/lim OR [meta analysis]/lim)** |  |
|  |  |  |  |
|  |  |  |  |
| **DB** | **Search** | **Keywords** |  |
| **Cochrane Library** | #1 | [mh "Uterine Cervical Neoplasms"] |  |
|  | #2 | "Uterine Cervical Neoplasms":ti,ab,kw OR "Cervical Neoplasm, Uterine":ti,ab,kw OR "Neoplasm, Uterine Cervical":ti,ab,kw OR "Uterine Cervical Neoplasm":ti,ab,kw OR "Neoplasms, Cervical":ti,ab,kw OR "Cervical Neoplasms":ti,ab,kw OR "Cervical Neoplasm":ti,ab,kw OR "Neoplasms, Cervix":ti,ab,kw OR "Cervix Neoplasm":ti,ab,kw OR "Neoplasm, Cervix":ti,ab,kw OR "Cervix Neoplasms":ti,ab,kw OR "Cancer of the Uterine Cervix":ti,ab,kw OR "Cancer of the Cervix":ti,ab,kw OR "Cervical Cancer":ti,ab,kw OR "Cancer, Cervical":ti,ab,kw OR "Cervical Cancers":ti,ab,kw OR "Uterine Cervical Cancer":ti,ab,kw OR "Cancer, Uterine Cervical":ti,ab,kw OR "Cervical Cancer, Uterine":ti,ab,kw OR "Uterine Cervical Cancers":ti,ab,kw OR "Cancer of Cervix":ti,ab,kw OR "Cervix Cancer":ti,ab,kw OR "Cancer, Cervix":ti,ab,kw OR "cervical adenocarcinoma":ti,ab,kw |  |
|  | #3 | [mh "Ovarian Neoplasms"] |  |
|  | #4 | "Ovarian Neoplasms":ti,ab,kw OR "Neoplasm, Ovarian":ti,ab,kw OR "Ovarian Neoplasm":ti,ab,kw OR "Ovary Neoplasms":ti,ab,kw OR "Neoplasm, Ovary":ti,ab,kw OR "Neoplasms, Ovary":ti,ab,kw OR "Ovary Neoplasm":ti,ab,kw OR "Neoplasms, Ovarian":ti,ab,kw OR "Ovary Cancer":ti,ab,kw OR "Cancer, Ovary":ti,ab,kw OR "Cancers, Ovary":ti,ab,kw OR "Ovary Cancers":ti,ab,kw OR "Ovarian Cancer":ti,ab,kw OR "Cancer, Ovarian":ti,ab,kw OR "Cancers, Ovarian":ti,ab,kw OR "Ovarian Cancers":ti,ab,kw OR "Cancer of Ovary":ti,ab,kw OR "Cancer of the Ovary":ti,ab,kw OR "ovarian clear cell carcinoma ":ti,ab,kw OR "Mutinous ovarian carcinoma":ti,ab,kw |  |
|  | #5 | [mh "Endometrial Neoplasms"] |  |
|  | #6 | "Endometrial Neoplasms":ti,ab,kw OR "Endometrial Neoplasm":ti,ab,kw OR "Neoplasm, Endometrial":ti,ab,kw OR "Neoplasms, Endometrial":ti,ab,kw OR "Endometrial Carcinoma":ti,ab,kw OR "Carcinoma, Endometrial":ti,ab,kw OR "Carcinomas, Endometrial":ti,ab,kw OR "Endometrial Carcinomas":ti,ab,kw OR "Endometrial Cancer":ti,ab,kw OR "Cancer, Endometrial":ti,ab,kw OR "Cancers, Endometrial":ti,ab,kw OR "Endometrial Cancers":ti,ab,kw OR "Endometrium Cancer":ti,ab,kw OR "Cancer, Endometrium":ti,ab,kw OR "Cancers, Endometrium":ti,ab,kw OR "Cancer of the Endometrium":ti,ab,kw OR "Carcinoma of Endometrium":ti,ab,kw OR "Endometrium Carcinoma":ti,ab,kw OR "Endometrium Carcinomas":ti,ab,kw OR "Cancer of Endometrium":ti,ab,kw OR "Endometrium Cancers":ti,ab,kw |  |
|  | #7 | [mh "Carcinoma, Ovarian Epithelial"] |  |
|  | #8 | "Carcinoma, Ovarian Epithelial":ti,ab,kw OR "Epithelial Carcinoma, Ovarian":ti,ab,kw OR "Ovarian Epithelial Carcinomas":ti,ab,kw OR "Epithelial Ovarian Cancer":ti,ab,kw OR "Ovarian Epithelial Cancer":ti,ab,kw OR "Cancer, Ovarian Epithelial":ti,ab,kw OR "Epithelial Cancer, Ovarian":ti,ab,kw OR "Ovarian Epithelial Cancers":ti,ab,kw OR "Ovarian Cancer, Epithelial":ti,ab,kw OR "Cancer, Epithelial Ovarian":ti,ab,kw OR "Epithelial Ovarian Cancers":ti,ab,kw OR "Ovarian Epithelial Carcinoma":ti,ab,kw OR "Epithelial Ovarian Carcinoma":ti,ab,kw OR "Carcinoma, Epithelial Ovarian":ti,ab,kw OR "Epithelial Ovarian Carcinomas":ti,ab,kw OR "Ovarian Carcinoma, Epithelial":ti,ab,kw OR "Serous Ovarian Cancer":ti,ab,kw OR "Endometrioid ovarian cancer":ti,ab,kw |  |
|  | **#9 Combine** | **#1 OR #2 OR #3 OR #4 OR #5 OR #6 OR #7 OR #8** |  |
|  | **#10** | **"serum inflammation marker":ti,ab,kw OR "Neutrophil-lymphocyte ratio":ti,ab,kw OR "NLR":ti,ab,kw OR "neutrophil-to-lymphocyte ratio":ti,ab,kw OR "monocyte lymphocyte ratio":ti,ab,kw OR "MLR":ti,ab,kw OR "monocyte-to-lymphocyte ratio":ti,ab,kw OR "lymphocyte monocyte ratio ":ti,ab,kw OR "LMR":ti,ab,kw OR "lymphocyte-to-monocyte ratio":ti,ab,kw OR "platelet lymphocyte ratio":ti,ab,kw OR "PLR":ti,ab,kw OR "platelet-to-lymphocyte ratio":ti,ab,kw OR "systemic immune-inflammation index":ti,ab,kw OR "SII":ti,ab,kw OR "systemic immune‐inflammation index":ti,ab,kw OR "systemic inflammatory response index":ti,ab,kw OR "SIRI":ti,ab,kw OR "systemic inflammatory response markers":ti,ab,kw** |  |
|  | **#11 Combine** | **#9 AND #10** |  |
|  | **#12 Limit** | **Among #11, Cochrane Reviews (SR)** |  |

**Table S2**. The details of deviations from PROSPERO

| **Protocol method** | **Deviation from protocol method, with justification,** |  |
| --- | --- | --- |
|  | **First submission** | **Revision** |
| We planned to register to the PROSPERO. | The protocol was submitted to PROSPERO (submission number: 453021), but registration was declined because the subject of the protocol was not a priority. Consequently, a research note containing the PROSPERO content was created and shared with the entire research team. It was updated as the research progressed.  Type of deviation: Omission | There were no changes in the revised version compared with the initial submission. |
| The PICO keywords were predefined as follows and we requested the librarian (Najin Kim) to conduct a systematic search. P: Uterine Cervical Cancer, Ovarian Cancer, Endometrial Cancer, cervical adenocarcinoma, epithelial ovarian cancer, Cervix cancer, ovarian clear cell carcinoma, Serous Ovarian Cancer, Mutinous ovarian carcinoma, Endometrioid ovarian cancer  I: serum inflammation marker, NLR, MLR, LMR, PLR, SII, SIRI, neutrophil-to-lymphocyte ratio, monocyte-to-lymphocyte ratio, lymphocyte-to-monocyte ratio, platelet-to-lymphocyte ratio, systemic immune‐inflammation index, systemic inflammatory response index  C: None  O: prognosis, treatment, prognostic marker, survival, OS, PFS, DFS, Overall survival, Progressive-free survival, Disease-free survival, FIGO, International Federation of Gynecology and Obstetrics, prognostic factor, Therapeutic outcome, prognostic indicator, prognostic significance | First, with the librarian's assistance, we modified keywords to match MESH terms and conducted literature searches using constructed search formulas up to September 13, 2024. The actual keywords and search terms used are presented in the supplementary table.  Second, we found that some studies with survival data were excluded because of keywords related to the outcome category. This primarily occurred when studies were conducted primarily in cell lines or animal models with validation on actual patients, but data on prognosis was not mentioned in the title and abstract.  Consequently, we initiated the search process without the Outcome category to incorporate a more extensive body of literature, and subsequently, we proceeded according to the PICO keywords during the paper selection process.  Type of deviation: Modification | There were no changes in the revised version compared with the initial submission. |
| We planned to request additional data if the publication did not contain any suitable data for our research. | We contacted the authors of the relevant papers, but we were unable to obtain the necessary data. It is likely that most authors did not collect survival data because post- or dynamic PBIM already had sufficient difference between the benign and malignant groups, thereby leaving no necessity for survival data. The studies from which we requested data turned out to focus on PBIMs between the benign (including premalignant) and cancer groups. Since those studies focused on the difference in PBIMs between benign and malignant rather than on survival, it is likely that most authors did not collect survival data in the first place.  Type of deviation: Omission | We contacted them again during the revision period, but have not yet received a response.  Type of deviation: Omission |
| We investigated the post-treatment sampling time for the PBIMs. The sampling time was retrieved from each of the included studies, and based on clinical experience, it was classified as early vs. late sampling using 4 weeks as the cutoff. | The protocol was implemented as planned. | In agreement with the reviewer, we performed a meta-regression using sampling time in days as a continuous variable. Significant results were observed in sensitivity analyses at 2 weeks (p < 0.001), 4 weeks (p = 0.012), 6 weeks (p = 0.030), and 8 weeks (p = 0.030) in meta-regression. To identify a precise cutoff, we performed a natural spline regression to verify the nonlinear relationship between time in days and survival endpoints, and we confirmed a significant p-value of less than 0.001 at a cutoff of median 15 days.  Type of deviation: post hoc analysis & modification |
| We investigated the various methods for defining high PBIM in the studies including post-treatment and dynamic PBIM values. | The methods used to analyze PBIMs can be broadly classified as using either binary (equivalent to categorical) or continuous values.  Studies using binary values can be further divided into two categories for dynamic PBIMs: binary directional (increased or decreased post-treatment levels compared to pre-treatment levels) and binary threshold (above or below a cutoff).  The initial fixed-effects analysis revealed that the forest plots showed excessively large weights relative to sample size in studies using continuous variables. This led to the exclusion of these studies from subsequent analyses.  Type of deviation: post hoc analysis | In agreement with the reviewer, we re-performed the meta-analysis, including studies using continuous variables with standardization to per-standard deviation (SD) increase. Additionally, we conducted meta-regression and subgroup analyses to evaluate the influence of studies employing continuous values.  Type of deviation: post hoc analysis & modification |

**Table S3.** Workflow and codes for statistical analysis of (A) meta-regression, (B) non-linear regression, and (C) publication bias.

(A)

| # ===================================================================  # Workflow and R Codes for Meta-Regression Analyses  # ===================================================================  # --- 1. Load Required Packages ---  library(readxl); library(dplyr); library(stringr); library(metafor)  # --- 2. Data Loading and Preparation ---  # Modify file path as needed for your system.  path <- "C:/User/Desktop/Raw data.xlsx"  sheet <- "Sheet 1"  SR_data <- readxl::read_xlsx(path, sheet = sheet)  # Create the main analysis dataframe 'ana'  ana <- SR_data %>%  mutate(  # Convert numeric columns, coercing errors to NA  HR = suppressWarnings(as.numeric(HR)),  CI_low = suppressWarnings(as.numeric(CI_low)),  CI_high = suppressWarnings(as.numeric(CI_high)),  Days_Sampling = suppressWarnings(as.numeric(Days_Sampling)),  N = suppressWarnings(as.numeric(N)),    # Clean character-based moderator variables  Adjusted = str_to_title(str_trim(as.character(Adjusted))),  Class = str_to_title(str_trim(as.character(Class))),  PBIM = str_to_title(str_trim(as.character(PBIM))),  Endpoint = str_to_title(str_trim(as.character(Endpoint))),  Time_Analysis = str_to_title(str_trim(as.character(Time_Analysis))),  Site = str_to_title(str_trim(as.character(Site))),  Treatment = str_to_title(str_trim(as.character(Treatment))),  # Calculate effect size and variance  yi = log(HR),  sei = (log(CI_high) - log(CI_low)) / (2 * 1.96),  vi = sei^2,    # Create IDs for multi-level model  study_id = as.character(coalesce(Study_ID, as.character(row_number())))  ) %>%  group_by(study_id) %>%  mutate(effect_id = row_number()) %>%  ungroup() %>%    # Filter for studies with valid effect sizes and within the "Dynamic" subgroup  filter(is.finite(yi), is.finite(vi), vi > 0,  Time_Analysis == "Dynamic") %>%    # Define time-based subgroup  mutate(  Time_Group = ifelse(Days_Sampling <= 15, "Early", "Late")  )  # --- 3. Define a Function for Meta-Regression and R² Calculation ---  # This function avoids code repetition and ensures consistency.  run_metareg <- function(mod_formula, mod_name, data) {    # Fit the null model once to get baseline heterogeneity  fit_null <- rma.mv(yi, vi,  random = ~ 1 \| study_id / effect_id,  method = "REML",  data = data)    # Fit the moderator model  # Using a simpler random-effects structure for study-level covariates  fit_mod <- rma.mv(yi, vi,  mods = mod_formula,  random = ~ 1 \| study_id,  method = "REML",  data = data)    # Print the model summary  cat(paste0("\n\n--- Meta-Regression for Moderator: ", mod_name, " ---\n"))  print(summary(fit_mod))    # Calculate and print R-squared  tau2_null <- fit_null$sigma2[1]  tau2_mod <- fit_mod$sigma2[1]    if (tau2_null > 0) {  R_squared <- 100 * (tau2_null - tau2_mod) / tau2_null  cat("R-squared for the", mod_name, "variable:", round(R_squared, 2), "%\n")  } else {  cat("R-squared for the", mod_name, "variable: Not applicable (baseline tau² is zero).\n")  }    return(fit_mod)  }  # --- 4. Run Analyses for Each Moderator ---  # Note: Ensure the factor levels are correct before running models.  # The code below assumes there are at least two levels for each factor.  # If an error occurs, check the variable with `table(ana$VariableName)`.  # Moderator: Site  fit_Site <- run_metareg(mod_formula = ~ factor(Site), mod_name = "Site", data = ana)  # Moderator: Treatment  fit_Treatment <- run_metareg(mod_formula = ~ factor(Treatment), mod_name = "Treatment", data = ana)  # Moderator: Days_Sampling (as a continuous variable)  fit_Days_Sampling <- run_metareg(mod_formula = ~ Days_Sampling, mod_name = "Days_Sampling (continuous)", data = ana)  # Moderator: Time_Group (Early vs. Late)  fit_Time_Group <- run_metareg(mod_formula = ~ factor(Time_Group), mod_name = "Time_Group (binary)", data = ana)  # Moderator: Adjusted  fit_Adjusted <- run_metareg(mod_formula = ~ factor(Adjusted), mod_name = "Adjusted", data = ana)  # Moderator: Class  fit_Class <- run_metareg(mod_formula = ~ factor(Class), mod_name = "Class", data = ana) |
| --- |

(B)

| # ===================================================================  # Workflow and R Codes for Non-linear Meta-Regression and Visualization  # ===================================================================  # --- 1. Load Required Packages ---  library(readxl)  library(dplyr)  library(splines)  library(metafor)  library(meta)  library(ggplot2)  library(purrr)  # --- 2. Data Loading and Preparation ---  # Modify file path as needed for your system.  path <- "C:/User/Desktop/Raw data.xlsx"  sheet <- "Sheet 1"  SR_data <- readxl::read_xlsx(path, sheet = sheet)  # Create the main analysis dataframe 'ana'  ana <- SR_data %>%  mutate(  HR = suppressWarnings(as.numeric(HR)),  CI_low = suppressWarnings(as.numeric(CI_low)),  CI_high = suppressWarnings(as.numeric(CI_high)),  Days_Sampling = suppressWarnings(as.numeric(Days_Sampling)),  yi = log(HR),  sei = (log(CI_high) - log(CI_low)) / (2 * 1.96),  vi = sei^2,  study_id = as.character(coalesce(Study_ID, as.character(row_number()))),  Days_Sampling_c = Days_Sampling - mean(Days_Sampling, na.rm = TRUE)  ) %>%  group_by(study_id) %>%  mutate(effect_id = row_number()) %>%  ungroup() %>%  filter(is.finite(yi), is.finite(sei), sei > 0)  # --- 3. Fit Natural Spline Meta-Regression Model (df = 2) ---  fit_spline_ns_df2 <- rma.mv(yi, vi,  mods = ~ ns(Days_Sampling_c, df = 2),  random = ~ 1 \| study_id/effect_id,  data = ana)  print(summary(fit_spline_ns_df2))  # --- 4. Prediction for Visualization ---  pred_days_c <- seq(min(ana$Days_Sampling_c, na.rm = TRUE),  max(ana$Days_Sampling_c, na.rm = TRUE),  length.out = 100)  # Must use coefficient names from the df=2 model.  newmods_ns_matrix <- ns(pred_days_c, df = 2)  colnames(newmods_ns_matrix) <- names(coef(fit_spline_ns_df2))[-1]  preds_spline <- predict(fit_spline_ns_df2, newmods = newmods_ns_matrix)  mean_days <- mean(SR_data$Days_Sampling, na.rm = TRUE)  df_pred_spline <- data.frame(  Days_Sampling_c = pred_days_c,  pred_log = preds_spline$pred,  ci.lb_log = preds_spline$ci.lb,  ci.ub_log = preds_spline$ci.ub  ) %>%  mutate(Days_Sampling = Days_Sampling_c + mean_days)  # --- 5. Subgroup Analysis for Plotting by Seting Data-Driven Cut-off and Creating Groups ---  # 5a. Find the internal knot location from the Natural Spline (df=2) model.  # This location serves as the data-driven statistical inflection point.  spline_object_df2 <- ns(ana$Days_Sampling_c, df = 2)  # As df=2, there is only one internal knot. Extract the value using [1].  internal_knot_c <- attr(spline_object_df2, "knots")[1]  mean_days <- mean(SR_data$Days_Sampling, na.rm = TRUE)  data_driven_cutoff <- internal_knot_c + mean_days  # Check the calculated cut-off value.  cat("Data-Driven Cut-off (Knot Location):", round(data_driven_cutoff, 1), "days\n")  # 5b. Create "Early" and "Late" groups based on the data_driven_cutoff value found above.  ana <- ana %>%  mutate(  Phase = case_when(  Days_Sampling <= data_driven_cutoff ~ "Early",  Days_Sampling > data_driven_cutoff ~ "Late"  ) %>%  factor(levels = c("Early", "Late"))  )  summary_stats <- ana %>%  group_by(Phase) %>%  summarise(  meta_res = list(metagen(TE = yi, seTE = sei, sm = "HR", data = pick(everything()))),  .groups = 'drop'  ) %>%  mutate(  logHR = map_dbl(meta_res, ~ .$TE.random),  CI_low = map_dbl(meta_res, ~ .$lower.random),  CI_high = map_dbl(meta_res, ~ .$upper.random),  p_val = map_dbl(meta_res, ~ .$pval.random),  label = paste0(  Phase,  "\nlog(HR) = ", format(round(logHR, 2), nsmall = 2),  "\n95% CI: [", format(round(CI_low, 2), nsmall = 2), ", ", format(round(CI_high, 2), nsmall = 2), "]",  "\np = ", format(round(p_val, 3), nsmall = 3)  ),  x_position = case_when(  Phase == "Early" ~ 7,  Phase == "Late" ~ 56  ),  y_position = case_when(  Phase == "Early" ~ 3.3,  Phase == "Late" ~ 2.5  )  )  # --- 6. Generate Text for Overall Trend p-value ---  qm_df <- fit_spline_ns_df2$QMdf[1]  qm_val <- round(fit_spline_ns_df2$QM, 2)  p_val_overall <- fit_spline_ns_df2$QMp  p_text_overall <- ifelse(p_val_overall < 0.001, "p < 0.001", paste0("p = ", round(p_val_overall, 3)))  final_text_regression <- paste0("Overall non-linear trend:\nQM(df=", qm_df, ") = ", qm_val, ", ", p_text_overall)  # --- 7. Generate Final Graph ---  ana$w <- 1 / sqrt(ana$vi)  ggplot() +  geom_hline(yintercept = 0, linetype = "dashed", color = "grey50") +  geom_point(data = ana, aes(x = Days_Sampling, y = yi, size = w),  shape = 21, fill = "gray70", alpha = 0.5) +  geom_line(data = df_pred_spline, aes(x = Days_Sampling, y = pred_log),  color = "darkred", linewidth = 1.2) +  geom_ribbon(data = df_pred_spline, aes(x = Days_Sampling, ymin = ci.lb_log, ymax = ci.ub_log),  fill = "red", alpha = 0.15) +  geom_vline(xintercept = knot_location, linetype = "dotted", color = "blue", linewidth = 1) +  geom_text(data = summary_stats,  aes(x = x_position, y = y_position, label = label),  hjust = 0.5, fontface = "bold", size = 4, vjust = 1) +  scale_size_continuous(  name = "Weight",  breaks = c(10, 250, 750)) +  labs(  title = "Non-linear Relationship over Time",  x = "Post-treatment Sampling Time (Days)",  y = "log HR"  ) +  theme_bw(base_size = 14) +  scale_x_continuous(breaks = seq(0, max(ana$Days_Sampling, na.rm = TRUE) + 7, by = 7)) +  coord_cartesian(ylim = c(min(ana$yi, na.rm = TRUE), 3.5)) +  theme(axis.text.x = element_text(angle = 0, hjust = 0.5)) +  annotate(  "text",  x = Inf, y = Inf,  label = final_text_regression,  hjust = 1.05, vjust = 1.1,  size = 4.5, fontface = "bold"  ) |
| --- |

(C)

| # ===================================================================  # Workflow and Python Codes for Publication Bias Analysis  # ===================================================================  # --- 1. Import Required Packages ---  import os  import glob  import numpy as np  import pandas as pd  from scipy import stats  import matplotlib.pyplot as plt  from copy import deepcopy  # --- 2. Define Global Variables and Helper Functions ---  # Define input and output directories  # NOTE: Modify these paths for your system.  IN_DIR = "path/to/your/excel/folder" # Folder containing the .xlsx file  OUT_DIR = "path/to/your/output/folder" # Folder to save plots  os.makedirs(OUT_DIR, exist_ok=True)  def trim_and_fill(yi, sei, side="right"):  """  Duval & Tweedie's Trim-and-Fill method (simplified implementation).  Args:  yi (array): Array of log effect sizes (e.g., log HR).  sei (array): Array of standard errors.  side (str): The side of the funnel plot to trim ("right" or "left").  Returns:  A dictionary containing the number of filled studies (k0),  the original and adjusted pooled effects, and the filled data.  """  yi = np.asarray(yi, dtype=float)  sei = np.asarray(sei, dtype=float)  w = 1.0 / (sei**2)  # Original pooled estimate (fixed-effect)  pooled = np.sum(w * yi) / np.sum(w)  # Sort by effect size  order = np.argsort(yi)  yi_sorted, sei_sorted = yi[order], sei[order]  # Estimate the number of missing studies (k0)  k0 = 0  if side == "right":  left = np.sum(yi_sorted < pooled)  right = np.sum(yi_sorted >= pooled)  k0 = max(0, right - left - 1) # Classic estimator R0  else: # side == "left"  right = np.sum(yi_sorted > pooled)  left = np.sum(yi_sorted <= pooled)  k0 = max(0, left - right -1) # Classic estimator L0  # Impute the missing studies by mirroring the most extreme values  yi_filled = list(yi)  sei_filled = list(sei)  if k0 > 0:  if side == "right":  extreme_yi = yi_sorted[-k0:]  extreme_sei = sei_sorted[-k0:]  mirrored_yi = 2 * pooled - extreme_yi  else: # side == "left"  extreme_yi = yi_sorted[:k0]  extreme_sei = sei_sorted[:k0]  mirrored_yi = 2 * pooled - extreme_yi    yi_filled.extend(mirrored_yi)  sei_filled.extend(extreme_sei) # SEs are not mirrored  # Calculate the adjusted pooled estimate  w_filled = 1.0 / (np.array(sei_filled)**2)  pooled_filled = np.sum(w_filled * np.array(yi_filled)) / np.sum(w_filled)  return {  "k0": k0,  "pooled_orig": pooled,  "pooled_filled": pooled_filled,  "yi_filled": np.array(yi_filled),  "sei_filled": np.array(sei_filled)  }  def funnel_plot_with_trim_fill(yi, sei, title, save_path):  """  Generates a funnel plot and overlays trim-and-fill results.  """  yi = np.asarray(yi, dtype=float)  sei = np.asarray(sei, dtype=float)  # Perform trim-and-fill analysis  tf_res = trim_and_fill(yi, sei, side="left") # Assuming smaller studies are missing  pooled_orig = tf_res["pooled_orig"]  # Generate funnel guides  se_max = np.nanmax(sei) * 1.1  se_grid = np.linspace(0, se_max, 100)  upper_ci = pooled_orig + 1.96 * se_grid  lower_ci = pooled_orig - 1.96 * se_grid  # Create plot  plt.figure(figsize=(8, 6))  plt.scatter(yi, sei, s=30, label="Observed studies", facecolors='none', edgecolors='black')  if tf_res["k0"] > 0:  # Identify the filled-in studies for plotting  n_orig = len(yi)  yi_imputed = tf_res["yi_filled"][n_orig:]  sei_imputed = tf_res["sei_filled"][n_orig:]  plt.scatter(yi_imputed, sei_imputed, s=40, c="black", marker="D", label="Imputed studies")  plt.axvline(pooled_orig, linestyle="--", color="gray", label=f"Original Pooled Effect = {pooled_orig:.2f}")  if tf_res["k0"] > 0:  plt.axvline(tf_res["pooled_filled"], linestyle=":", color="black", label=f"Adjusted Pooled Effect = {tf_res['pooled_filled']:.2f}")    plt.plot(upper_ci, se_grid, linestyle=":", color="gray")  plt.plot(lower_ci, se_grid, linestyle=":", color="gray")    plt.gca().invert_yaxis() # Invert y-axis to show most precise studies at the top  plt.xlabel("Effect Size (log HR)")  plt.ylabel("Standard Error")  plt.title(f"{title}\nTrim-and-Fill: k₀ = {tf_res['k0']} studies imputed")  plt.legend()  plt.tight_layout()  plt.savefig(save_path, dpi=300)  plt.close()  def egger_test(yi, sei):  """  Performs Egger's regression test for funnel plot asymmetry.  """  precision = 1.0 / sei  snd = yi / sei # Standardized Normal Deviate    # Weighted least squares regression: snd ~ precision  X = np.column_stack([np.ones_like(precision), precision])  try:  beta_hat, _, _, _ = np.linalg.lstsq(X, snd, rcond=None)  y_hat = X @ beta_hat  residuals = snd - y_hat  n, p = X.shape  if n <= p: return None # Cannot compute if k <= 2    sigma2 = np.sum(residuals**2) / (n - p)  cov_matrix = sigma2 * np.linalg.inv(X.T @ X)  se_intercept = float(np.sqrt(cov_matrix[0, 0]))    t_intercept = float(beta_hat[0] / se_intercept)  p_intercept = float(2 * stats.t.sf(np.abs(t_intercept), df=n - p))  return {  "intercept": float(beta_hat[0]),  "slope": float(beta_hat[1]),  "p_value": p_intercept,  "precision": precision,  "snd": snd  }  except np.linalg.LinAlgError:  return None  def egger_plot(egger_res, title, save_path):  """  Generates a regression plot for Egger's test.  """  if egger_res is None: return    precision = egger_res["precision"]  snd = egger_res["snd"]  intercept = egger_res["intercept"]  slope = egger_res["slope"]  p_value = egger_res["p_value"]  x_line = np.linspace(np.min(precision), np.max(precision), 100)  y_line = intercept + slope * x_line    plt.figure(figsize=(8, 6))  plt.scatter(precision, snd, s=30, facecolors='none', edgecolors='black')  plt.plot(x_line, y_line, linestyle="--", color="gray")  plt.xlabel("Precision (1 / SE)")  plt.ylabel("Standard Normal Deviate (log HR / SE)")  plt.title(f"{title}\nEgger's Test Intercept = {intercept:.2f} (p = {p_value:.3f})")  plt.tight_layout()  plt.savefig(save_path, dpi=300)  plt.close()  # --- 3. Main Analysis Workflow ---  # Load the data  # NOTE: Ensure file path and sheet name are correct.  file_path = "C:/Users/User/Desktop/SR/Raw data_250917.xlsx"  sheet_name = "Fill_report__v2_" # This was the last used sheet name  df = pd.read_excel(file_path, sheet_name=sheet_name)  # --- Define subgroups to analyze ---  # This structure makes it easy to add or remove analyses.  subgroups_to_analyze = [  {"PBIM": "NLR", "Endpoint": "OS"},  {"PBIM": "PLR", "Endpoint": "OS"},  {"PBIM": "NLR", "Endpoint": "PFS"},  # Add other subgroups as needed...  ]  # Loop through each defined subgroup to perform analyses  for subgroup in subgroups_to_analyze:  pbim = subgroup["PBIM"]  endpoint = subgroup["Endpoint"]    # Create a title for this subgroup's analysis  analysis_title = f"Publication Bias for {pbim} - {endpoint}"  print(f"\n--- Running Analysis for: {pbim} - {endpoint} ---")    # Filter data for the current subgroup  df_sub = df[(df['PBIM'] == pbim) & (df['Endpoint'] == endpoint)].copy()    # Prepare data: convert to numeric and drop rows with missing effect sizes/SEs  df_sub["yi"] = pd.to_numeric(np.log(df_sub["HR"]), errors='coerce')  df_sub["sei"] = pd.to_numeric((np.log(df_sub["CI_high"]) - np.log(df_sub["CI_low"])) / (2 * 1.96), errors='coerce')  df_sub = df_sub.dropna(subset=["yi", "sei"])    if len(df_sub) < 2:  print(f"Skipping {pbim}-{endpoint}: Not enough studies (k={len(df_sub)})")  continue  yi = df_sub["yi"].values  sei = df_sub["sei"].values  # a) Generate Funnel Plot with Trim-and-Fill  funnel_plot_with_trim_fill(  yi=yi, sei=sei,  title=analysis_title,  save_path=os.path.join(OUT_DIR, f"Funnel_TrimFill_{pbim}_{endpoint}.png")  )    # b) Perform and Plot Egger's Test  egger_result = egger_test(yi=yi, sei=sei)  if egger_result:  print(f"Egger's Test: p-value = {egger_result['p_value']:.4f}")  egger_plot(  egger_res=egger_result,  title=analysis_title,  save_path=os.path.join(OUT_DIR, f"Egger_Plot_{pbim}_{endpoint}.png")  )  else:  print("Egger's Test could not be performed (k is too small).")  print("\n--- All analyses complete. ---") |
| --- |

**Table S4**. Main Characteristics of all gynecological cancer studies included in the meta-analysis

| Site | Authors, year | Country | Ethnicity | Age (years) | Enrollment period | Follow up (months) | Number of events | Statistical approach | covariates adjusted |
| --- | --- | --- | --- | --- | --- | --- | --- | --- | --- |
| Cervix | Chao et al., 2020 (19) | China | Asian | 42(28-79) ¹ | 2009-2018 | 67(6-129) ¹ | NA | Univariate analysis | NA |
|  | Kim et al., 2018 (20) | Korea | Asian | 55(26-84) ¹ | 2009-2016 | 39.9(2.7-114.6) ¹ | 23 deaths,  47 recurrences | Multivariate analysis | age, histology, ASA, CA-125, stage, residual disease, total cycles of chemotherapy. |
|  | Lee et al., 2020 (21) | Korea | Asian | 53.67±13.23 ² | 2005-2016 | 50(6-124) ¹ | 24 deaths,  45 recurrences | Multivariate analysis | NA ^3^ |
|  | Trinh et al., 2020 (22) | USA | African American | 47(22-85) ¹ | 2008-2019 | 40.4(27.5-54.5) ¹ | 34 deaths,  44 recurrences | Multivariate analysis | stage, histology. |
|  | Du et al., 2023 (23) | China | Asian | 52.38(24-76) ¹ | 2012-2017 | 71.5(5-132) ¹ | 25 deaths,  45 recurrences | Univariate analysis | NA |
|  | Chen et al., 2024 (24) | Taiwan | Asian | 60.1±14.1 ² | 2016-2021 | 33.8(7.2-79.3) ¹ | 27 deaths,  46 recurrences | Univariate analysis | NA |
|  | Lee et al., 2025 (25) | Korea | Asian | 60 ¹ | 2010-2022 | 28¹ | 29 deaths,  22 recurrences | Multivariate analysis | age, ECOG, stage |
| Ovary | Kim et al., 2020 (26) | Korea | Asian | 57(27-80) ¹ | 2007-2015 | NR | NA | Multivariate analysis | age, stage |
|  | Sanna et al., 2021 (27) | Italy | Caucasian | 57(39-86) ¹ | 2009-2019 | NR | NA | Univariate analysis | NA |
|  | Plaja et al., 2023 (28) | Spain | Caucasian | 62 ¹ | 2008-2016 | 46.9(6.8-126.2) ¹ | 85 deaths,  117 recurrences | Univariate analysis | NA |
|  | Weng et al., 2023 (29) | Taiwan | Asian | 53(23-83) ¹ | 2010-2019 | 62.4(13.2-135.6) ¹ | 158 deaths,  208 recurrences | Multivariate analysis | ECOG, stage, histologic grade, residual disease, SMI, SMD, albumin, PNI |
|  | Lazar et al., 2024 (30) | Romania | Caucasian | 65.4±5.43 ² | 2016-2024 | NR | NA | Multivariate analysis | NA ^3^ |
| Endometrium | Ding et al., 2017 (31) | China | Asian | 59.29(33-82) ¹ | 2007-2013 | 65.84(1-114) ¹ | 31 deaths,  47 recurrences | Multivariate analysis | NA ^3^ |
|  | Huang et al., 2021 (32) | China | Asian | 54.17(25-75) ¹ | 2010-2015 | 89.62(23-125) ¹ | 8 deaths | Multivariate analysis | NA ^3^ |

**¹** Values are presented as either median (range) or mean; **²** values are mean ± standard deviation (SD). ^3^ The manuscript does not specify the exact statistical methods employed or the covariates included in the adjustment. Abbreviations: NA, not available; ASA, American Society of Anesthesiologists; ECOG, Eastern Cooperative Oncology Group; SMI, skeleton muscle index; SMD, skeleton muscle radiodensity, PNI, prognostic nutritional index.

**Table S5**. Thresholds of all gynecological cancer studies included in the Post group.

| **Study** | **Survival** | **Marker** | **Threshold** |
| --- | --- | --- | --- |
| Lee et al., 2020 (21) | OS | NLR | post > 5.231 |
|  |  | PLR | post < 2.235 |
|  |  | MLR | post > 368 |
|  | DFS | NLR | post > 3.76 |
|  |  | PLR | post < 2.31 |
|  |  | MLR | post > 356.6 |
| Du et al., 2023 (23) | OS, PFS | NLR | post > 2.08 |
| Lee et al., 2025 (25) | OS | NLR | post > 3.554 |
|  |  | PLR | post > 2.15 |
|  |  | MLR | post > 0.5103 |
|  |  | SII | post > 3.01 |
|  |  | SIRI | post > 4 .89 |
|  | DFS | NLR | post > 2.7319 |
|  |  | PLR | post > 441.0585 |
|  |  | MLR | post > 0.6053 |
|  |  | SII | post > 885504 |
|  |  | SIRI | post > 1847.56 |
| Sanna et al., 2021 (27) | PFS | NLR | post > 1.58 |
| Ding et al., 2017 (31) | OS, DFS | NLR | post > 7.54 |
|  |  | PLR | post > 186.4 |
| Huang et al., 2021 (32) | OS | SII | post > 2.93E+12 |

**Table S6.** Thresholds of all gynecological cancer studies included in the Dynamic group

| **Study** | **Survival** | **Marker** | **Threshold** |
| --- | --- | --- | --- |
| Chao et al., 2020 (19) | OS | SIRI | post/pre > 1.75 |
| Kim et al., 2018 (20) | OS, PFS | NLR | post >pre |
| Lee et al., 2020 (21) | OS | NLR | post/pre > 1.25 |
|  |  | PLR | post/pre < 0.548 |
|  |  | MLR | post/pre > 1.724 |
|  | DFS | NLR | post/pre > 1.25 |
|  |  | PLR | post/pre < 0.389 |
|  |  | MLR | post/pre > 1.63 |
| Du et al., 2023 (23) | OS, PFS | NLR | post>pre |
| Chen et al., 2024 (24) | OS, PFS | NLR | post>pre |
|  |  | PLR | post>pre |
|  |  | MLR | post>pre |
| Lee et al., 2025 (25) | OS | NLR | post/pre > 1.33 |
|  |  | PLR | post/pre > 1.09 |
|  |  | MLR | post/pre > 3.10 |
|  |  | SII | post/pre > 1.15 |
|  |  | SIRI | post/pre > 3.77 |
|  | DFS | NLR | post/pre > 1.3728 |
|  |  | PLR | post/pre > 3.5411 |
|  |  | MLR | post/pre > 0.8115 |
|  |  | SII | post/pre > 0.8529 |
|  |  | SIRI | post/pre > 2.6472 |
| Kim et al., 2020 (26) | OS, PFS | NLR | post>pre |
| Plaja et al., 2023 (28) | OS, PFS | NLR | post>pre |
|  |  | PLR | post>pre |
|  |  | MLR | post>pre |
| Lazar et al., 2024 (30) | OS, PFS | NLR | post>pre |

**Table S7.** Meta-regression analyses exploring between-study heterogeneity by cancer site, treatment modality, hazard ratio adjustment and sampling time.

| Moderator | R² (%) | *p*-value |
| --- | --- | --- |
| Cancer site | -8.17 | 0.4610 |
| Treatment (CCRT vs. Surgery) | -8.51 | 0.6726 |
| Adjusted vs. Unadjusted | -0.87 | 0.6558 |
| Categorical vs. Continuous | 8.12 | 0.1441 |
| Sampling Time (Days) | 36.76 | 0.0042 |
| Sampling Time (median days ≤15 vs. >15) | 65.62 | <0.0001 |
| **R²**: Percentage of between-study heterogeneity explained by the moderator | | |

.

**Table S8.** Characteristics of patients clinicopathological data included in the meta-analysis.

| Clinicopathological parameters | | Du et al. (23) | | Ding et al. (31) | | Huang et al. (32) | |
| --- | --- | --- | --- | --- | --- | --- | --- |
|  |  | Low PBIM | High PBIM | Low PBIM | High PBIM | Low PBIM | High PBIM |
| Age | Young | 29 | 56 | 56 | 37 | 109 | 5 |
|  | Old | 47 | 69 | 64 | 28 | 130 | 1 |
| Histology (Endometrium) | Type Ⅰ | - | - | 110 | 53 | 223 | 5 |
|  | Type Ⅱ | - | - | 10 | 12 | 16 | 1 |
| FIGO Stage | Low | 56 | 91 | 79 | 38 | 206 | 4 |
|  | High | 20 | 34 | 13 | 18 | 33 | 2 |
| Lymphatic Invasion | Negative | 67 | 101 | 84 | 45 | 223 | 4 |
|  | Positive | 9 | 24 | 8 | 11 | 16 | 2 |
| Depth of Invasion | Superficial | 44 | 71 | - | - | 202 | 4 |
|  | Deep | 32 | 54 | - | - | 37 | 2 |
| Operative approach | Laparoscopy | - | - | 44 | 27 | 49 | 1 |
|  | Laparotomy | - | - | 76 | 38 | 190 | 5 |
| Postoperative  Chemotherapy | Negative | - | - | 53 | 27 | 193 | 5 |
|  | Positive | - | - | 67 | 38 | 46 | 1 |
| Postoperative  Radiotherapy | Negative | - | - | 118 | 62 | 212 | 5 |
|  | Positive | - | - | 2 | 3 | 27 | 1 |
| Menopausal Status | Premenopausal | 37 | 64 | - | - | 98 | 6 |
|  | Postmenopausal | 39 | 61 | - | - | 140 | 0 |
| Hypertension | Negative | - | - | 83 | 47 | 151 | 4 |
|  | Positive | - | - | 37 | 18 | 88 | 2 |
| Diabetes | Negative | - | - | 99 | 59 | 201 | 4 |
|  | Positive | - | - | 21 | 6 | 38 | 2 |
